# Whole-genome bisulfite sequencing of multiple individuals reveals complementary roles of promoter and gene body methylation in transcriptional regulation

**DOI:** 10.1186/s13059-014-0408-0

**Published:** 2014-07-30

**Authors:** Shaoke Lou, Heung-Man Lee, Hao Qin, Jing-Woei Li, Zhibo Gao, Xin Liu, Landon L Chan, Vincent KL Lam, Wing-Yee So, Ying Wang, Si Lok, Jun Wang, Ronald CW Ma, Stephen Kwok-Wing Tsui, Juliana CN Chan, Ting-Fung Chan, Kevin Y Yip

**Affiliations:** Department of Computer Science and Engineering, The Chinese University of Hong Kong, Shatin, New Territories Hong Kong; School of Life Sciences, The Chinese University of Hong Kong, Shatin, New Territories Hong Kong; Department of Medicine and Therapeutics, The Chinese University of Hong Kong, Shatin, New Territories Hong Kong; Hong Kong Institute of Diabetes and Obesity, The Chinese University of Hong Kong, Shatin, New Territories Hong Kong; Li Ka Shing Institute of Health Sciences, The Chinese University of Hong Kong, Shatin, New Territories Hong Kong; Beijing Genomics Institute (BGI)-Shenzhen, Shenzhen, China; Department of Chemical Pathology, The Chinese University of Hong Kong, Shatin, New Territories Hong Kong; Department of Biology, University of Copenhagen, Copenhagen, Denmark; The Novo Nordisk Foundation Center for Basic Metabolic Research, University of Copenhagen, Copenhagen, Denmark; King Abdulaziz University, Jeddah, Saudi Arabia; School of Biomedical Sciences, The Chinese University of Hong Kong, Shatin, New Territories Hong Kong; Hong Kong Bioinformatics Centre, The Chinese University of Hong Kong, Shatin, New Territories Hong Kong; CUHK-BGI Innovation Institute of Trans-omics, The Chinese University of Hong Kong, Shatin, New Territories Hong Kong

## Abstract

**Background:**

DNA methylation is an important type of epigenetic modification involved in gene regulation. Although strong DNA methylation at promoters is widely recognized to be associated with transcriptional repression, many aspects of DNA methylation remain not fully understood, including the quantitative relationships between DNA methylation and expression levels, and the individual roles of promoter and gene body methylation.

**Results:**

Here we present an integrated analysis of whole-genome bisulfite sequencing and RNA sequencing data from human samples and cell lines. We find that while promoter methylation inversely correlates with gene expression as generally observed, the repressive effect is clear only on genes with a very high DNA methylation level. By means of statistical modeling, we find that DNA methylation is indicative of the expression class of a gene in general, but gene body methylation is a better indicator than promoter methylation. These findings are general in that a model constructed from a sample or cell line could accurately fit the unseen data from another. We further find that promoter and gene body methylation have minimal redundancy, and either one is sufficient to signify low expression. Finally, we obtain increased modeling power by integrating histone modification data with the DNA methylation data, showing that neither type of information fully subsumes the other.

**Conclusion:**

Our results suggest that DNA methylation outside promoters also plays critical roles in gene regulation. Future studies on gene regulatory mechanisms and disease-associated differential methylation should pay more attention to DNA methylation at gene bodies and other non-promoter regions.

**Electronic supplementary material:**

The online version of this article (doi:10.1186/s13059-014-0408-0) contains supplementary material, which is available to authorized users.

## Background

DNA methylation refers to the methylation of the carbon atom at position 5 of a cytosine (m5C), which mostly happens within CpG, CpHpG and CpHpH nucleotide patterns in eukaryotes [1-4]. In differentiated cells of mammals, methylation appears predominantly at CpG dinucleotides, with about 60% to 90% of all CpG sites methylated [4-6]. DNA methylation is a stable epigenetic modification involved in many cellular processes, including cellular differentiation, suppression of transposable elements, embryogenesis, X-inactivation and genomic imprinting [4]. DNA methylation around the 5’ terminus of a gene is well-recognized to be associated with low gene expression, by actively repressing transcription or marking already silenced genes [7,8]. Different models have been proposed for the molecular mechanisms of DNA methylation in transcriptional repression, including the blockage of transcription factor binding, and the recruitment of transcriptional repressors involved in methylation-dependent chromatin remodeling and gene repression [1,9]. The important roles of DNA methylation are also evidenced by the association of aberrant DNA methylation with various human diseases [10,11].

### Previous findings obtained by high-throughput methods

To systematically study DNA methylation at the genomic scale, it is necessary to identify many, ideally all, methylated sites in a genome. Various high-throughput methods have been invented for large-scale detection of methylation events [8,12-14]. These methods differ in the way genomic regions enriched for methylated or unmethylated DNA are identified, and how genomic locations of these regions or their sequences are determined. The former includes the use of methylation-sensitive restriction enzyme digestion [15,16], immunoprecipitation [17-19], affinity capture [20,21], and bisulfite conversion of unmethylated cytosines to uracils [2-4,22]. The identities of the collected regions are determined by microarray [15-19] or sequencing [2-4,20-22]. These methods have been extensively compared in terms of their genomic coverage, resolution, cost, consistency and context-specific bias [23,24].

By integrating gene expression data and global DNA methylation profiles from these high-throughput methods, a general genome-wide negative correlation between promoter methylation and gene expression was observed in multiple species [25,26]. However, substantial overlap exists in the distributions of promoter methylation level between genes with low versus high expression [19,25,26]. It was also suggested that for CpG island promoters, DNA methylation is sufficient but not necessary for their inactivation, while for promoters with low CpG content, hypermethylation does not preclude gene expression [19]. The quantitative relationship between promoter methylation and gene expression is thus more complicated than once assumed [14] and the details have not been fully worked out.

The high-throughput methods have also provided evidence that there is extensive DNA methylation at transcribable regions [27]. Gene body methylation was observed to be positively correlated with gene expression in some cell types [28,29], but not in others [4]. It was suggested that the positive correlation could either be due to *de novo* methylation of internal CpG islands facilitated by transcription, in which case methylation was the consequence; or due to the repression of anti-sense transcripts that would down-regulate expression of the sense transcript, in which case methylation was the cause [29]. In contrast, it was also previously proposed that intragenic DNA methylation could reduce the efficiency of transcription elongation [30,31], which would result in a negative correlation between gene body methylation and expression. Furthermore, gene body methylation was reported to be related to the regulation of alternative promoters [32], and may play a role in RNA splicing [33]. Whether these mechanisms co-exist and their relative importance in gene regulation remain not fully explored.

Some of these functional roles of DNA methylation could depend on histone modifications [34]. Strong positive or negative correlations between DNA methylation and various types of histone modifications have been observed at promoters and gene bodies by high-throughput experiments [28,32,35,36].

### The need for quantitative studies

Most of the findings about promoter and gene body methylation described above were based on global trends rather than individual genes. For instance, while promoter methylation has a general negative correlation with gene expression, huge variance exists between both the promoter activities and resulting expression levels of genes with similar methylation levels [19,25,26]. Until now it has been unclear whether it is possible to construct a quantitative model that tells the expression level of an individual gene from its DNA methylation pattern alone or with additional information about histone modifications around its genomic region. Such quantitative modeling would be useful for understanding the combined effect of DNA methylation at different gene sub-elements, such as promoters, exons and introns, on gene expression. It could further help elucidate the relative roles of DNA methylation and other gene regulatory mechanisms in controlling gene expression, and estimate the degree of cooperation and redundancy between them. It could also provide a principal way to identify subsets of genes most affected by DNA methylation in particular cell types.

In recent studies, genomic regions hypo- or hyper-methylated in disease samples have been identified by applying high-throughput methods [37-40]. Having the ability to estimate the effect of DNA methylation on the expression of a gene, quantitative modeling could help identify the most biologically relevant events in disease states, from potentially long lists of differentially methylated regions, for downstream validation and functional studies.

Here, we present our work in quantitatively modeling the relationships between DNA methylation and gene expression using high-throughput sequencing data that cover the methylome and transcriptome of three human samples and additional cell lines at single-base resolution. We show that DNA methylation is highly anti-correlated with gene expression only when the methylation or expression level of a gene is extremely high. We demonstrate that both promoter and gene body methylation are indicative of gene expression level, but gene body methylation has a stronger effect overall. Combining both types of features provides stronger modeling power than considering each type alone. Statistical models constructed from such features can describe the general relationships between DNA methylation and gene expression across different human samples and cell lines. We further demonstrate that DNA methylation could complement histone modification signals in modeling gene expression, and that the quantification measure used for calculating methylation levels has a profound impact on the modeling process and the corresponding biological conclusions.

## Results and discussion

### Data and global patterns

We obtained whole-methylome bisulfite sequencing data at single-base resolution from peripheral blood mononuclear cells (PBMCs) of three individuals in a family trio: Father (F), Mother (M) and Daughter (D) from our previous study (Lee HM et al., Discovery of type 2 diabetes genes using a multiomic analysis in a family trio, submitted). Correspondingly, we extracted total RNA from the three samples and performed whole-transcriptome shotgun sequencing. After data preprocessing, about 95% of the resulting reads were uniquely mapped to the human reference genome (Additional file 1: Table S1).

#### High correlations between methylation patterns in the three genomes

**Figure 1:**
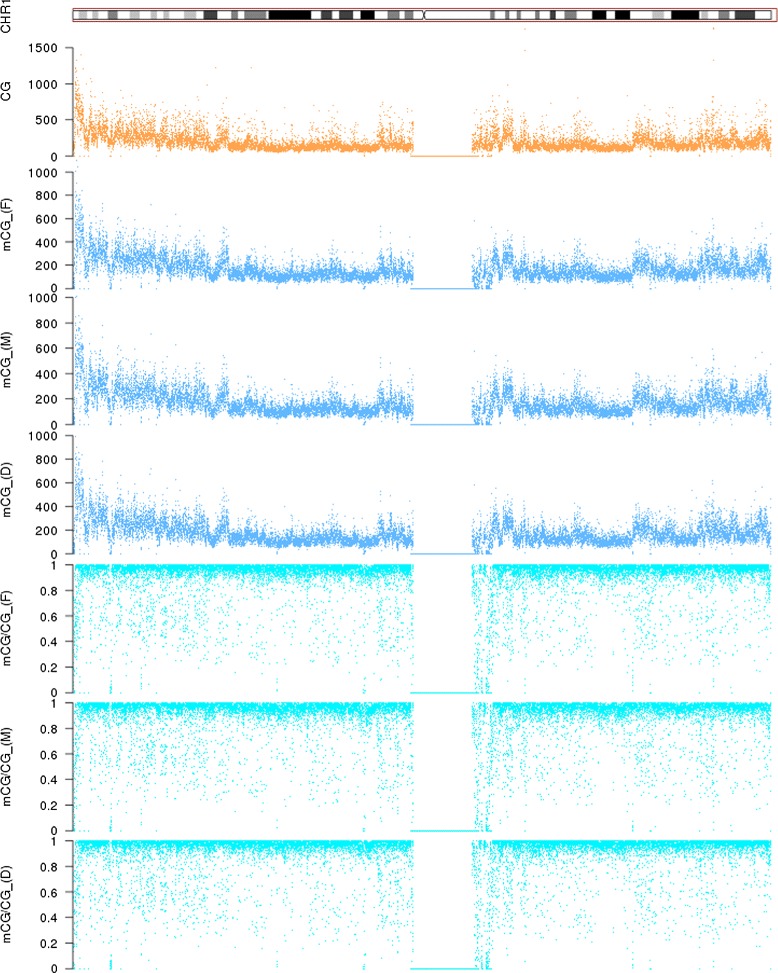
**DNA methylation profiles of the three individuals based on 10 kb sliding windows on chromosome 1.** Abbreviations: CG: number of CpG dinucleotides in each window; mCG: number of methylated cytosines within CpG dinucleotides in each window; (F): Father; (M): Mother;(D): Daughter.

We first explored the global patterns of DNA methylation in the three individuals. Overall, both the absolute number of methylated cytosines within CpG dinucleotides (mCG) in 10 kb sliding windows and the density of methylated cytosines with respect to the total number of CpG dinucleotides within the window (mCG/CG) are highly correlated among the three individuals (Additional file 1: Figure S1 for the whole genome, Figure 1 for chromosome 1 as an example). The methylation density measure mCG/CG has been commonly used in various methylome studies to quantify DNA methylation level [4,22,28]. To check if our data were able to capture subtle DNA methylation differences among the three individuals, we computed the correlation of every 15 adjacent windows between each of the three pairs of individuals. To filter out local fluctuations due to intrinsic randomness in sequencing experiments, we progressively increased the window size from 10 kb to 250 kb. When the window size was 10 kb, both mCG and mCG/CG identified a lot of regions with poor correlation between two individuals (Additional file 1: Figures S2–S7), signifying regions with potential differential methylation status. When the window size was increased, the number of poorly correlated regions decreased for both methylation measures, but the decrease was more rapid for mCG, indicating that mCG/CG is more sensitive to small fluctuations, in particular in windows that contain a small number of CpG dinucleotides.

We collected the low-correlation regions that consistently showed up on the lists at different window sizes, and used DAVID [41] to test for any functional enrichment of the genes inside these regions. At a significance level of p = 0.05 after correcting for multiple hypothesis testing using the Benjamini-Hochberg procedure, some terms were significantly enriched in these genes, including O-methyltransferase (p = 0.0057), melatonin metabolic process (p = 0.023) and hormone biosynthetic process (p = 0.047) (Additional file 1: Table S2). Notably, melatonin secretion was known to be associated with type 2 diabetes (T2D) [42]. As two of the three samples in our study were obtained from individuals with T2D (Lee HM et al., Discovery of type 2 diabetes genes using a multiomic analysis in a family trio, submitted), our results indicated that our data were able to capture relevant information related to the physiological status of the samples.

#### L-shaped patterns between methylated CpG count and gene expression

**Figure 2:**
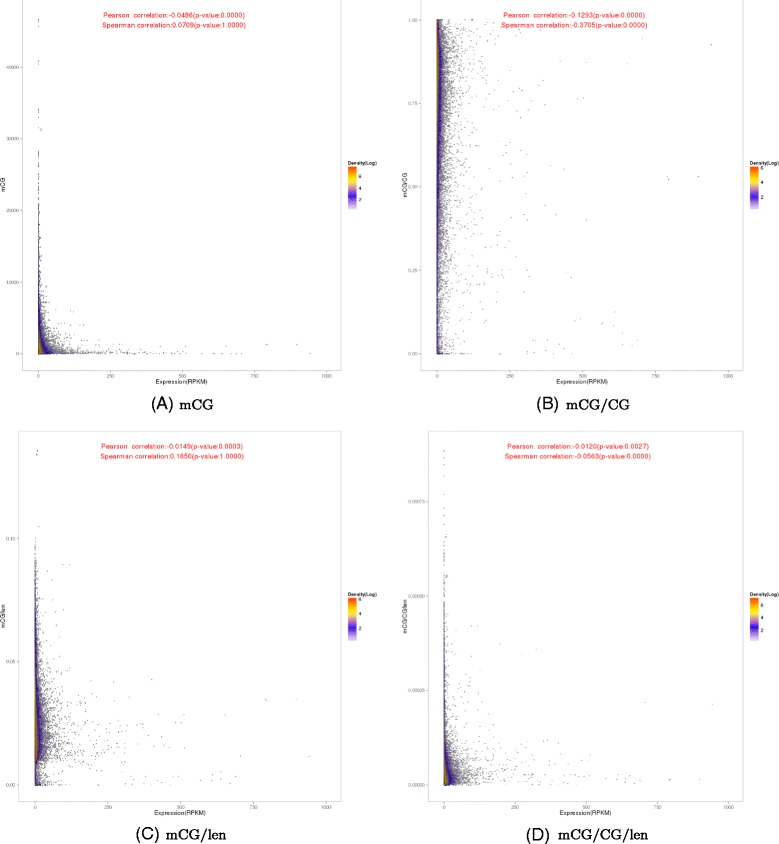
**Relationships between the DNA methylation and expression levels of genes.** Each point in the figure corresponds to a gene. The methylation of a gene is the average level over its body and the 2 kb upstream region. The four panels correspond to the results based on four different DNA methylation measures. Color indicates number of points (in log _2_ scale) within a cell when the occupied space is divided into a 500×500 grid.

We then examined the relationship between DNA methylation and expression levels of genes in the three individuals. We computed the average methylation level along each gene, considering both the gene body and upstream regions, and plotted these methylation levels against the corresponding expression levels. The resulting scatterplot based on the mCG quantification measure of DNA methylation (Figure 2A) displays a very clear “L” shape, in which genes with very high expression levels all display very low methylation levels, and genes with very high methylation levels all show very low expression levels. This pattern suggests that for these extreme cases, there is a negative correlation between DNA methylation and gene expression. However, the majority of genes have both low methylation and expression levels, and the global correlations between DNA methylation and gene expression when all genes are considered were not strong (Figure 2A, Pearson correlation =−0.0486, Spearman correlation = 0.0709), despite significant p-value of the Pearson correlation due to the large number of genesinvolved.

In contrast, the plot based on the normalized measure, mCG/CG, does not display an L-shaped pattern, but rather shows a more global negative correlation with gene expression (Figure 2B, Pearson correlation =−0.1293, Spearman correlation =−0.3705). When the methylation levels were plotted against log expression values instead, the L-shaped patterns became less clear (Additional file 1: Figure S12a,b), but DNA methylation and gene expression were still observed to be weakly anti-correlated.

To get a better understanding on the differences that exist between different quantification measures for DNA methylation, we also normalized mCG by the total length of the measured region (gene body and upstream regions in this case), or by both the number of CpG sites and the region length. We denote these measures as mCG/len and mCG/CG/len, respectively. The two corresponding scatterplots both exhibit some weaker L-shaped patterns (Figure 2C and D).

These observed differences led us to check whether we could find positive correlations between gene body methylation and expression levels as reported in some previous studies [28,29]. To do that, instead of considering both upstream regions and gene bodies at the same time as in Figure 2, we made separate scatterplots for upstream regions (Additional file 1: Figure S8) and gene bodies (Additional file 1: Figure S9). We found a weak positive correlation between gene body methylation and gene expression for the mCG/len measure based on Spearman correlation (Additional file 1: Figure S9c). However, for the other quantification measures, no significant global correlations were observed. For mCG, L-shaped patterns were observed for both upstream regions and gene bodies (Additional file 1: Figures S8a and S9a). We also checked exons (Additional file 1: Figure S10) and introns (Additional file 1: Figure S11) separately, and found no significant differences between the global patterns of these plots and those in which they were taken together as gene bodies. Again, plots based on log expression levels exhibited similar correlation values but less apparent visual patterns (Additional file 1: Figures S12–S16).

These initial results indicate that the relationship between DNA methylation and gene expression is complex and non-linear. The expression levels of genes with very strong methylation levels appear much more affected by DNA methylation than other genes. Whether DNA methylation at promoters and gene bodies have opposite quantitative relationships with gene expression also warrants further investigation.

### Quantitative modeling

To systematically study the quantitative relationships between DNA methylation and gene expression, we performed statistical modeling by means of machine learning. The idea is to compute DNA methylation levels at different sub-regions of a gene as its features, and construct a model that can tell the expression level of any given gene based on its features. The accuracy of a model can be quantified by comparing the model outputs and the actual expression levels of the genes measured by RNA-seq. We constructed different models using different sub-regions and DNA methylation measures, to test which ones could better explain the observed expression levels.

**Figure 3:**
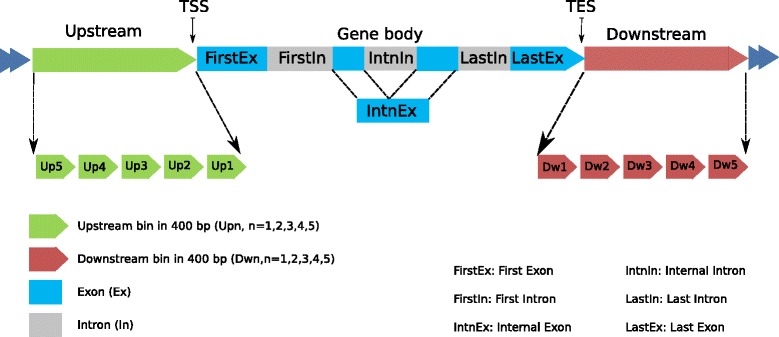
**Sub-regions defined for each gene.** The transcribed region (body) of a gene is divided into 6 variable-length sub-regions according to its exons and introns, namely first exon (FirstEx), first intron (FirstIn), last exon (LastEx), last intron (LastIn), internal exons (IntnEx) and internal introns (IntnIn). The 2 kb upstream region is divided into 5 fixed-length sub-regions Up1-Up5, each of 400 bp. Downstream sub-regions Dw1-Dw5 are defined analogously. In some analyses these sub-regions are further grouped into meta sub-regions, such as Upstream (Up1-Up5), Body (all the exonic and intronic sub-regions) and Downstream (Dw1-Dw5).

Specifically, for each annotated gene, we computed methylation levels in 16 different sub-regions around its gene body and flanking regions (Figure 3). Within the gene body, we defined 6 sub-regions as in a previous study [22], namely first exon (FirstEx), first intron (FirstInt), internal exons (IntnEx), internal introns (IntnInt), last exon (LastEx), and last intron (LastInt). For the upstream and downstream regions, we defined 5 non-overlapping 400 bp sub-regions each (Up1-Up5 and Dw1-Dw5, respectively). We divided all genes into four equal-sized classes according to their expression levels, namely Highest, Medium-high, Medium-low and Lowest, which correspond to genes with expression within the first, second, third and fourth quartiles, respectively. In the first set of models, we combined the data from the three individuals to maximize the amount of data for model construction, resulting in 53,535 (17,845 × 3) data records from 17,845 genes. We tested our models using a left-out procedure, in which two-thirds of the genes from all three individuals were used in model training, and the accuracy of a model was evaluated using the remaining one-third of the genes. We then repeated the procedure 5 times using different random training and testing sets and reported their average accuracy, to ensure the reliability of the results.

#### DNA methylation is partially indicative of expression class

We first constructed models with all DNA methylation features from the 16 sub-regions of each gene, using the mCG methylation measure. We tried 11 different model construction methods, and found that the Random Forest method [43] produced models with the highest cross-validation accuracy, regardless of the exact way model accuracy was computed (Additional file 1: Figure S17). We thus used the modeling accuracy of this method as a proxy of how indicative of gene expression the methylation features are. Based on the AUC measure (area under the receiver operator characteristic curve), the accuracy of the one-class-against-all models for the four expression classes ranged from 0.63 to 0.82 (Additional file 1: Figure S18), where a random assignment of genes to expression classes would result in an AUC value of 0.5, indicating that the methylation features were able to partially separate genes from different expression classes. Among the four expression classes, the Lowest expression class had the highest accuracy, followed by the Highest, Medium-high and Medium-low classes. These results are consistent with what we observed from the scatterplots, that many genes with the lowest expression levels have very high methylation patterns, which can separate them from genes with higher expression levels. The genes with the highest expression levels are slightly more difficult to identify since their signature of low methylation is also shared by many genes from other expression classes. Lacking clear signatures from DNA methylation levels alone, genes in the two medium expression classes are most difficult to identify. The same trends were observed when we repeated the analysis with all four DNA methylation quantification measures and a wide range of expression class numbers (from 2 to 64, Additional file 1: Figures S19–S22).

#### Gene body methylation is a stronger indicator of expression class than promoter methylation

**Figure 4:**
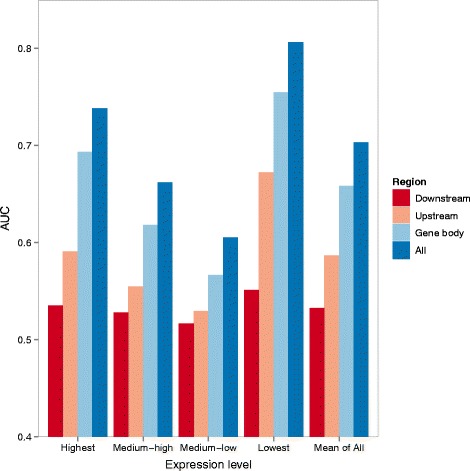
**Accuracy of Random Forest expression models based on DNA methylation features quantified by mCG from three individual sub-regions or their combination.** The accuracy values of genes from the four expression classes are shown in the first four bar groups, while the last bar group shows the average accuracy of the four expression classes.

**Figure 5:**
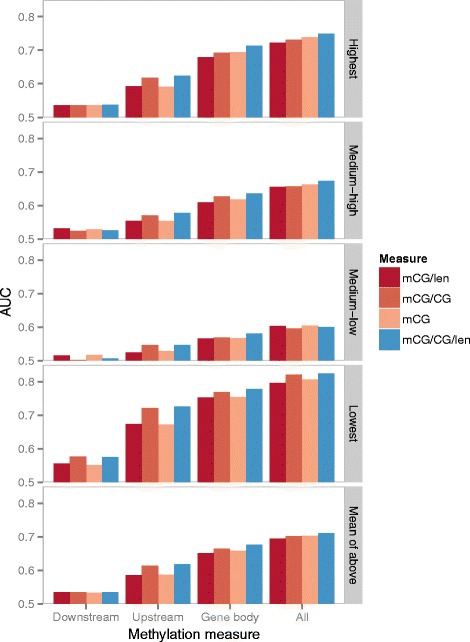
**Comparison of the modeling accuracy based on different DNA methylation measures.** The Random Forest expression models based on the four quantification measures of DNA methylation are shown by different colors. The modeling accuracy involving different subsets of genes from different expression classes are shown in the first four rows, while the last row shows the average accuracy of the four expression classes. Within each row, the four bar groups show the accuracy values of the models constructed from only downstream features, only upstream features, only gene body features, and all of them, respectively. The four quantification measures are ordered according to the average accuracy of their corresponding models when features from all three sub-regions are considered.

We then compared the models constructed using features from either the upstream regions, gene bodies or downstream regions alone (Figure 4). Methylation levels at gene bodies were more capable of telling the expression class of a gene than upstream and downstream regions, for all four expression classes. Combining features from all sub-regions gave the best modeling accuracy, which shows that the features from the different sub-regions are not totally redundant, and may play different roles in gene regulation. These observations stay true for all four methylation quantification measures (Figure 5 and Additional file 1: Figure S23). Comparing the modeling accuracy of the four methylation measures, none of them is clearly better than the others, although on average mCG/CG/len had a slightly higher accuracy.

A potential confounding factor of the above analyses is that the upstream and downstream regions of a transcript could overlap with the body of another transcript [32]. For instance, for a multi-exon gene, if it has a transcript that does not involve the first exon of the gene, DNA methylation at the promoter of the transcript would be counted as gene body methylation of the gene, which may confuse the statistical models. To study how much this would affect the results, we repeated the statistical modeling using the subset of genes with only one annotated transcript isoform. Comparing the resulting models based on different feature sets (Additional file 1: Figure S24), gene bodies still showed stronger modeling power than upstream and downstream regions, and the best accuracy was still obtained by combining features from all three sub-regions.

It was previously shown that DNA methylation of the first exon is linked to transcriptional silencing [44]. We checked whether the higher modeling accuracy of gene body feature was merely due to some strong features extended from the promoter to the first exon. Specifically, we considered two more sub-regions, namely gene bodies excluding the first exons (Genebody–FirstEx) and upstream regions including the first exons (Upstream+FirstEx). We observed that including the first exon in the upstream regions (Upstream+FirstEx) or gene bodies (Genebody) indeed increased the modeling accuracy as compared to having it excluded (Upstream and Genebody–FirstEx, respectively), thus confirming the important role of this sub-region in signifying expression levels (Additional file 1: Figure S25). On the other hand, when we compared upstream and gene body regions, we found that the modeling accuracy of Genebody–FirstEx was higher than Upstream, and that of Genebody was higher than Upstream+FirstEx when all annotated genes were considered (Additional file 1: Figure S25). The same trends were also observed when only genes with one annotated transcript isoform were considered (Additional file 1: Figure S24), except for a slightly higher accuracy of Upstream than Genebody–FirstEx when the mCG/len methylation measure was used. Altogether, our results show that in general, DNA methylation at gene bodies is a stronger indicator of the expression class than DNA methylation at promoters, and it is neither due to overlapping definitions of promoters and gene bodies for multi-transcript genes, nor signals coming from the first exon only.

We also compared the modeling accuracy of exons and introns. For all four quantification measures, methylation levels at exons were consistently a better indicator of expression than methylation levels at introns (Additional file 1: Figure S25), but again the modeling accuracy was higher when both types of features were considered than when either one was used alone.

To test if the above observations are sensitive to the way we define expression classes, we also used a second way to divide genes into four expression classes covering equal range of log-expression values. The results (Additional file 1: Figure S26) show that all the main observations discussed above remain unaffected.

#### Quantitative relationships between promoter and gene body methylation

Since both promoter and gene body methylation are indicative of gene expression to a certain extent, we next explored whether they carry redundant information. When plotting the DNA methylation levels at these two regions for all genes, the distributions based on the four quantification measures were found to be very different (Additional file 1: Figure S27). An L-shaped pattern was observed for mCG (Additional file 1: Figure S27a) and less obviously for mCG/len (Additional file 1: Figure S27c), but not for the other two measures (Additional file 1: Figure S27b and d). Notably, when mCG/CG was used for quantification, the genes were divided into two large clusters (Additional file 1: Figure S27b). Both clusters display very high level of gene body methylation, but one with very high and the other with very low promoter methylation. We also created scatterplots for studying the relationships between the length, the number of CpGs, and the number of methylated CpGs in each sub-region, for each of the 16 types of sub-regions (Additional file 1: Figures S28–S30). The scatterplots between number of CpGs and number of methylated CpGs reveal some interesting patterns about the two clusters in the mCG/CG plot (Additional file 1: Figure S29). For most gene body sub-regions except FirstEx and to some degree LastEx, the genes form a straight line along the diagonal line CG = mCG, showing that the different genes actually have different absolute number of CpGs at their gene bodies, but most of their internal exons and internal introns are fully methylated. In contrast, for the upstream and downstream sub-regions, as well as the first exon, the genes form a tilted V-shaped pattern, with a group of genes lying close to the diagonal CG = mCG and another group lying close to the vertical axis mCG = 0, which correspond to the extreme cases with fully methylated and fully unmethylated CpGs.

To gain more insights into the relationships between promoter and gene body methylation, we included in our analysis the expression levels of the genes (Additional file 1: Figure S31). The three-dimensional scatterplot based on the mCG measure displays the sharpest pattern among the four plots (Additional file 1: Figure S31a), which shows a “triple-inverse” relationship between promoter methylation, gene body methylation and gene expression. This triple-inverse relationship indicates that a gene can either have a high promoter mCG level, a high gene body mCG level, or a high expression level, but not two or three of them simultaneously. This relationship between the three quantities is consistent with the L-shaped patterns we previously observed in the 2D plots (Additional file 1: Figures S8a, S9a and S27a). These results suggest that in terms of the absolute number of methylated CpG sites, either strong promoter methylation or strong gene body methylation alone is sufficient to indicate low expression, and it is not required for a gene to redundantly have both indicators.

#### Potential role of gene body methylation for genes with CpG-poor promoters

**Figure 6:**
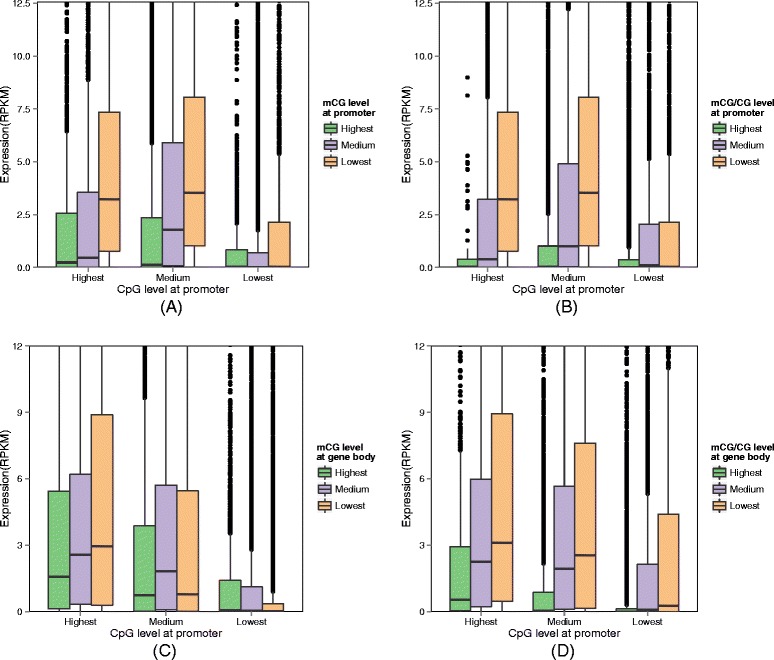
**Relationship between DNA methylation and gene expression for genes with different promoter CpG levels.** The four panels show the expression levels of different subsets of genes and their corresponding methylation levels at upstream **(A and B)** or transcribed regions **(C and D)**. Panels **A** and **C** involve the use of the mCG methylation measure, while panels **B** and **D** involve the use of the mCG/CG measure. Within each panel, the genes are first divided into three subsets according to their promoter CpG levels, which correspond to three bar groups. For each subset, the genes are further divided into another level of three subsets based on their methylation level. Finally, for each of the resulting subset of genes, their distribution of expression levels is shown by a Box and Whisker plot.

It has been proposed that for CpG island promoters, DNA methylation is a sufficient but not necessary condition for gene inactivation, while for CpG-poor promoters, DNA methylation does not preclude expression [19]. To check whether the same observations could be made in our data, we plotted the expression level of different groups of genes according to their promoter CpG levels (Figure 6A and B). Indeed, the expression levels of genes with a large number of CpG dinucleotides in their promoter regions were more strongly affected by the DNA methylation in these regions. Specifically, for both mCG and mCG/CG measures, promoter methylation was more anti-correlated with gene expression for genes with highest or medium promoter CpG levels (first two bar sets of the figures) than those with lowest promoter CpG levels (last bar sets of the figures). Genes with lowest promoter CpG levels were largely insensitive to promoter methylation, and had low expression levels in general.

For this group of genes with CpG-poor promoters, can gene body methylation indicate their expression levels? To answer this question, we again divided genes into three groups according to their promoter CpG counts, but this time we studied the correlation between gene body methylation and expression levels of each group instead (Figure 6C and D). For both mCG and mCG/CG, the genes with CpG-poor promoters do exhibit some weak differential expression patterns as gene body methylation level varies, but the correlation between gene body methylation and expression was positive for mCG and negative for mCG/CG. These results suggest a potential role of gene body methylation in regulating genes with CpG-poor promoters, although the exact mode of regulation is yet to be understood.

### Generality of the quantitative models

All the results above were based on quantitative models both constructed and tested on the same individuals (albeit on different subsets of genes), using data from one single cell type (PBMC). To test if these models are generally useful for signifying expression classes, we collected single-base resolution bisulfite sequencing and RNA-seq data for two cell lines, H1 human embryonic stem cells (hESC) and the human lung fibroblast line IMR90, from the Roadmap Epigenomics Project [45] (Additional file 1: Table S3). We constructed models using DNA methylation and expression data from one individual/cell line, and applied the models to predict the expression class of genes in another individual/cell line based on its DNA methylation profile alone. To ensure the generality of the models, the genes used for training in the first individual/cell line and the genes used for testing in the second individual/cell line were mutually exclusive.

**Figure 7:**
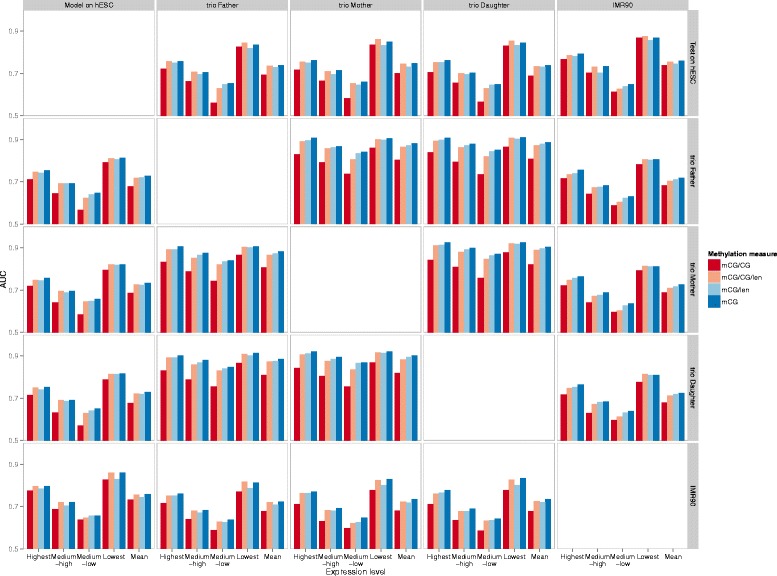
**Generality of the quantitative models.** Random Forest expression models were constructed using methylation and expression data from one of the individuals or cell lines, indicated by the different columns. The methylation level of a gene is defined as the average level over its upstream, transcribed and downstream regions. These models were used to predict the expression levels of genes in another individual/cell line, based on their measured DNA methylation levels of them in it. For each of these model training/testing combinations, the prediction accuracy values of the genes in different expression classes, and their overall average, are shown in different bar groups. Within each bar group, the accuracy values based on the four DNA methylation measures are shown.

The results (Figure 7) show that, for all combinations of training and testing individuals/cell lines, the prediction accuracy was much higher than random predictions (which would have an AUC value of 0.5). Models constructed from any one of the three individuals were able to predict the expression classes of genes in another individual with an average AUC of about 0.9, which is expected as these samples all contained PBMC from individuals in the same family. More interestingly, the other data set combinations also have prediction accuracy of about 0.75 on average, which demonstrate the generality of the constructed models. These cross-sample results reconfirm our earlier findings that the more extreme expression classes are better indicated by methylation patterns. Moreover, among the four methylation quantification measures used, mCG, mCG/len and mCG/CG/len consistently provided better modeling accuracy than mCG/CG (Figure 7), which indicates that the commonly-used quantification measure of DNA methylation, mCG/CG, is not necessarily the best in signifying gene expression classes.

### Quantitative relationship with histone modifications

Our quantitative models based on DNA methylation were able to achieve reasonable accuracy in identifying the expression class of a gene, but they also show that DNA methylation alone is not informative enough to signify precise expression levels. We have previously shown that histone modifications are strong indicators of expression levels [46,47]. Therefore, we next explored the relationship between DNA methylation and histone modifications in terms of indicating gene expression, and tested whether information on gene expression conveyed by DNA methylation is totally subsumed by that of histone modifications. It was previously shown that promoter methylation was negatively correlated with H3K4me3 (histone 3 lysine 4 trimethylation) in the human brain [32], and gene body methylation was positively correlated with H3K36me3 and negatively correlated with H3K27me3 in a B-lymphocyte cell line [28]. To study the quantitative relationships between DNA methylation and histone modifications in the context of indicating expression levels, we compared statistical models that involve either only DNA methylation features, only histone modification features, or both.

We collected ChIP-seq data for 26 types of histone modification from the H1 embryonic cell line from the Roadmap Epigenomics Project (Additional file 1: Table S3). As with DNA methylation, we computed the average signal of each type of histone modification in the same 16 sub-regions for each gene. Although some histone marks are known to be enriched in particular sub-regions, this knowledge is limited to some well-studied types of histone modifications. We therefore considered all sub-regions and let the Random Forest method identify the features most useful for indicating expression levels.

**Figure 8:**
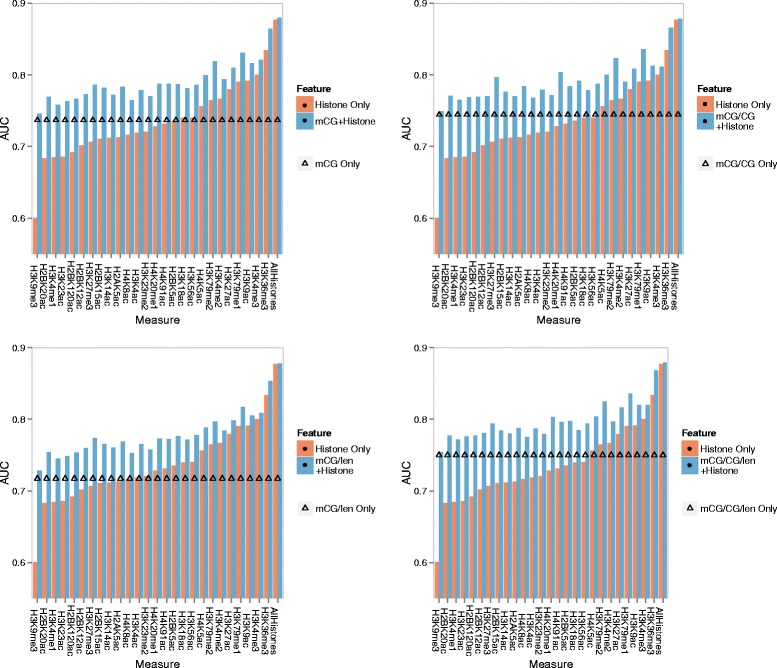
**Joint effects of DNA methylation and histone modifications on gene expression.** The four panels compare Random Forest expression models with only DNA methylation features (straight line with triangle markers), only histone modification features (orange bars), or both (blue bars). The four panels involve DNA methylation levels computed by different quantification measures. For DNA methylation and any type of histone modifications, its signal level is computed as the average over the upstream, transcribed and downstream regions of a gene. In each panel, the first 26 bar groups correspond to models involving one of the 26 types of histone modification, while the last bar group corresponds to the model involving all 26 types of histone modification.

As expected, some of the models constructed from histone modification features alone had high cross-validation accuracy (Figure 8). Consistent with previous findings, the two strongest feature sets were H3K36me3 and H3K4me3, which mark actively transcribed regions and active promoters, respectively [48]. Models based on DNA methylation features alone were not as accurate as those constructed from these histone modification features well-known for their roles in marking gene activities, but were more accurate than many other types of histone modification such as H3K9me3 and H3K4me1 (Figure 8).

#### DNA methylation and histone modifications contain non-redundant information about gene expression

Interestingly, regardless of the type of histone modification and the DNA methylation measure used, combining both types of features consistently increased the accuracy of the corresponding models involving only histone modification features or only DNA methylation features. Even for the strongest histone modification feature set derived from H3K36me3, incorporating DNA methylation features still led to an improvement of modeling accuracy by about 6%, from AUC value of 0.83 to 0.88 for mCG/CG/len, which indicates that the two types of signals were not completely redundant in terms of signifying gene expression.

**Figure 9:**
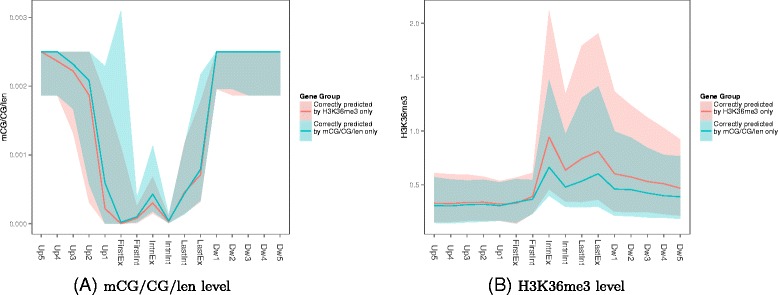
**DNA methylation and H3K36me3 levels of genes the expression classes of which were correctly identified by either the mCG/CG/len model but not the H3K36me3 model, or vice versa.** In the figures, the solid lines represent the median signal value of all genes in the group, and the shaded area of the same color tone marks the 25-th precentile to 75-th percentile range.

To better understand how DNA methylation complements histone modification in indicating expression classes, we examined the DNA methylation and H3K36me3 signal levels of two types of genes, namely (1) those with expression classes correctly identified by the model involving only mCG/CG/len features but not by the model involving only H3K36me3 features, and (2) the vice versa, i.e., those with expression classes correctly identified by the H3K36me3 model but not the mCG/CG/len model. The genes with expression classes correctly identified by the mCG/CG/len model only displayed higher mCG/CG/len levels (Figure 9A, blue lines and areas) and lower H3K36me3 levels (Figure 9B), indicating that in general they were the less transcribed genes. Among the different sub-regions, as expected the ones best separating the two groups of genes in terms of H3K36me3 signals were those within the gene bodies, and to a lesser extent those at downstream regions (Figure 9B). Interestingly, in terms of mCG/CG/len levels, the sub-regions that best separate the two groups of genes were the exonic regions, especially the first exon (Figure 9A), indicating that methylation levels at exonic regions not only play crucial roles in models involving DNA methylation features alone, but could also be important in complementing histone modifications in indicating the expression class of a gene.

As in the case of DNA methylation, histone modification features were most successful in identifying genes with lowest expression levels (Additional file 1: Figure S32). However, even the strongest histone modification features were not significantly better than DNA methylation in identifying these genes. In contrast, some of them were much better in identifying genes with medium expression levels, suggesting that DNA methylation mainly indicates the coarse on/off status of a gene, while some histone marks provide more fine-grained details about the precise expression levels.

**Figure 10:**
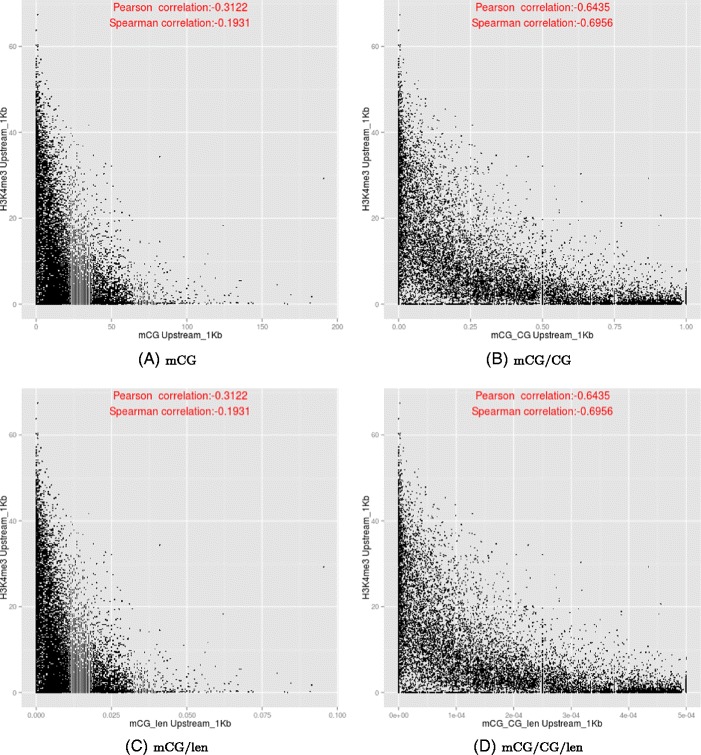
**Relationships between the DNA methylation (y-axis) and H3K4me3 (x-axis) at the upstream regions of genes, based on the four DNA methylation measures.**

**Figure 11:**
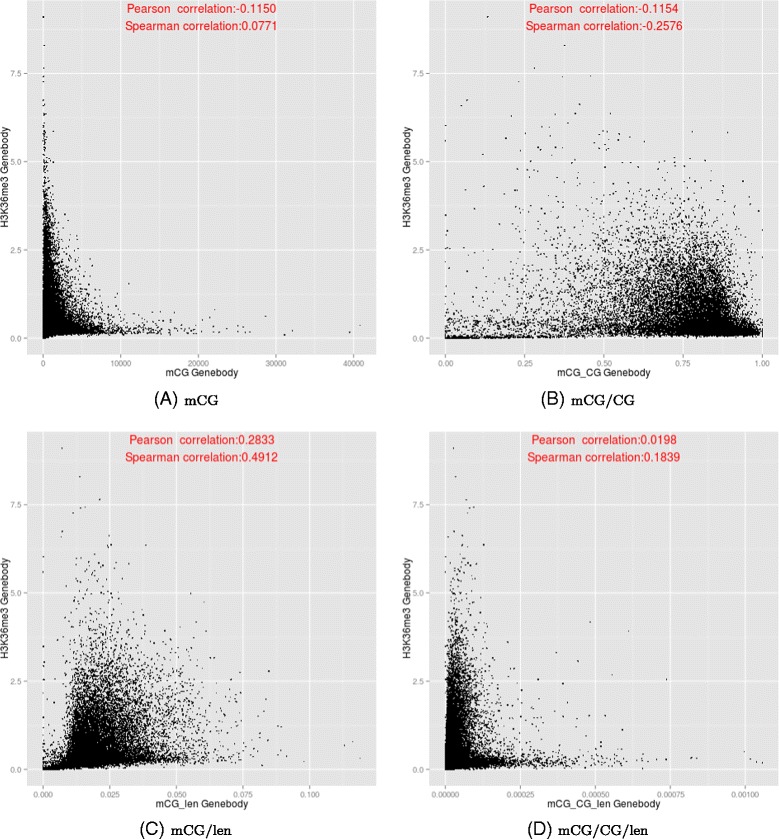
**Relationships between the DNA methylation (y-axis) and H3K36me3 (x-axis) at the transcribed regions of genes, based on the four DNA methylation measures.**

We examined the relationships between DNA methylation and histone modifications in more detail by plotting their values in different sub-regions of genes (Additional file 1: Figures S33–S34). In particular, we reconfirmed previous findings that DNA methylation and H3K4me3 negatively correlate at the upstream region (Figure 10). However, whether gene body methylation positively or negatively correlates with H3K36me3 depends on the DNA quantification measure (Figure 11), with the correlation being most positive for mCG/len, and most negative for mCG/CG.

#### A small number of DNA methylation and histone modification features are sufficient to maximally indicate gene expression

When we combined features derived from DNA methylation and all 26 types of histone modifications, the resulting model had a higher accuracy than all the models involving single histone modification and/or DNA methylation features (Figure 8). To test if it is possible to achieve the same accuracy with a smaller number of feature sets, we applied a forward feature selection procedure. Specifically, we started with either an empty set of features, or all DNA methylation features based on one quantification measure. We then iteratively added the set of features for the type of histone modification that could maximize the accuracy gain, until no more sets could lead to any further improvements. Depending on the DNA methylation features included in the first step, maximal accuracy was achieved by 6-8 feature sets in total (Additional file 1: Figure S45).

Consistent with the single-feature-set results, H3K36me3 and H3K4me3 were always the features first incorporated into the models. The features next incorporated include those that involve H3K79, and the repressive mark H3K27me3. For the DNA methylation measures mCG, mCG/CG and mCG/CG/len, including DNA methylation features resulted in final models with higher accuracy than the one involving histone modification features alone, indicating that DNA methylation has non-negligible roles in these models with maximal modeling accuracy.

Since the AUC values were increased most by H3K36me3 and H3K4me3, and these two marks are well-known to be most indicative of expression levels, we believe similar results would be obtained if we had applied other feature selection methods.

## Conclusions

Previous studies have examined high-level qualitative relationships between DNA methylation and gene expression. In this work, we have demonstrated that DNA methylation status alone can indicate the expression class of a gene with fairly high accuracy. The generality of our models has been confirmed by their cross-sample/cell line modeling capability. Our models provide a means to analyze the detailed quantitative relationships between DNA methylation and expression, with systematic assessments of the level of expression variations explainable by DNA methylation.

We showed that two groups of genes have particularly clear methylation profiles in our data, namely genes that lie on both ends of the spectrum – those with very high methylation and very low expression levels, and those with very high expression and very low methylation levels. If we apply a simple classification of genes into those with high or low expression and DNA methylation levels, among the four possible combinations, the one with both high expression and high DNA methylation is almost devoid of genes when three out of the four DNA methylation quantification measures were used. The resulting scatterplots exhibit clear L-shaped patterns (Figure 2), which correspond to an exclusive OR (XOR) relationship between DNA methylation and gene expression. Our results indicate that on the one hand, strong DNA methylation is sufficient to indicate low expression of a gene, but on the other hand, while low DNA methylation is permissive of transcription, the actual expression level of a gene is largely determined by other factors.

We further demonstrated that one class of such factors is histone modification. Some types of histone modification, including H3K4me3 and H3K36me3, are much stronger indicators of precise expression levels of individual genes than DNA methylation. However, we found that incorporating DNA methylation features consistently improved the modeling power of the models involving either of these histone marks alone, or even the one involving all types of histone modification combined (Figure 8). Notably, we found that DNA methylation levels at exonic regions helped determine the expression class of some genes in our models when H3K36me3 features failed to do so.

A key finding of this study is that gene body methylation is a stronger indicator of expression class than promoter methylation for genes in all expression classes. Our results are consistent with the strong effects of gene body methylation on expression previously observed in plants [49,50]. We provided evidence that the stronger modeling power of gene body methylation could not be explained by the effects of first exons alone or biases caused by the presence of multiple transcript isoforms in a single gene, nor was it affected by the quantification measure of DNA methylation levels. We also found that combining both promoter and gene body DNA methylation features resulted in a better modeling accuracy of gene expression classes. The “triple-inverse” pattern observed between promoter methylation, gene body methylation and gene expression (Additional file 1: Figure S31a) suggests that promoter and gene body methylation exert repressive effects on different sets of genes. Previous studies have proposed that promoter methylation is linked to blockage of transcription factors, while gene body methylation is related to the recruitment of transcriptional repressors and reduction of transcriptional elongation [1,9,31]. The potentially divergent roles of DNA methylation at the two types of regions are consistent with the higher modeling accuracy achieved in our study when both types of features were considered. Since the on/off role of promoter methylation appears to affect a relatively small set of genes with extreme methylation levels, we speculate that the effect of gene body methylation on reducing transcription efficiency may be a more general mechanism that affects a broader group of genes, which provides a plausible explanation for the stronger modeling power of the gene body methylation features in our current study.

We propose that the different functions of DNA methylation in transcriptional regulation are better reflected by multiple quantification measures rather than one single measure. It is possible that the raw number of methylated CpG sites, mCG, is a proxy of the total time of an elongating polymerase being slowed down by gene body methylation. Another quantification measure, the number of methylated CpG sites per unit length, mCG/len, may be more related to the average speed reduction of the elongating polymerase. Finally, the commonly-used density measure mCG/CG represents a comparison between methylated and unmethylated CpG sites in a given genomic region, which may reflect the “competitiveness” of the region for events such as protein binding. In this study, we demonstrated that these quantification measures used to represent methylation levels at a given genomic region could exhibit drastically different patterns when analyzed together with gene expression and histone modification signals. However, all of them were able to model expression classes reasonably well and none of them was clearly better than all the others. Further investigations are needed to study the detailed mechanistic meanings of these different quantification measures.

Our results offer several possible explanations for the apparent discrepancies among previous studies examining the relationships between gene body methylation and gene expression, that in some studies they were observed to be positively correlated [31,32] and in others, negatively correlated [28,29,51,52]. We found that substantially different correlation values could be obtained by using different quantification measures of DNA methylation, and different ways to compute the correlations. For example, whereas rank-based Spearman correlation is more strongly affected by the bulk of genes with low methylation and expression levels as they occupy a wide range of rank values, value-based Pearson correlation is more influenced by genes with more extreme methylation and expression levels. Calculating correlations using different subsets of genes, such as all genes versus only those with observable expression values, could also lead to very different conclusions. The discrepancies in the previous studies could be due to these and other subtle data processing and analysis details.

Further studies will be needed to elucidate how promoter and gene body methylation of different transcripts of a gene are coordinated. Signals that cover a broad area, such as DNA methylation over whole transcript bodies, have a high chance of interfering with other transcripts. The coordination would be simplest if promoter and gene body methylation both take on a repressive role, and different transcript isoforms of a gene co-express in a synchronized manner. In that case, DNA methylation would be mainly responsible for marking genes with all transcripts repressed. The co-expression of transcript isoforms was indeed observed in large-scale sequencing data from human cells [53], although it is still unclear whether the different isoforms expressed simultaneously in the same cell, or actually different subsets of them were expressed in different sub-populations of the cells from which RNA was extracted and sequenced. Alternatively, intragenic DNA methylation that intersects promoters of some transcripts may be involved in regulating the use of alternative promoters [32]. Whether other, more complex types of coordination exist is yet to be studied.

Our study of the quantification measures at different genic sub-regions was facilitated by whole-genome bisulfite sequencing data at single-base resolution. Some other experimental methods produce data at a lower resolution (such as ChIP-based or affinity-capture-based methods), have incomplete genome coverage, especially at transcribed regions (such as some array-based methods), or provide information for only some types of DNA methylation quantification. Nevertheless, whole-genome bisulfite sequencing has a relatively high cost, and it requires extensive computations in data processing. For practical purposes, it would be crucial to choose a suitable experimental method based on the goal of the study. For example, methylation profiles are obtained from case and control samples in some disease studies, to identify differentially methylated regions with functional consequences. Our results offer new insights into choosing the suitable experimental method by indicating that for the vast majority of genes with moderate or low methylation levels, their expression levels are only weakly reflected by methylation levels, but more strongly affected by other factors. Therefore, if one is to make hypotheses based on the methylation data alone, it is more reasonable to consider only genes with extreme methylation levels. These extreme cases can probably be identified using low-resolution data with partial genome coverage. In contrast, if one wants to identify all differentially methylated genes for downstream experimental testing of their functional effects, data with higher resolution can probably provide more details about subtle differences that exist among the various samples. Additionally, it has recently been proposed that methylation at distal enhancer sites may cause differential gene expression in disease samples [54], the study of such phenomena would better be conducted using data with whole-genome coverage.

## Materials and methods

### Sample collection

We collected DNA methylation and gene expression data from a family trio from our previous study (Lee HM et al., Discovery of type 2 diabetes genes using a multiomic analysis in a family trio, submitted). In the following, we briefly descirbe the sample collection, data generation and data processing proceses.

Blood samples were obtained from a Chinese family trio consisting of a father, a mother and a daughter, which we denote as F, M and D, respectively. Peripheral blood mononuclear cells (PBMCs) were isolated using Ficoll-Paque stepwise gradient centrifugation. The isolated PBMCs were divided for DNA and RNA extraction. Total DNA was prepared using proteinase K digestion and phenol extraction. Total RNA was extracted by Trizol (Life Technologies, Carlsbad, CA, USA) following the manufacturer’s protocol. The quality of the RNA samples was checked by Bioanalyzer (Agilent Technologies, Palo Alto, CA, USA) before they were subjected to sequencing.

### Methylome sequencing and data processing

Bisulfite sequencing and data processing were carried out as described previously [22]. DNA was fragmented by sonication to 100 to 500 bp in size, followed by end-blunting, dA addition at the 3’end and ligation of adapters. The adapter sequence contained multiple methylcytosines to allow monitoring of the efficiency of the bisulfite conversion. Unmethylated cytosines were converted to uracils by bisulfite treatment using a modified protocol from Hayatsu [55]. DNA fragments in the size range of 320 to 380 bp were gel-purified for sequencing. All procedures were performed according to the manufacturer’s instructions. Converted DNA was subjected to 50 bp paired-end sequencing using an Illumina Solexa GA sequencer (Illumina, San Diego, CA, USA). All the raw data were processed by the Illumina Pipeline v1.3.1 (Illumina, San Diego, CA, USA).

The cleaned reads generated were aligned to the reference human genome hg18 as follows. Since DNA methylation is strand-specific, the two strands of the reference human genome were modified separately *in silico* to convert all C’s to T’s, to generate a combined 6 Gbp target genome for aligning reads after bisulfite conversion. Correspondingly, the sequencing reads were also transformed using the following criteria: (1) observed C’s in the forward reads were replaced by T’s; and (2) observed G’s in the reverse reads were converted to A’s. The transformed reads were then aligned to the modified target genome using the SOAP2 aligner [56]. All the reads mapped to unique locations with minimum mismatches and clear strand information were defined as uniquely matched reads, and were used to determine the methylated Cytosines. According to the alignment results, the unconverted C’s and G’s from the original read sequences before the transformation were used to identify the methylated Cytosines. Bases with low quality scores were filtered to ensure accuracy of the results. The methylated Cytosines were defined as those having a significant number of reads supporting its methylated status, with less than 1% FDR according to a binomial distribution, as suggested previously [22]. All the Cytosine positions were then lifted over to the reference human genome hg19 by the LiftOver utility provided by the UCSC Genome Browser [57] for downstream analyses.

### Transcriptome sequencing and data processing

Total RNA extracted from each sample was enriched by oligo-dT to get the polyA+ fraction for sequencing. The polyA+ mRNAs were then fragmented and converted to cDNA by reverse transcription. After ligation of the 5’ and 3’ sequencing adaptors to the cDNA, DNA fragments were size-selected for 75 bp paired-end sequencing by Illumina Genome Analyzer II using standard procedures. All the raw data were processed by the Illumina Pipeline v1.3.1. All sequencing reads were trimmed dynamically according to the algorithm provided by the -q option of the BWA tool [58]. After trimming, read pairs with both sides having at least 35 bp were retained and mapped to the human reference genome hg19 using TopHat [59] (v.1.1.4) with the following parameters: microexon-search, butterfly-search and -r 20. The expression value of a gene was computed by the RPKM (reads per kilobase of exons per million mapped reads) measure [60], defined as the number of reads that cover it (in million reads) normalized by its length (in kilobase) and the total number of reads in the data set.

### Definition of the four DNA methylation quantification measures

We defined four methylation measures based on methylated CpG sites. The first measure is the absolute number of methylated CpG sites in a region, denoted as mCG. The second measure is the density of methylated CpG sites relative to the total number of CpG sites in a region, denoted as mCG/CG. The third measure is the density of methylated CpG sites relative to the length of a region, denoted as mCG/len. The fourth measure is the number of methylated CpG sites normalized by both the total number of CpG sites and the length of a region, denoted as mCG/CG/len.

### Visualizing global DNA methylation patterns and computing local correlations between two individuals

We constructed global DNA methylation profiles of the three individuals as follows. We first divided up the human genome into 10 kb windows. In each window, we computed the DNA methylation level based on one of the four quantification measures. We then visualized the resulting global patterns using IGV [61] and Circos [62]. To compute local correlations of DNA methylation profiles between two individuals, we divided up the genome into fixed-length windows (of size 10 kb, 50 kb, 100 kb or 250 kb), and computed the DNA methylation level in each window. For every 15 consecutive windows, we then computed the Pearson correlation between two individuals (F vs. M, F vs. D or M vs. D). The resulting distributions of correlation values were visualized using Box and Whisker plots.

### Enrichment analysis of regions with low methylation correlations

We collected regions with methylation correlations less than 0.5 between any two of the three individuals based on the mCG quantification measure. We found that most of the regions obtained from the analysis based on 100 Kb window size consistently showed up on the list at different window sizes, and thus we focused on this list of regions. We extracted the genes within these regions and submitted it to the DAVID tool [41] for enrichment analysis with default parameters. The p-values reported were corrected by the Benjamini-Hochberg procedure [63].

### Definition of gene sub-regions

For analyses involving genes, we considered the level 1 and level 2 protein-coding genes annotated in Gencode v7 [64], based on composite gene models. We defined the body of a gene as the first transcription start site of its annotated transcripts to the last transcription termination site of its annotated transcripts. Within the gene body, we defined any region annotated as an exon in any of the associated transcripts as an exon of the gene. We then defined sub-regions of a gene as shown in Figure 3 and explained in the Results and discussion section. We discarded genes with less than 4 exonic regions after merging overlapping exons from different transcripts, resulting in a set of 17,845 genes.

### Definition of expression classes

By default we defined gene expression classes as follows. We first combined the genes from the three individuals into a set of 53,535 (17,845 ×3) genes. Each of them was then assigned to one of four expression classes, namely the “Highest”, “Medium-high”, “Medium-low” and “Lowest” classes, which contained genes with expression levels within the first, second, third and fourth quartiles on the list of expression values sorted in descending order. The Lowest expression class could contain genes with zero RPKM values. In some analyses, we also defined other numbers (2, 8, 16, 32 or 64) of expression classes in similar ways.

We also tested a second way of defining expression classes, in which classes A, B, C and D contained genes with expression level within (log min+3*x*, log max], (log min+2*x*, log min+3*x*], (log min+*x*, log min+2*x*] and [ log min, log min+*x*], respectively, where min and max are the minimum and maximum expression values among all genes, respectively, and $x = \frac {(\log \max - \log \min)}{4}$.

### Statistical modeling

We used 11 different methods to construct statistical models, including 5-Nearest Neighbors, 10-Nearest Neighbors, 20-Nearest Neighbors, Naive Bayes, Bayesian Network, Decision Trees (C4.5), Random Forests, Logistic Regression, Support Vector Machine (SVM) with linear kernel, SVM with second-degree polynomial kernel, and SVM with Radial Basis Function (RBF) kernel. We used the implementation of all these methods in Weka [65]. We constructed statistical models using these methods with features derived from DNA methylation and/or histone modification levels of the different genic sub-regions. We first constructed models for the three individuals using their combined data. We randomly sampled 1/3 of the genes as a left-out testing set. The remaining 2/3 of the genes were used to perform model training. The constructed model was then applied to the left-out set to compute the accuracy. For each setting, we repeated the process five times to compute an average accuracy of the five models.

We also tested the generality of our models by constructing models using the DNA methylation and gene expression data of a random set of 2/3 of the genes from one single individual/cell line for training, and applying the model to predict the expression levels of the remaining 1/3 of the genes in another individual/cell line based on the DNA methylation levels in this individual/cell line.

### Collection and processing of cell line data

We downloaded data for human embryonic stem cells and human lung fibroblast line IMR90 produced by Roadmap Epigenomics [45] from the Gene Expression Omnibus (GEO) [66] web site. The RPKM measure was used to compute the level of histone modification in each given region. For data sets with replicates, we used the mean values of the replicates for computing the histone modification signals.

### Data availability

All raw sequencing reads have been deposited into NCBI Sequence Read Archive under entry SRP033491. All the processed data files used in this study can be found at http://yiplab.cse.cuhk.edu.hk/means/.

## Additional file

Additional file 1
**Supplementary Information.**
