# Supplementary material for: Whole-genome bisulfite sequencing of multiple individuals reveals complementary roles of promoter and gene body methylation in transcriptional regulation
Source: Genome Biol. 2014 Jul 30;15(7):408. doi: 10.1186/s13059-014-0408-0 (PMC4189148; doi:10.1186/s13059-014-0408-0)
Supplement: Additional file 1 — Supplementary Information. [file 13059_2014_408_MOESM1_ESM.pdf]

Supplementary Information

Supplementary tables

Table S1: Summary statistics of the bisulfite sequencing and RNA sequencing data from the three samples of the family trio.

Individual	Father	Mother	Daughter
Bisulfite sequencing			
Number of reads (million reads)	3,069	2,821	2,646
Number of bases (Gb)	152	140	141
Number of mapped bases (Gb)	105	104	108
Mapping ratio (%)	69.3	74.5	76.8
Average coverage	35x	35x	36x
RNA sequencing			
Number of reads (million reads)	82.0	121.5	154.8
Number of bases (Gb)	5.3	8.3	10.4
Number of mapped bases (Gb)	78.9	118.1	150.1
Mapping ratio (%)	96.2	97.2	96.9

Table S2: Terms enriched in the genes within regions with low methylation correlation between the three individuals based on the mCG quantification measure.

Category	Term	Benjamini-Hochberg-corrected p-value
INTERPRO	IPR001077:O-methyltransferase, family 2	0.0057
GOTERM_BP_FAT	GO:0030186 melatonin metabolic process	0.023
GOTERM_BP_FAT	GO:0030187 melatonin biosynthetic process	0.023
GOTERM_MF_FAT	GO:0017096 acetylserotonin O-methyltransferase activity	0.011
GOTERM_BP_FAT	GO:0046219 indolalkylamine biosynthetic process	0.019
GOTERM_BP_FAT	GO:0042435 indole derivative biosynthetic process	0.019
GOTERM_BP_FAT	GO:0042434 indole derivative metabolic process	0.036
GOTERM_BP_FAT	GO:0006586 indolalkylamine metabolic process	0.036
GOTERM_BP_FAT	GO:0042430 indole and derivative metabolic process	0.036
GOTERM_MF_FAT	GO:0008171 O-methyltransferase activity	0.026
GOTERM_BP_FAT	GO:0042401 biogenic amine biosynthetic process	0.057
GOTERM_BP_FAT	GO:0042446 hormone biosynthetic process	0.047

Table S3: List of DNA methylation (bisulfite sequencing), gene expression (RNA-seq) and histone modification (ChIP-seq) data sets from the H1 human embryonic stem cells (hESC) and IMR90 human lung fibroblast line used in this study.

Cell	Data type	GEO ID	Cell	Data type	GEO ID
hESC	Bisulfite sequencing	GSM429321	hESC	ChIP-seq (H3K4me1)	GSM466739
hESC	Bisulfite sequencing	GSM429322	hESC	ChIP-seq (H3K4me1)	GSM605312
hESC	Bisulfite sequencing	GSM429323	hESC	ChIP-seq (H3K4me2)	GSM602260
hESC	Bisulfite sequencing	GSM432685	hESC	ChIP-seq (H3K4me2)	GSM602261
hESC	Bisulfite sequencing	GSM432686	hESC	ChIP-seq (H3K4me3)	GSM409308
hESC	ChIP-seq (H2AK5ac)	GSM602257	hESC	ChIP-seq (H3K4me3)	GSM469971
hESC	ChIP-seq (H2AK5ac)	GSM602258	hESC	ChIP-seq (H3K4me3)	GSM605315
hESC	ChIP-seq (H2BK120ac)	GSM605295	hESC	ChIP-seq (H3K56ac)	GSM605317
hESC	ChIP-seq (H2BK120ac)	GSM789280	hESC	ChIP-seq (H3K56ac)	GSM667627
hESC	ChIP-seq (H2BK120ac)	GSM789281	hESC	ChIP-seq (H3K79me1)	GSM605318
hESC	ChIP-seq (H2BK12ac)	GSM605296	hESC	ChIP-seq (H3K79me1)	GSM605319
hESC	ChIP-seq (H2BK12ac)	GSM605297	hESC	ChIP-seq (H3K79me1)	GSM605320
hESC	ChIP-seq (H2BK15ac)	GSM605298	hESC	ChIP-seq (H3K79me2)	GSM605321
hESC	ChIP-seq (H2BK15ac)	GSM605299	hESC	ChIP-seq (H3K79me2)	GSM605322
hESC	ChIP-seq (H2BK20ac)	GSM605300	hESC	ChIP-seq (H3K9ac)	GSM434785
hESC	ChIP-seq (H2BK20ac)	GSM605301	hESC	ChIP-seq (H3K9ac)	GSM605323
hESC	ChIP-seq (H2BK5ac)	GSM605302	hESC	ChIP-seq (H3K9me3)	GSM605325
hESC	ChIP-seq (H2BK5ac)	GSM605303	hESC	ChIP-seq (H3K9me3)	GSM605327
hESC	ChIP-seq (H3K14ac)	GSM667614	hESC	ChIP-seq (H3K9me3)	GSM605328
hESC	ChIP-seq (H3K14ac)	GSM667615	hESC	ChIP-seq (H3K9me3)	GSM818057
hESC	ChIP-seq (H3K18ac)	GSM602259	hESC	ChIP-seq (H4K20me1)	GSM605329
hESC	ChIP-seq (H3K18ac)	GSM605304	hESC	ChIP-seq (H4K20me1)	GSM789284
hESC	ChIP-seq (H3K23ac)	GSM667617	hESC	ChIP-seq (H4K5ac)	GSM605330
hESC	ChIP-seq (H3K23ac)	GSM667618	hESC	ChIP-seq (H4K5ac)	GSM752990
hESC	ChIP-seq (H3K23me2)	GSM605305	hESC	ChIP-seq (H4K8ac)	GSM896166
hESC	ChIP-seq (H3K23me2)	GSM605306	hESC	ChIP-seq (H4K8ac)	GSM908966
hESC	ChIP-seq (H3K27ac)	GSM466732	hESC	ChIP-seq (H4K91ac)	GSM605332
hESC	ChIP-seq (H3K27ac)	GSM663427	hESC	ChIP-seq (H4K91ac)	GSM752991
hESC	ChIP-seq (H3K27me3)	GSM434776	hESC	RNA-Seq	GSM915328
hESC	ChIP-seq (H3K27me3)	GSM466734	hESC	RNA-Seq	GSM915329
hESC	ChIP-seq (H3K27me3)	GSM605308	IMR90	Bisulfite sequencing	GSM432687
hESC	ChIP-seq (H3K36me3)	GSM409312	IMR90	Bisulfite sequencing	GSM432688
hESC	ChIP-seq (H3K36me3)	GSM466737	IMR90	Bisulfite sequencing	GSM432689
hESC	ChIP-seq (H3K36me3)	GSM605309	IMR90	Bisulfite sequencing	GSM432690
hESC	ChIP-seq (H3K4ac)	GSM605311	IMR90	Bisulfite sequencing	GSM432691
hESC	ChIP-seq (H3K4ac)	GSM667624	IMR90	Bisulfite sequencing	GSM432692
hESC	ChIP-seq (H3K4me1)	GSM409307	IMR90	RNA-Seq	GSM438363

Supplementary figures

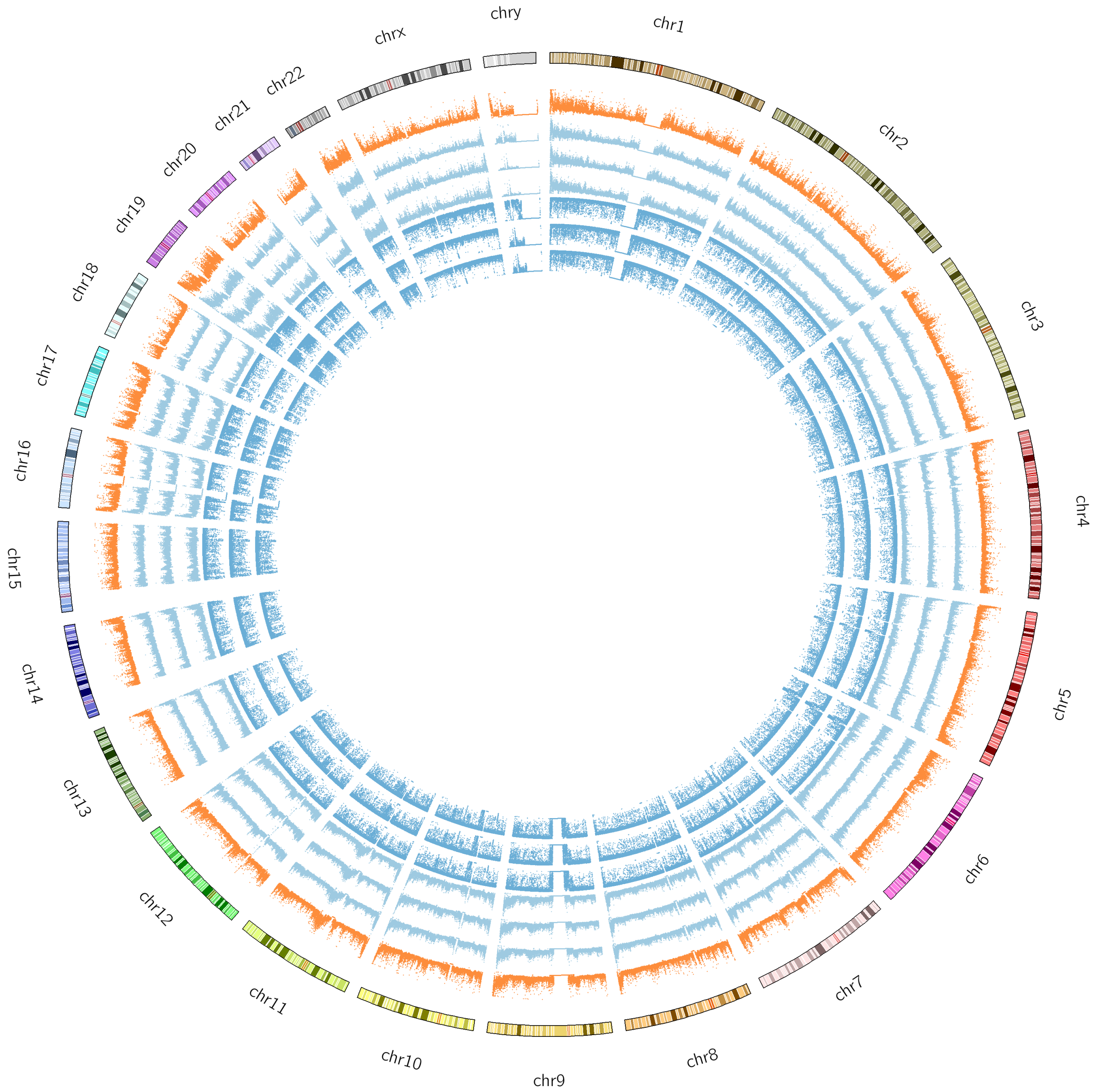

Figure S1: Genome-wide DNA methylation profiles of the three individuals based on 10kb sliding windows. The outermost track corresponds to the karyotype of the human genome. The remaining seven tracks correspond to, from outer to inner, in each window (1) the number of CpG dinucleotides, the number of methylated cytosines within CpG dinucleotides in (2) F, (3) M and (4) D, and the ratio of methylated cytosines as compared to the total number of CpG dinucleotides in (5) F, (6) M and (7) D.

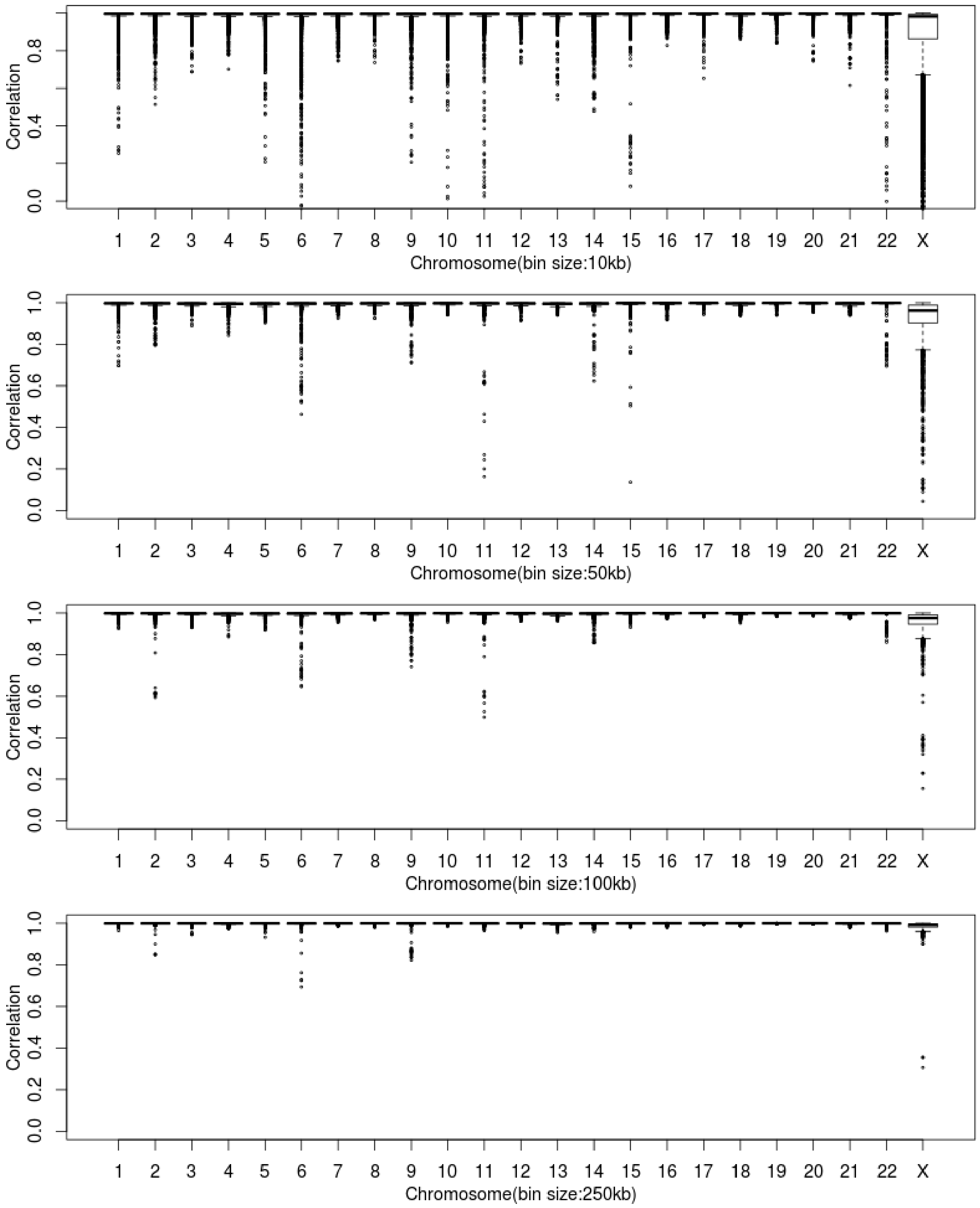

Figure S2: Genome-wide correlation values of DNA methylation levels between Father and Mother according to the mCG quantification measure. The correlation values are based on average methylation levels in every 15 consecutive windows. The four panels correspond to the results for windows of sizes 10kb, 50kb, 100kb and 250kb, respectively.

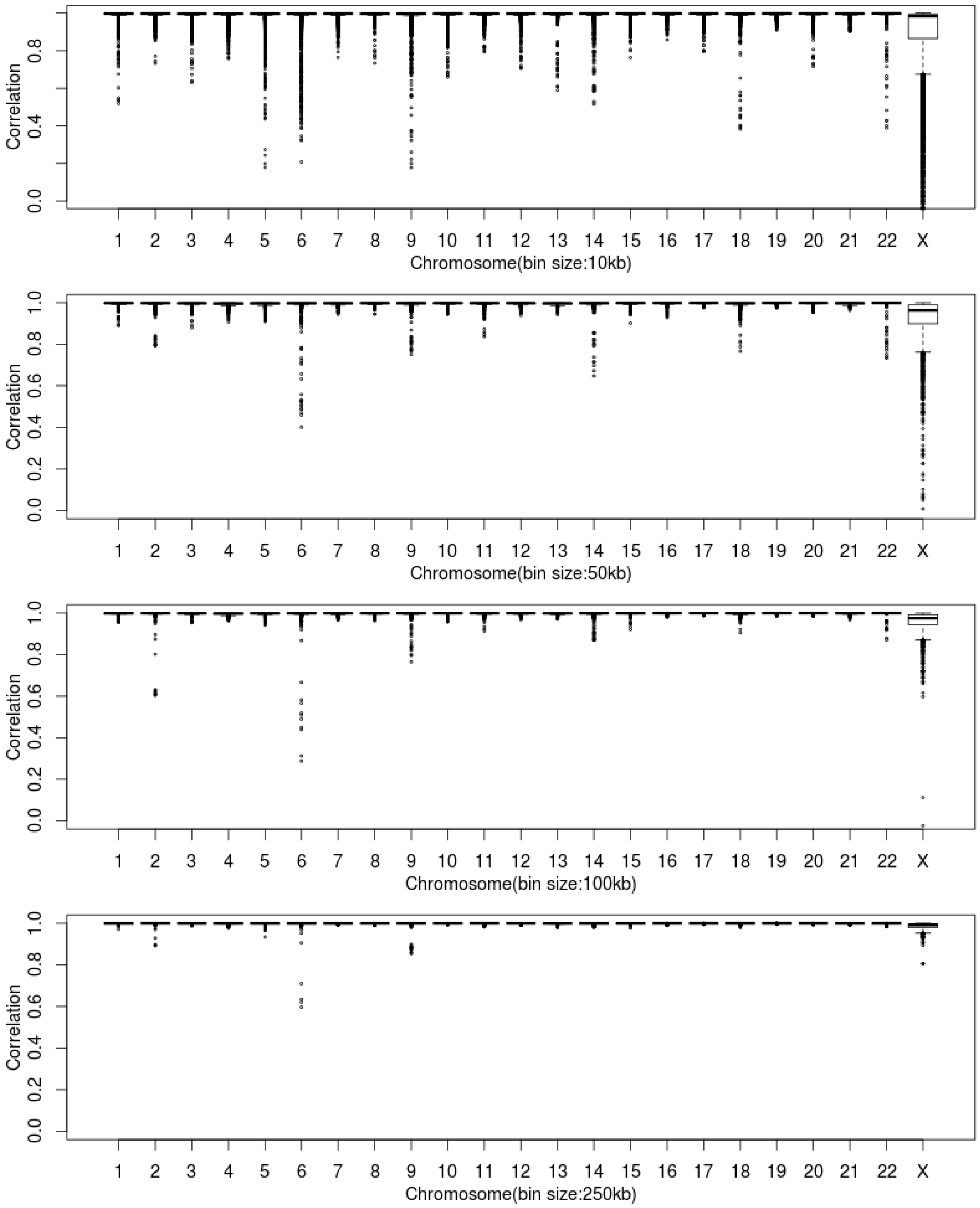

Figure S3: Genome-wide correlation values of DNA methylation levels between Father and Daughter according to the mCG quantification measure. The correlation values are based on average methylation levels in every 15 consecutive windows. The four panels correspond to the results for windows of sizes 10kb, 50kb, 100kb and 250kb, respectively.

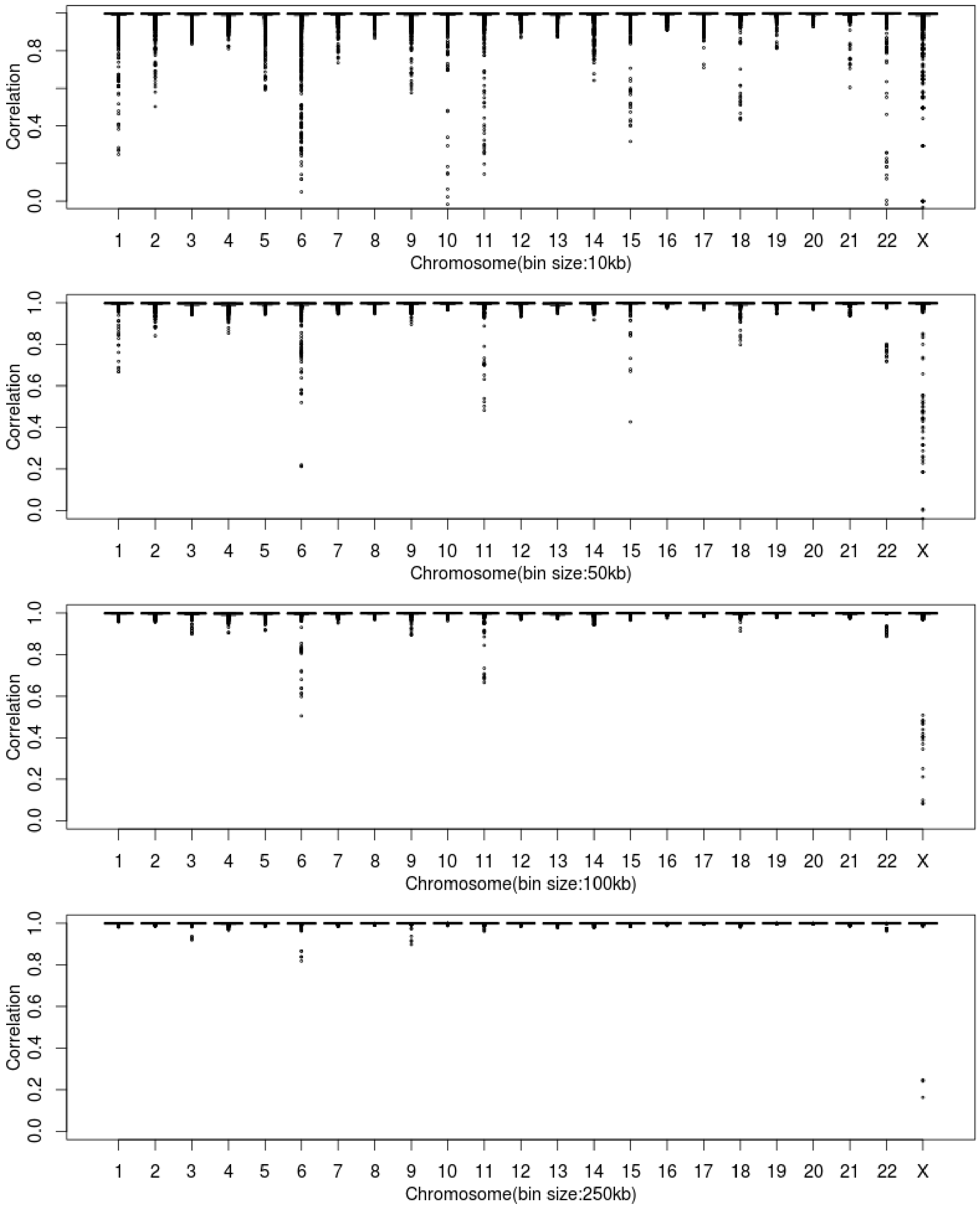

Figure S4: Genome-wide correlation values of DNA methylation levels between Mother and Daughter according to the mCG quantification measure. The correlation values are based on average methylation levels in every 15 consecutive windows. The four panels correspond to the results for windows of sizes 10kb, 50kb, 100kb and 250kb, respectively.

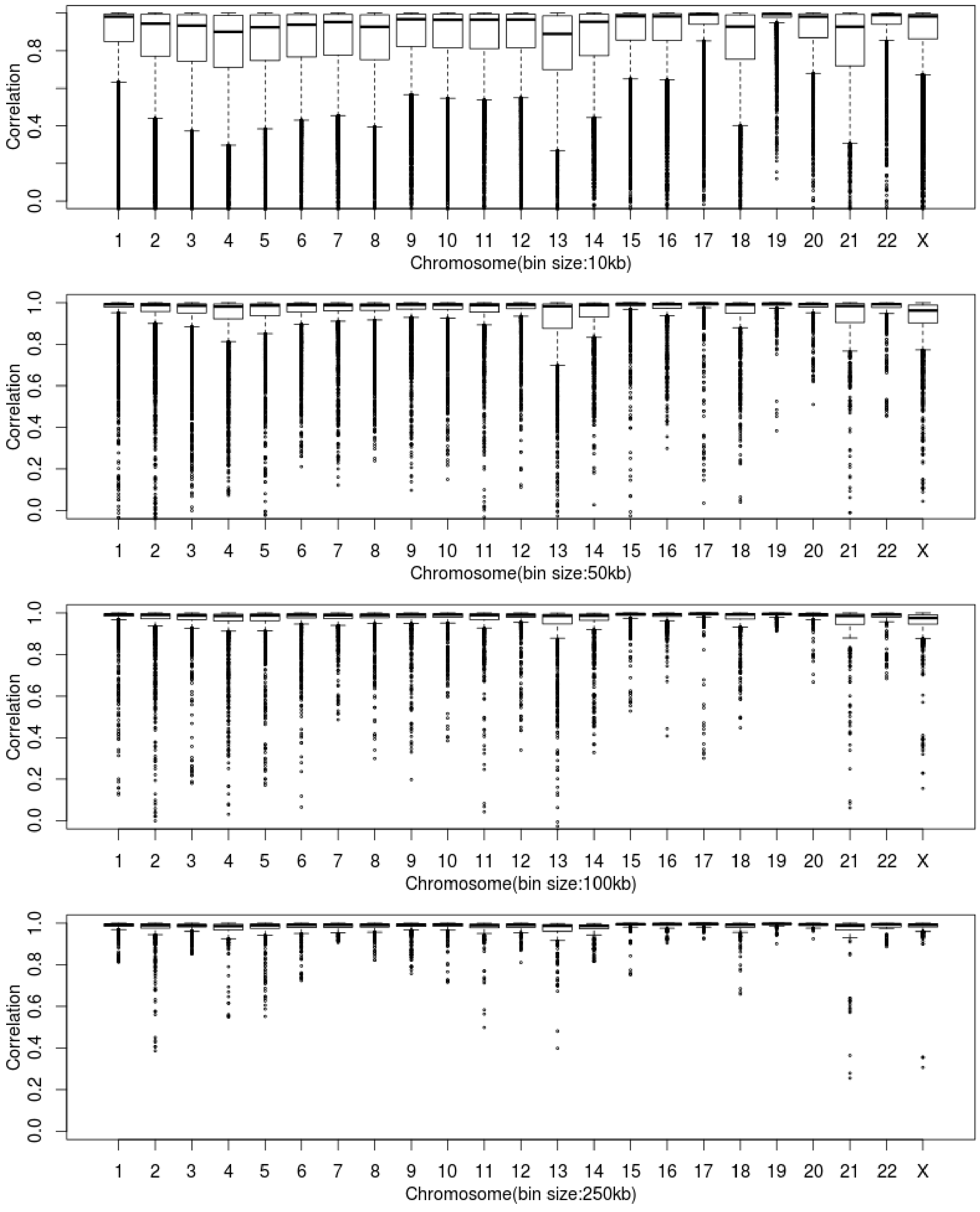

Figure S5: Genome-wide correlation values of DNA methylation levels between Father and Mother according to the mCG/CG quantification measure. The correlation values are based on average methylation levels in every 15 consecutive windows. The four panels correspond to the results for windows of sizes 10kb, 50kb, 100kb and 250kb, respectively.

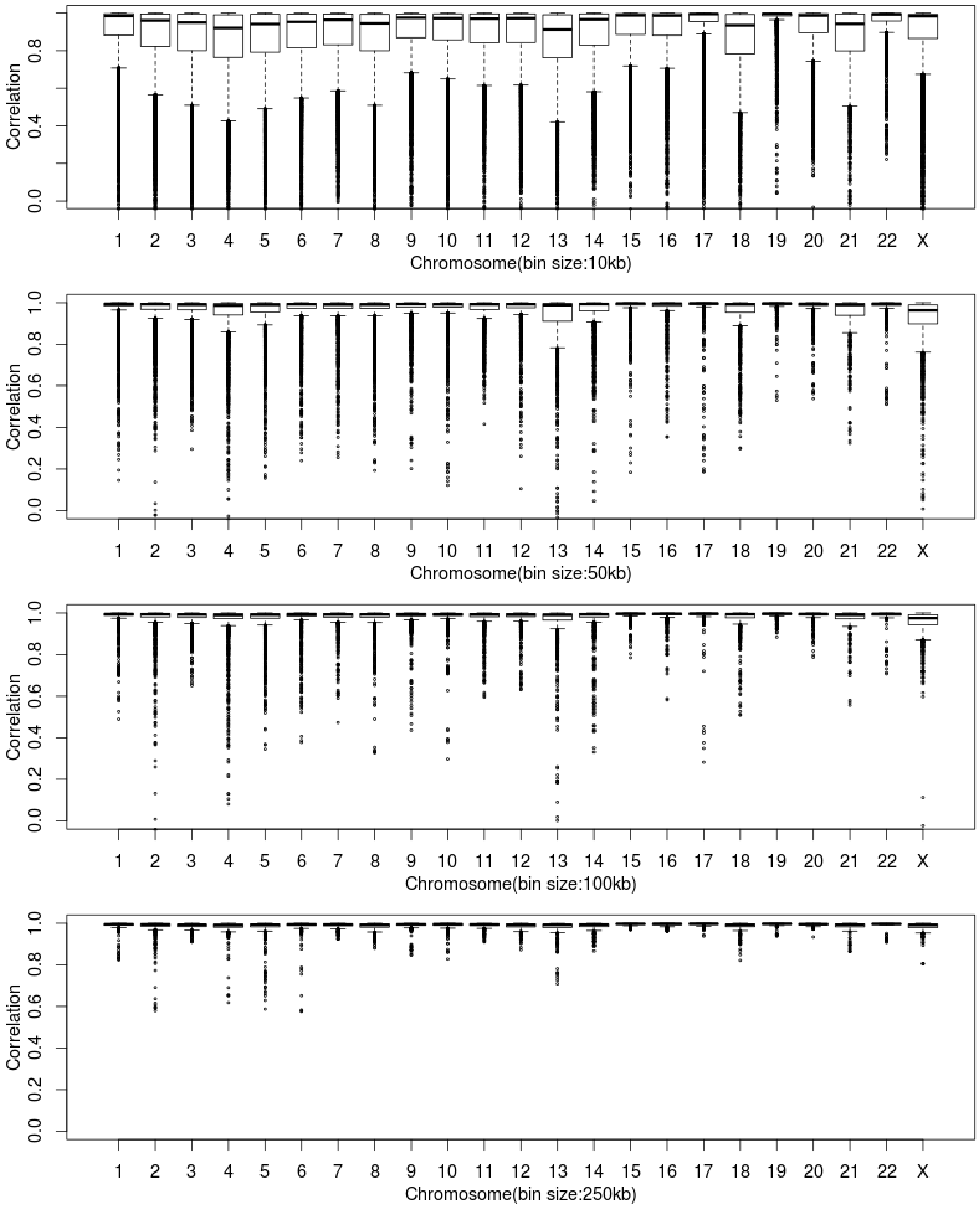

Figure S6: Genome-wide correlation values of DNA methylation levels between Father and Daughter according to the mCG/CG quantification measure. The correlation values are based on average methylation levels in every 15 consecutive windows. The four panels correspond to the results for windows of sizes 10kb, 50kb, 100kb and 250kb, respectively.

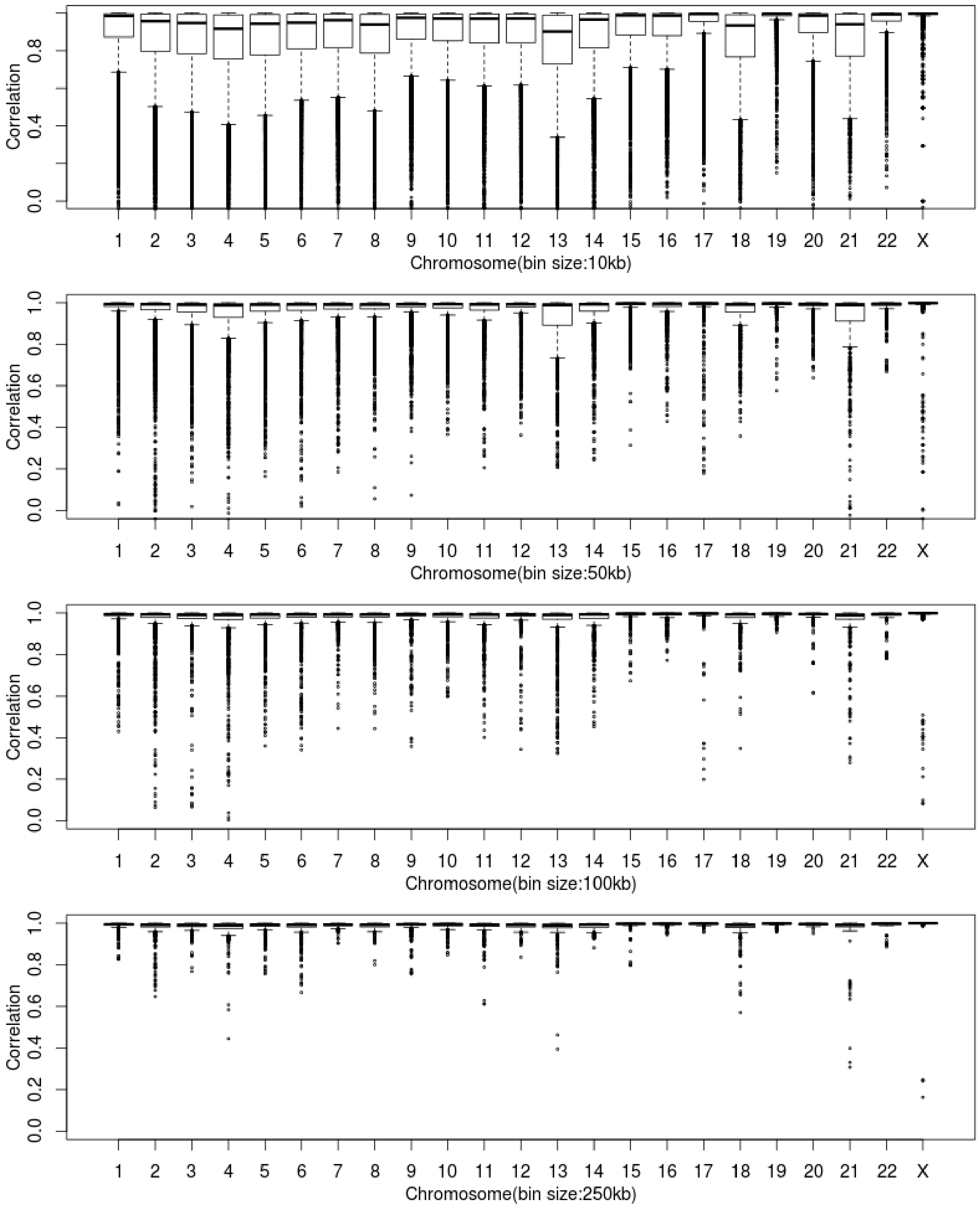

Figure S7: Genome-wide correlation values of DNA methylation levels between Mother and Daughter according to the mCG/CG quantification measure. The correlation values are based on average methylation levels in every 15 consecutive windows. The four panels correspond to the results for windows of sizes 10kb, 50kb, 100kb and 250kb, respectively.

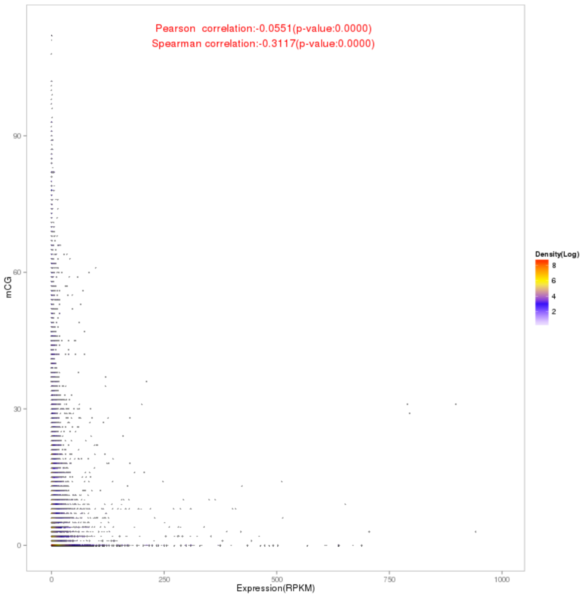

(a) mCG

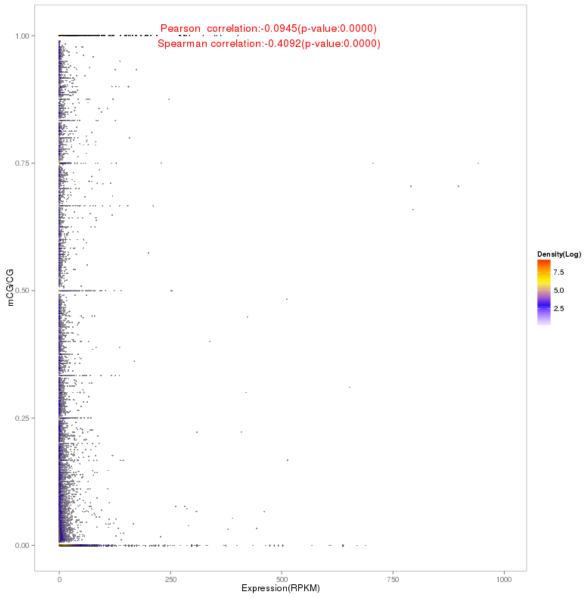

(b) mCG/CG

Figure S8: Relationships between the DNA methylation and expression levels of gene upstream regions. Each point in the figure corresponds to a gene. The methylation of a gene is the average level over its 2kb upstream region. The two panels correspond to the results based on the mCG and mCG/CG DNA methylation measures. Since the upstream regions were defined to have the same length for all genes, the plots for mCG/len and mCG/CG/len would be identical to those for mCG and mCG/CG, respectively, and are thus omitted. Color indicates number of points (in $\log_2$ scale) within a cell when the occupied space is divided into a 500x500 grid.

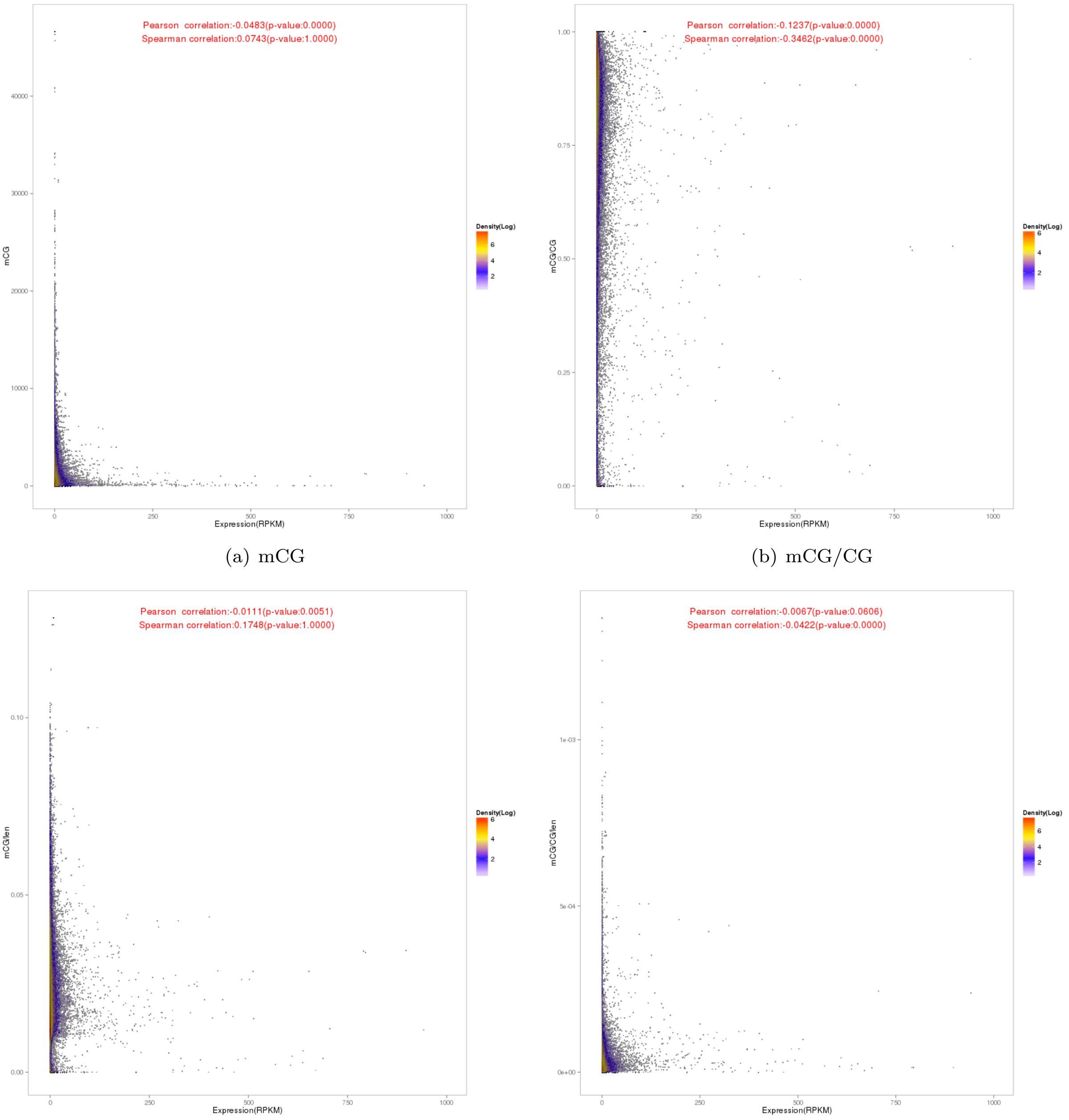

(a) mCG

(b) mCG/CG

(c) mCG/len

(d) mCG/CG/len

Figure S9: Relationships between the DNA methylation and expression levels of gene bodies. Each point in the figure corresponds to a gene. The methylation of a gene is the average level over its transcribed region. The four panels correspond to the results based on four different DNA methylation measures. Color indicates number of points (in $\log_2$ scale) within a cell when the occupied space is divided into a 500x500 grid.

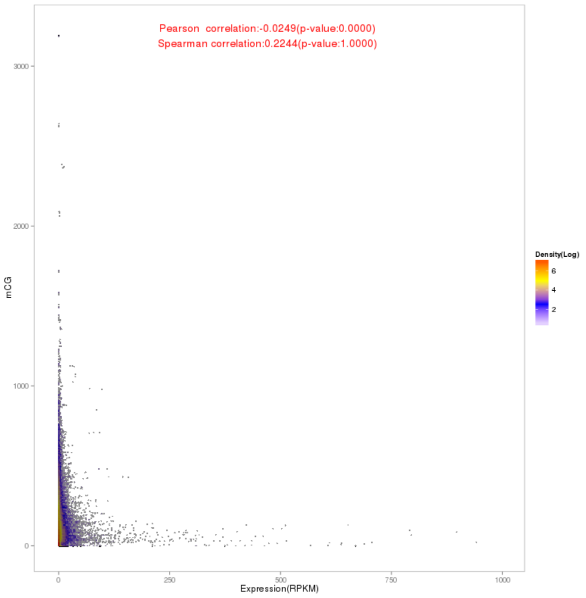

(a) mCG

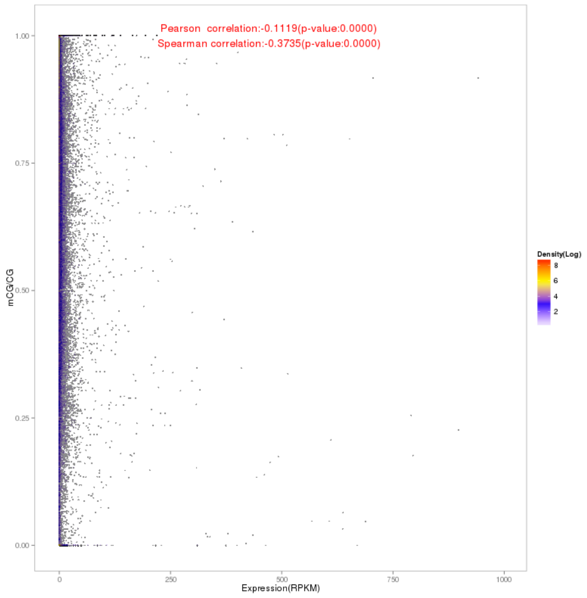

(b) mCG/CG

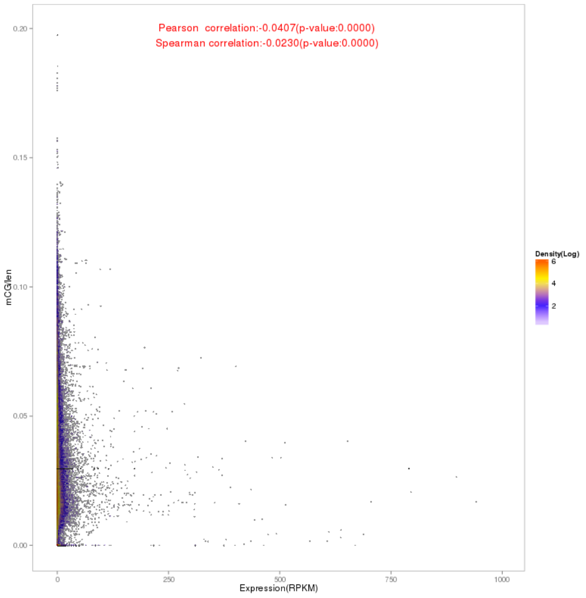

(c) mCG/len

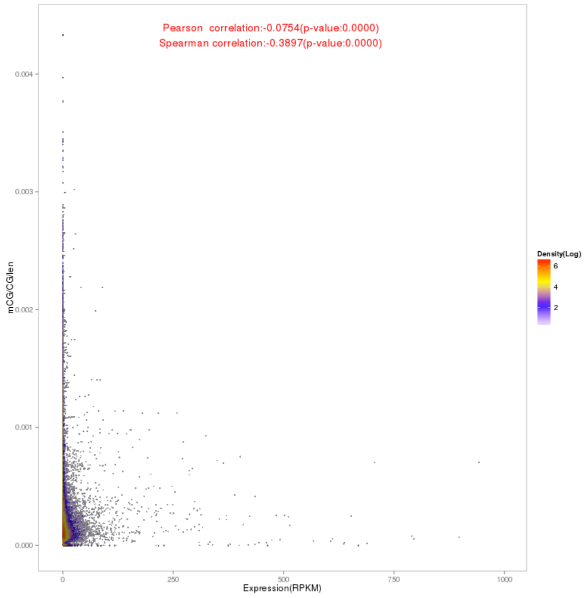

(d) mCG/CG/len

Figure S10: Relationships between the DNA methylation and expression levels of gene exons. Each point in the figure corresponds to a gene. The methylation of a gene is the average level over its exonic regions. The four panels correspond to the results based on four different DNA methylation measures. Color indicates number of points (in $\log_2$ scale) within a cell when the occupied space is divided into a 500x500 grid.

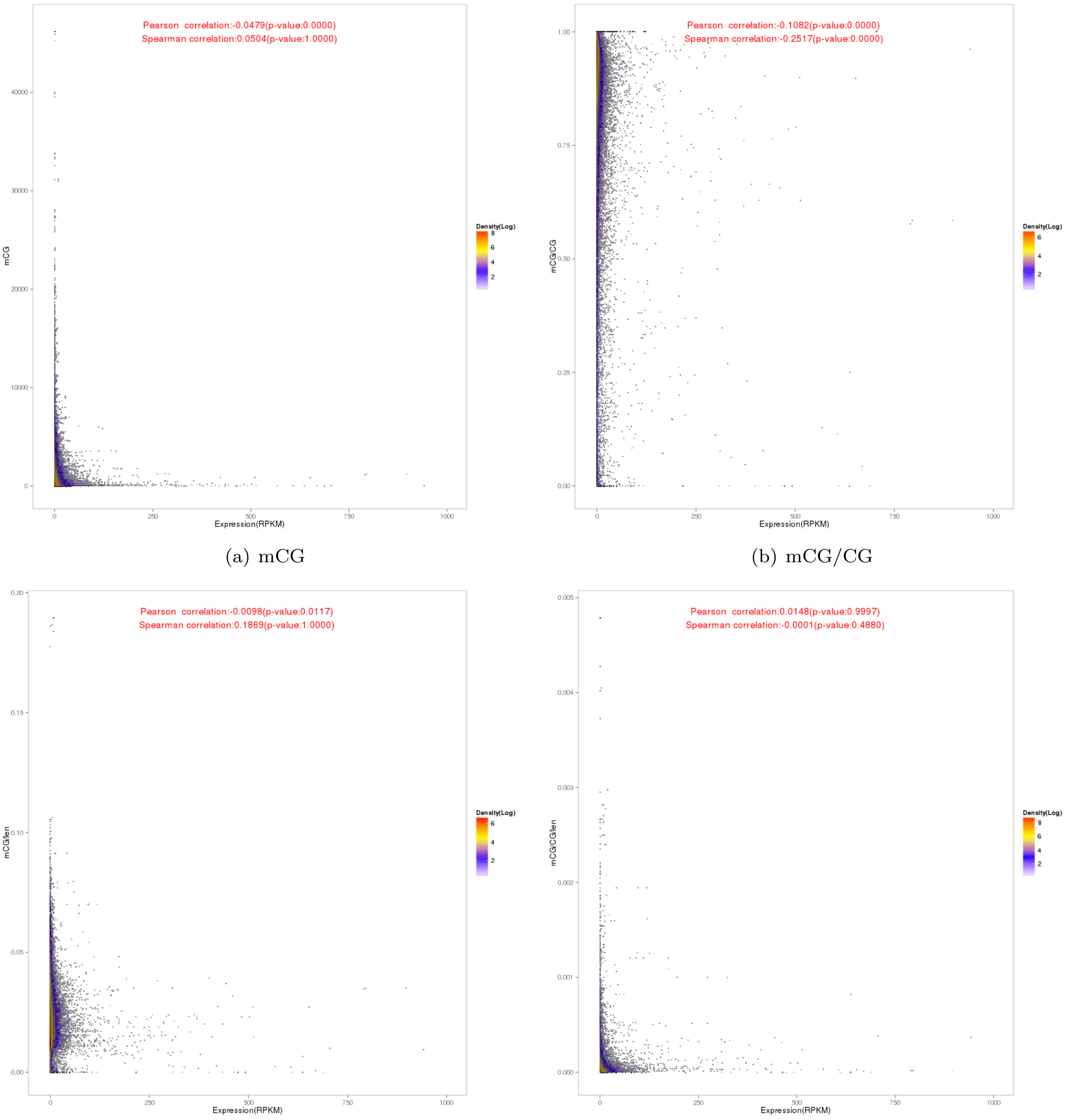

(a) mCG

(b) mCG/CG

(c) mCG/len

(d) mCG/CG/len

Figure S11: Relationships between the DNA methylation and expression levels of gene introns. Each point in the figure corresponds to a gene. The methylation of a gene is the average level over its intronic regions. The four panels correspond to the results based on four different DNA methylation measures. Color indicates number of points (in $\log_2$ scale) within a cell when the occupied space is divided into a 500x500 grid.

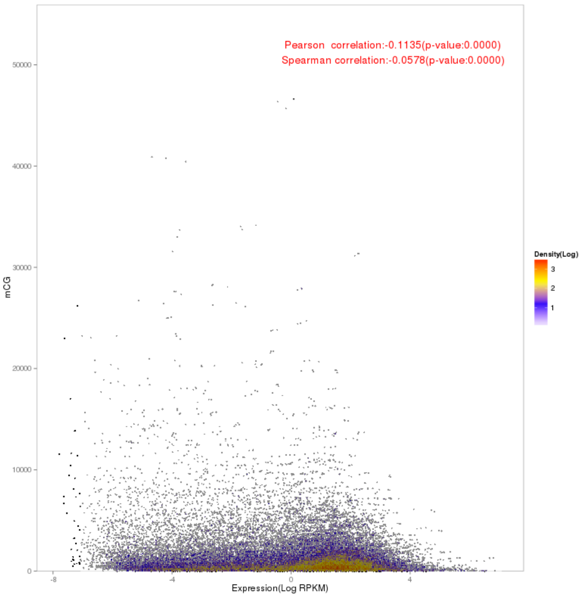

(a) mCG

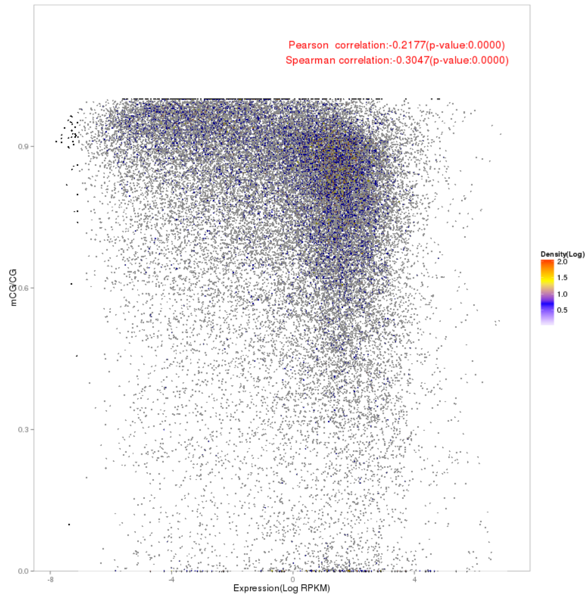

(b) mCG/CG

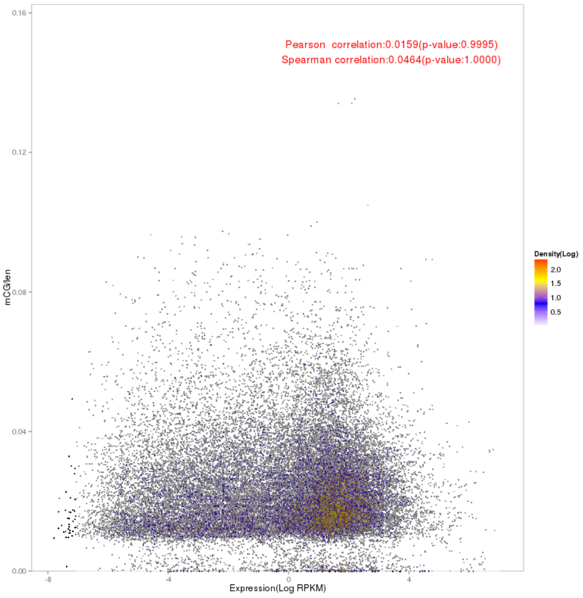

(c) mCG/len

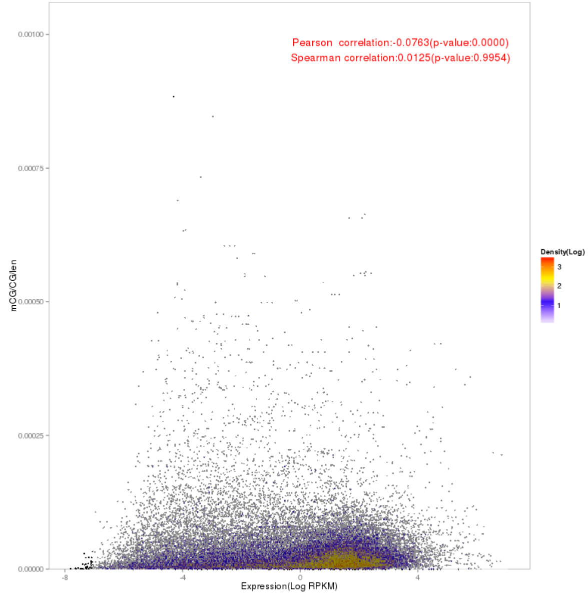

(d) mCG/CG/len

Figure S12: Relationships between the DNA methylation and log expression levels of genes. Each point in the figure corresponds to a gene. The methylation of a gene is the average level over its body and 2kb upstream region. The four panels correspond to the results based on four different DNA methylation measures. Color indicates number of points (in $\log_2$ scale) within a cell when the occupied space is divided into a 500x500 grid.

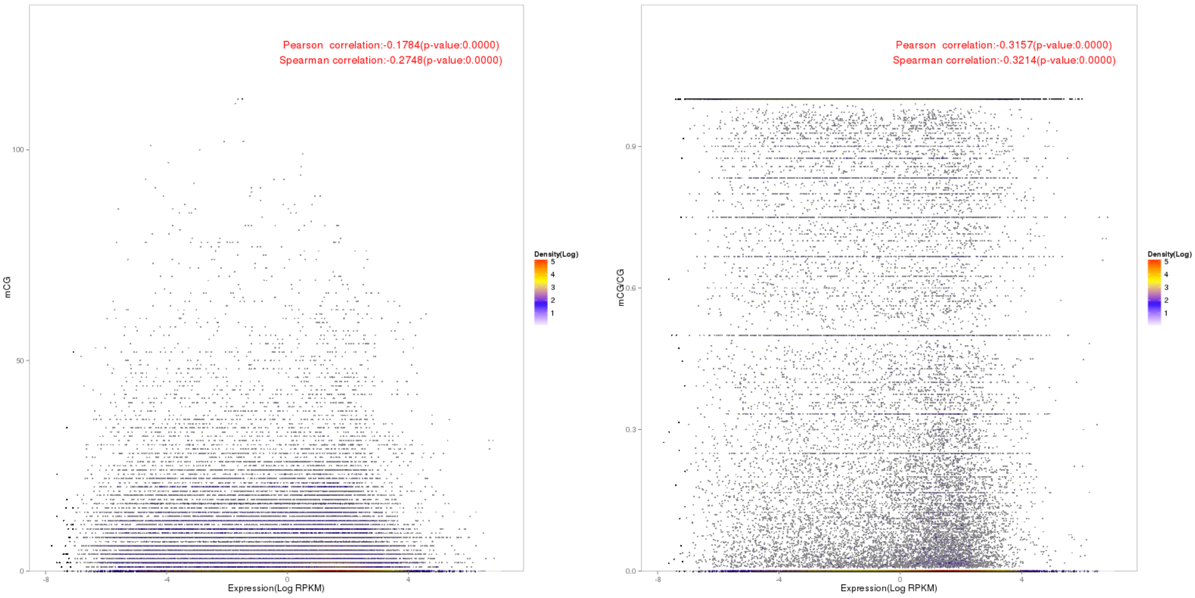

(a) mCG (b) mCG/CG

Figure S13: Relationships between the DNA methylation and log expression levels of gene upstream regions. Each point in the figure corresponds to a gene. The methylation of a gene is the average level over its 2kb upstream region. The two panels correspond to the results based on the mCG and mCG/CG DNA methylation measures. Since the upstream regions were defined to have the same length for all genes, the plots for mCG/len and mCG/CG/len would be identical to those for mCG and mCG/CG, respectively, and are thus omitted. Color indicates number of points (in $\log_2$ scale) within a cell when the occupied space is divided into a 500x500 grid.

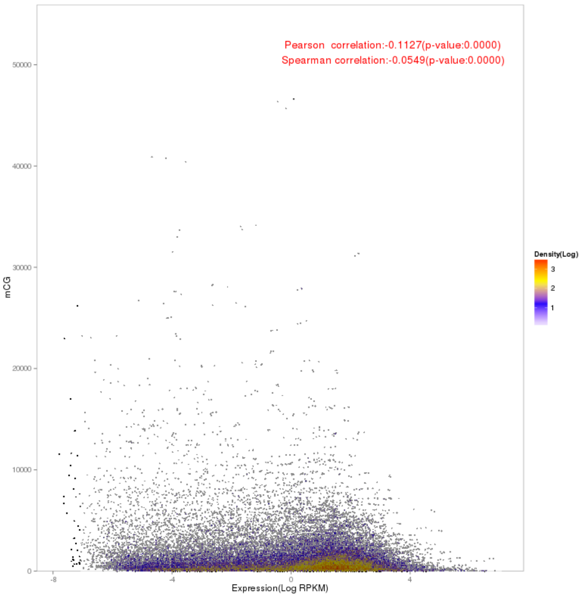

(a) mCG

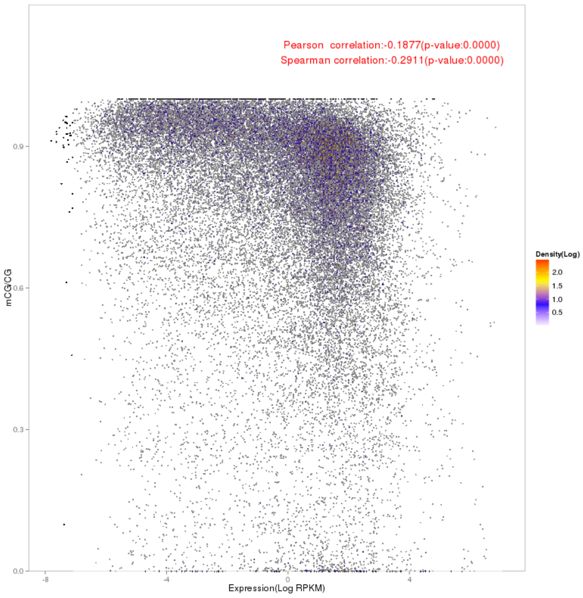

(b) mCG/CG

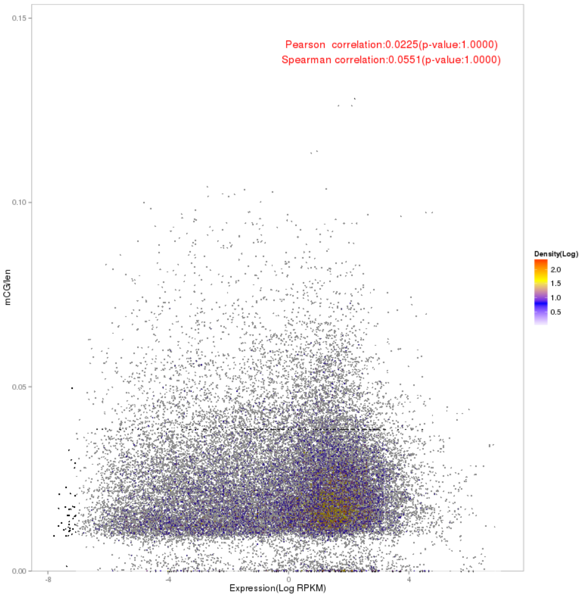

(c) mCG/len

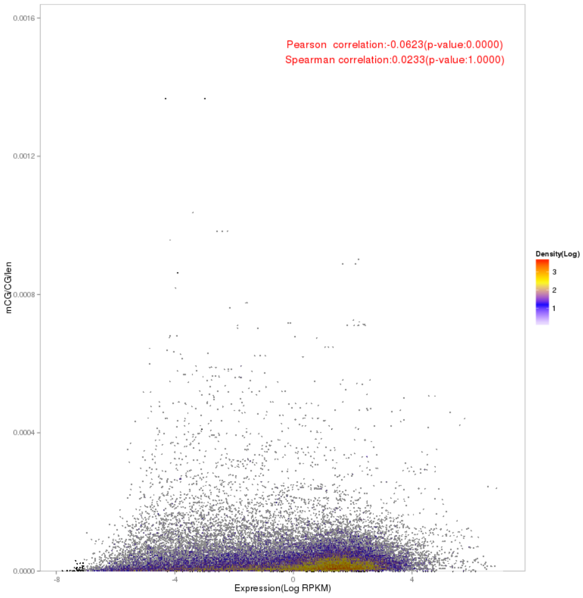

(d) mCG/CG/len

Figure S14: Relationships between the DNA methylation and log expression levels of gene bodies. Each point in the figure corresponds to a gene. The methylation of a gene is the average level over its transcribed region. The four panels correspond to the results based on four different DNA methylation measures. Color indicates number of points (in $\log_2$ scale) within a cell when the occupied space is divided into a 500x500 grid.

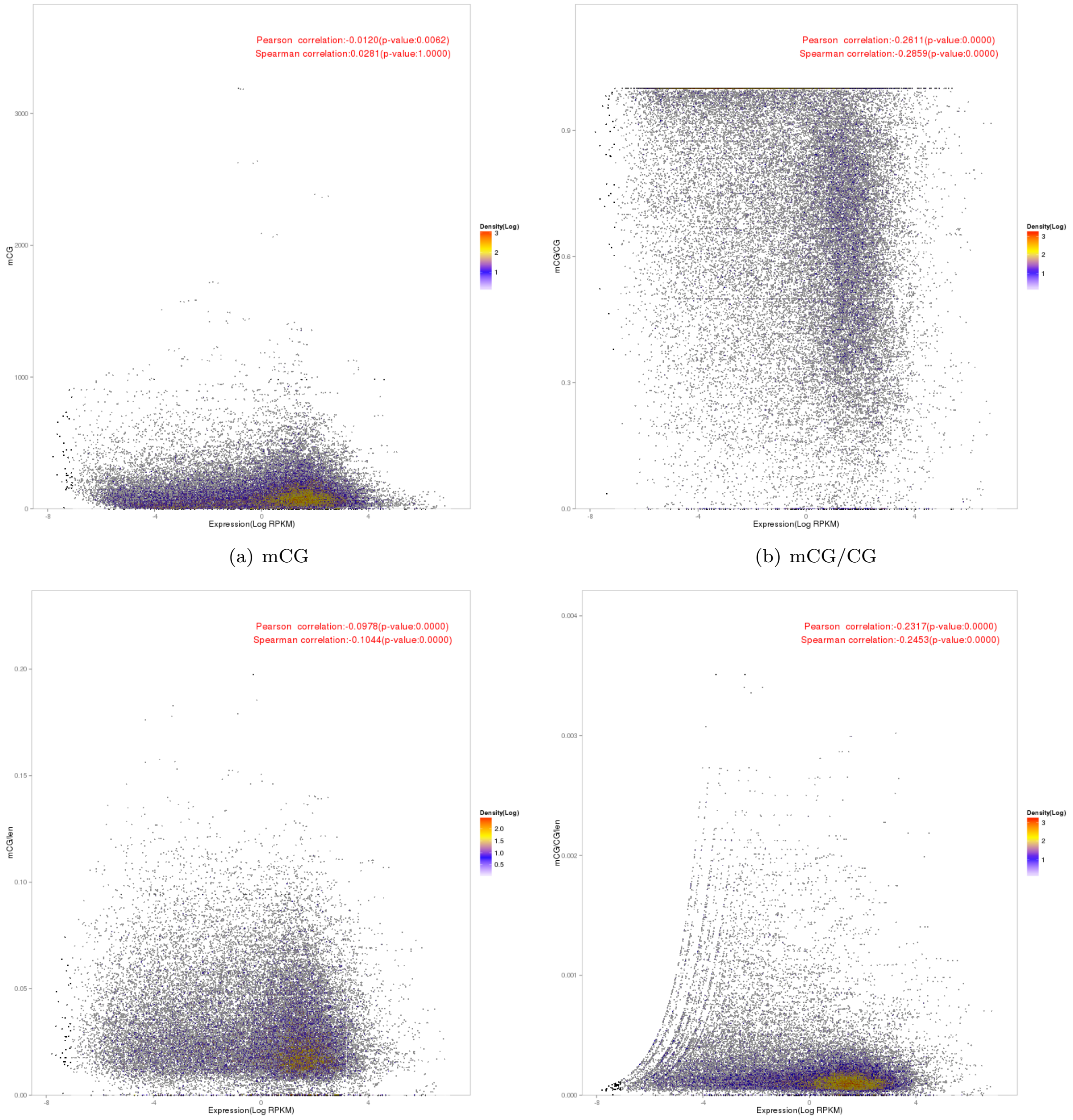

(a) mCG

(b) mCG/CG

(c) mCG/len

(d) mCG/CG/len

Figure S15: Relationships between the DNA methylation and log expression levels of gene exons. Each point in the figure corresponds to a gene. The methylation of a gene is the average level over its exonic regions. The four panels correspond to the results based on four different DNA methylation measures. Color indicates number of points (in $\log_2$ scale) within a cell when the occupied space is divided into a 500x500 grid.

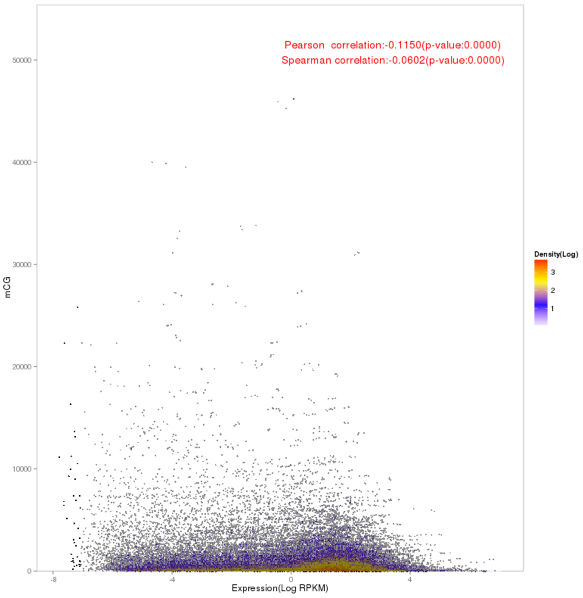

(a) mCG

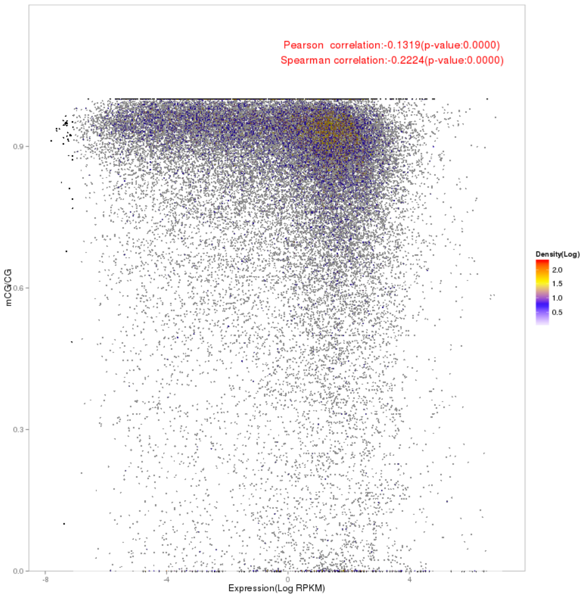

(b) mCG/CG

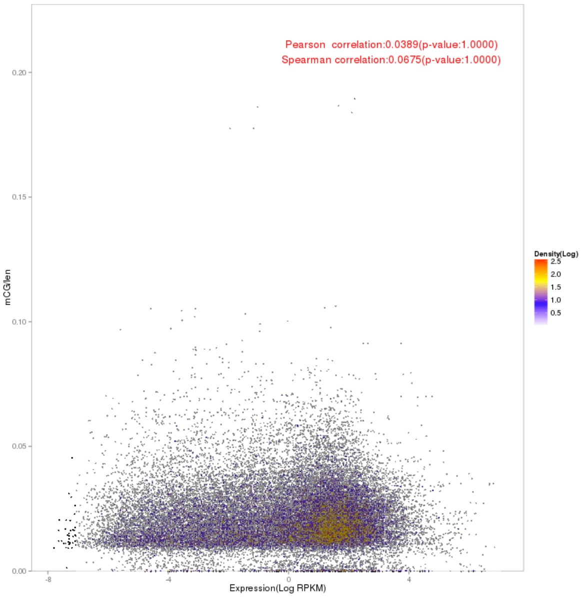

(c) mCG/len

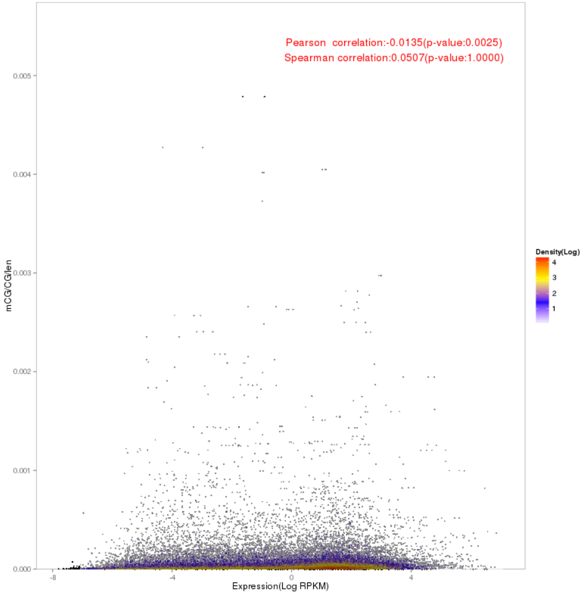

(d) mCG/CG/len

Figure S16: Relationships between the DNA methylation and log expression levels of gene introns. Each point in the figure corresponds to a gene. The methylation of a gene is the average level over its intronic regions. The four panels correspond to the results based on four different DNA methylation measures. Color indicates number of points (in $\log_2$ scale) within a cell when the occupied space is divided into a 500x500 grid.

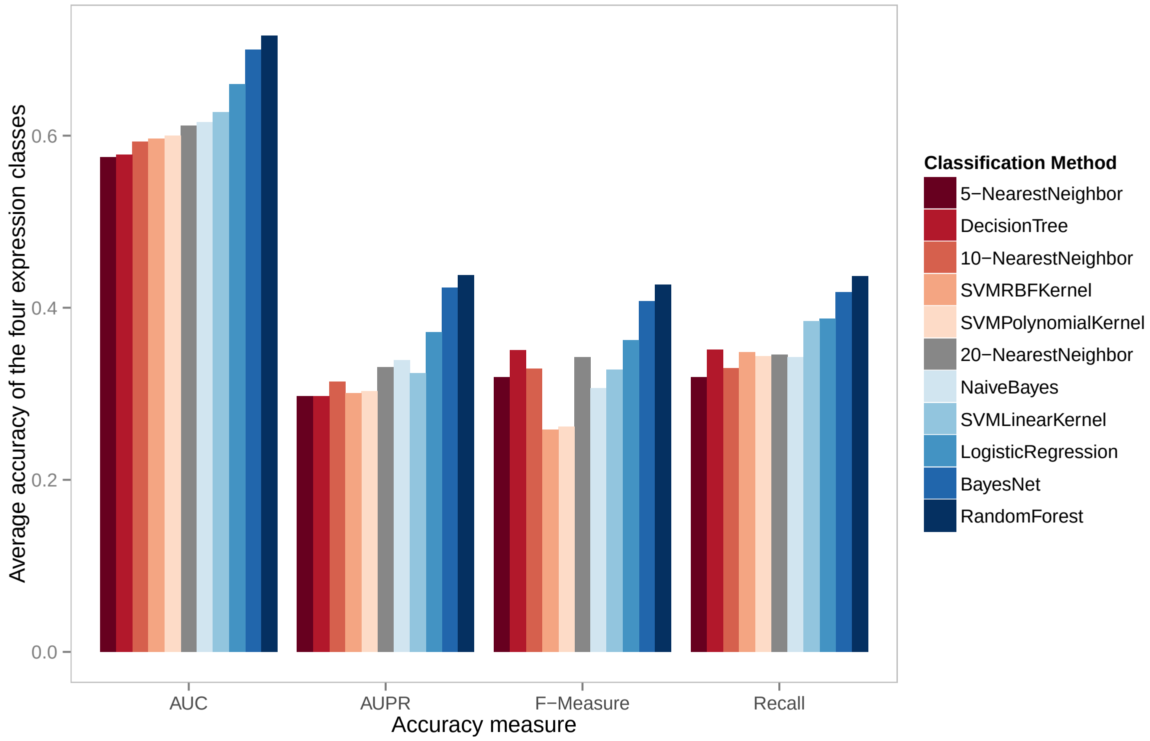

Figure S17: Average accuracy of the different model construction methods. The accuracy of a method is defined as its average accuracy over the four gene expression classes. The four bar groups correspond to four different ways to compute model accuracy. The different methods are ordered according to their accuracy based on the AUC measure, so that the method receiving the lowest AUC value (5-Nearest Neighbor) is ordered first in all four bar groups, followed by the method receiving the second lowest AUC value (Decision Tree), and so on.

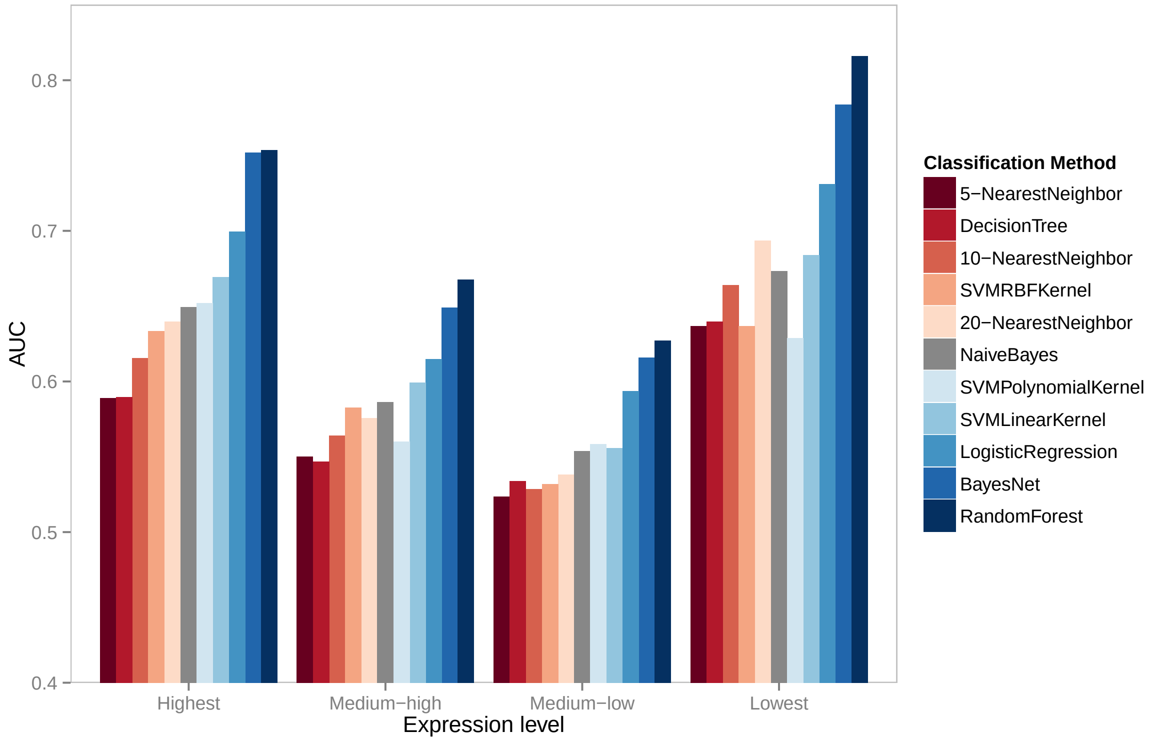

Figure S18: Accuracy of the different model construction methods on genes from different expression classes. The accuracy of a method is defined based on the AUC measure. The first four bar groups correspond to the four gene expression classes, while the last one shows the average accuracy of them.

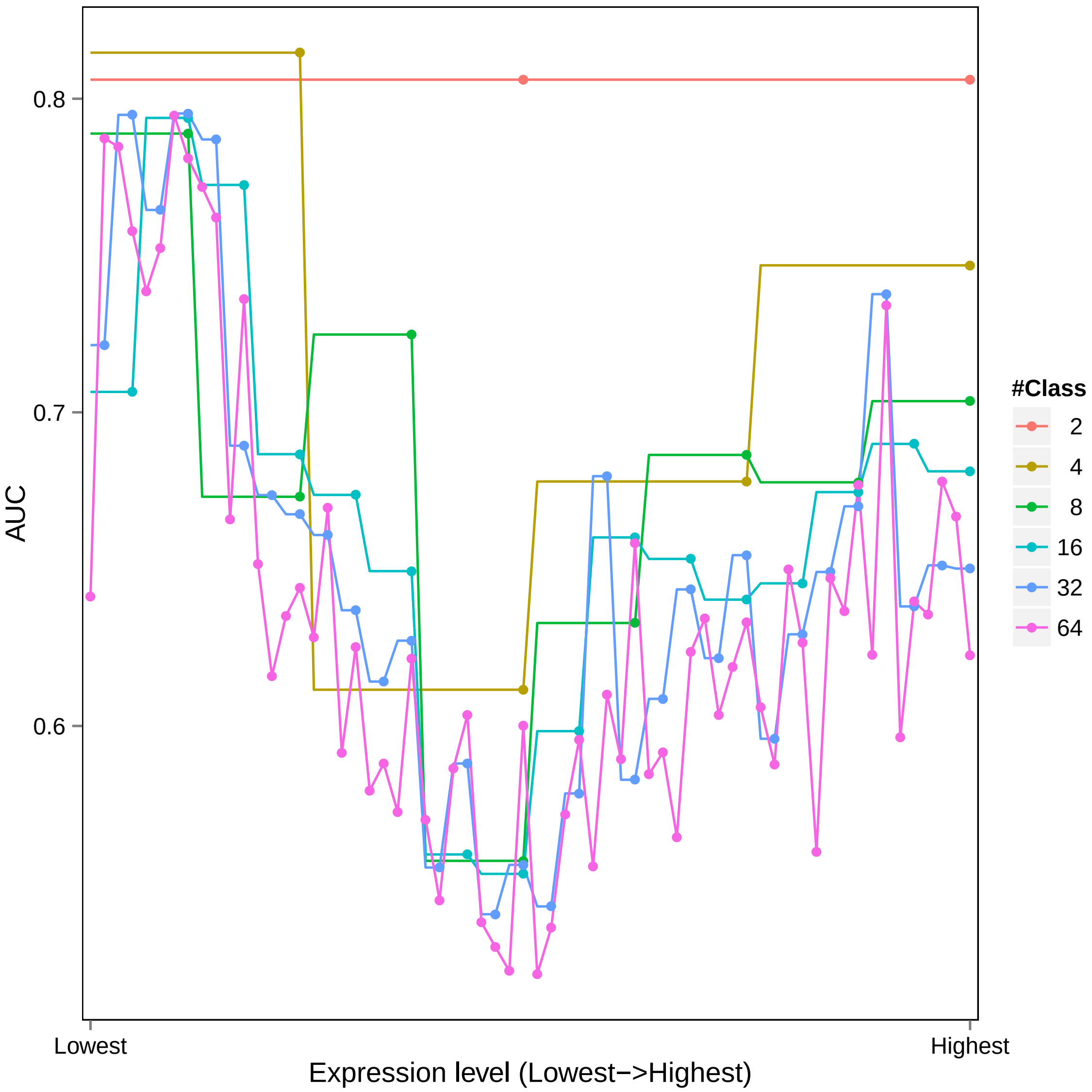

Figure S19: Accuracy of Random Forest models with different number of expression classes, based on DNA methylation features quantified by the mCG measure. Each curve corresponds to the results of a fixed number of expression classes. In each curve, the different expression classes are represented by different values along the x-axis, where the leftmost value corresponds to the class with lowest expression and the rightmost value corresponds to the class with highest expression. The y-coordinate of each class is the average modeling accuracy (AUC) based on a 10-fold cross validation procedure.

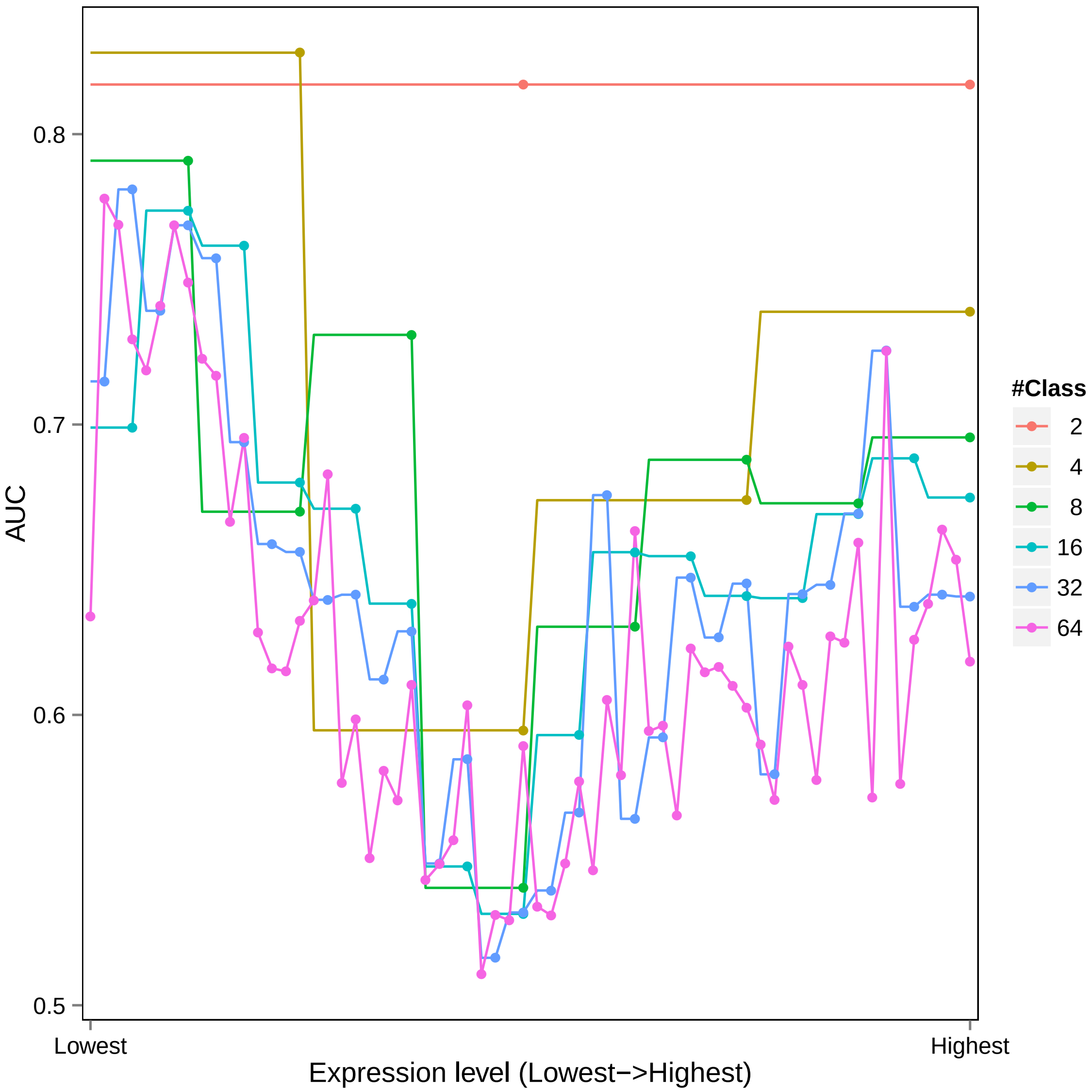

Figure S20: Accuracy of Random Forest models with different number of expression classes, based on DNA methylation features quantified by the mCG/CG measure. Each curve corresponds to the results of a fixed number of expression classes. In each curve, the different expression classes are represented by different values along the x-axis, where the leftmost value corresponds to the class with lowest expression and the rightmost value corresponds to the class with highest expression. The y-coordinate of each class is the average modeling accuracy (AUC) based on a 10-fold cross validation procedure.

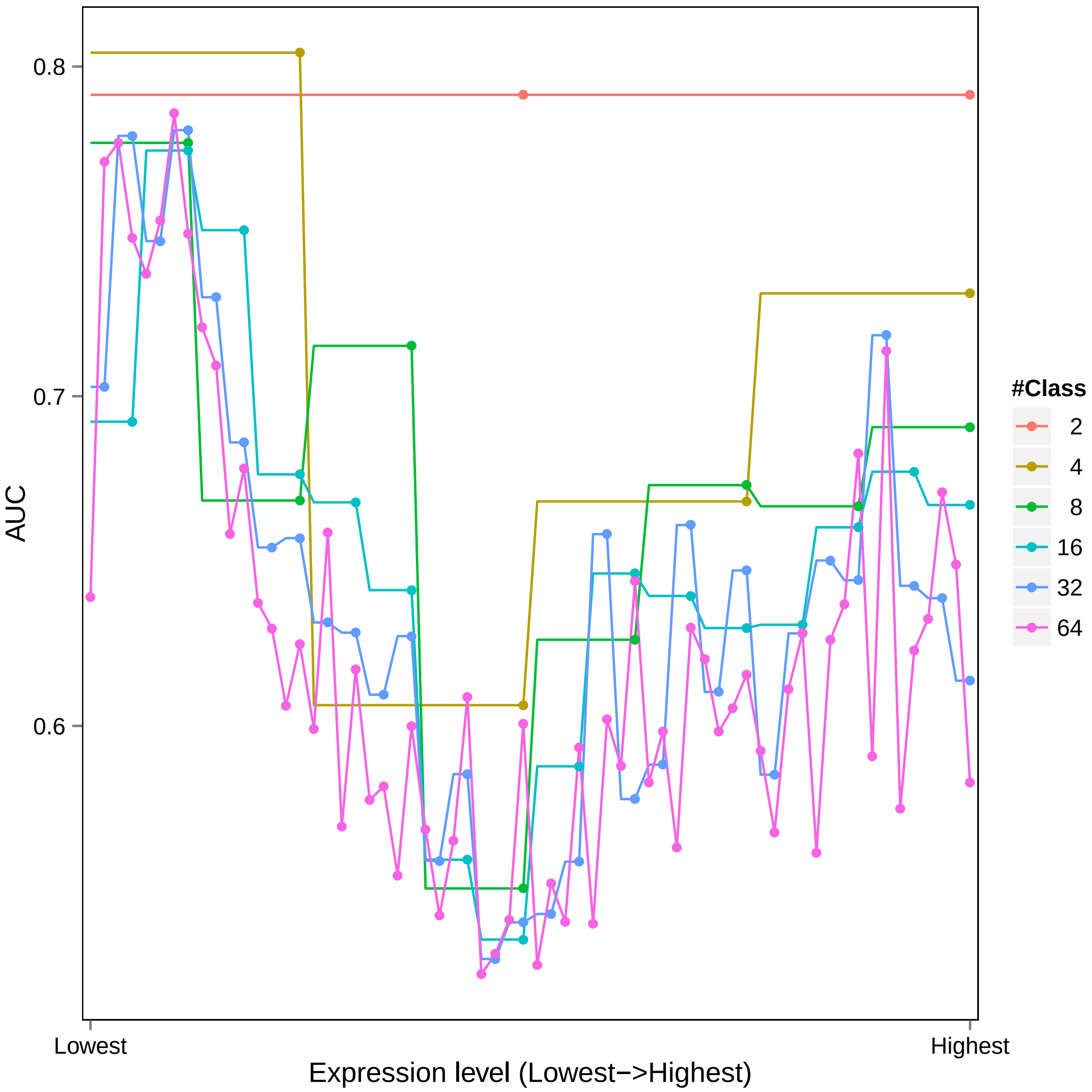

Figure S21: Accuracy of Random Forest models with different number of expression classes, based on DNA methylation features quantified by the mCG/len measure. Each curve corresponds to the results of a fixed number of expression classes. In each curve, the different expression classes are represented by different values along the x-axis, where the leftmost value corresponds to the class with lowest expression and the rightmost value corresponds to the class with highest expression. The y-coordinate of each class is the average modeling accuracy (AUC) based on a 10-fold cross validation procedure.

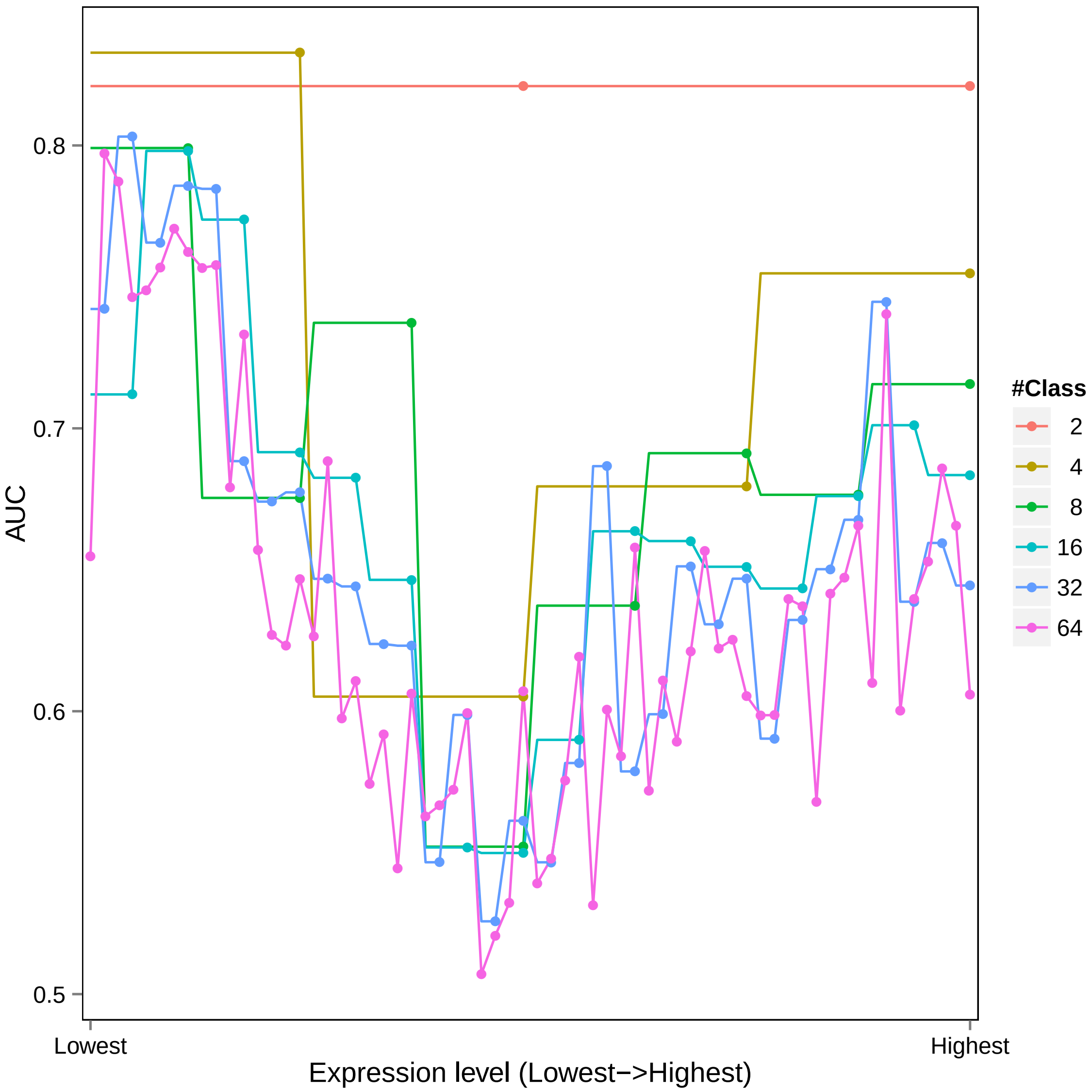

Figure S22: Accuracy of Random Forest expression models with different number of expression classes, based on DNA methylation features quantified by the mCG/CG/len measure. Each curve corresponds to the results of a fixed number of expression classes. In each curve, the different expression classes are represented by different values along the x-axis, where the leftmost value corresponds to the class with lowest expression and the rightmost value corresponds to the class with highest expression. The y-coordinate of each class is the average modeling accuracy (AUC) based on a 10-fold cross validation procedure.

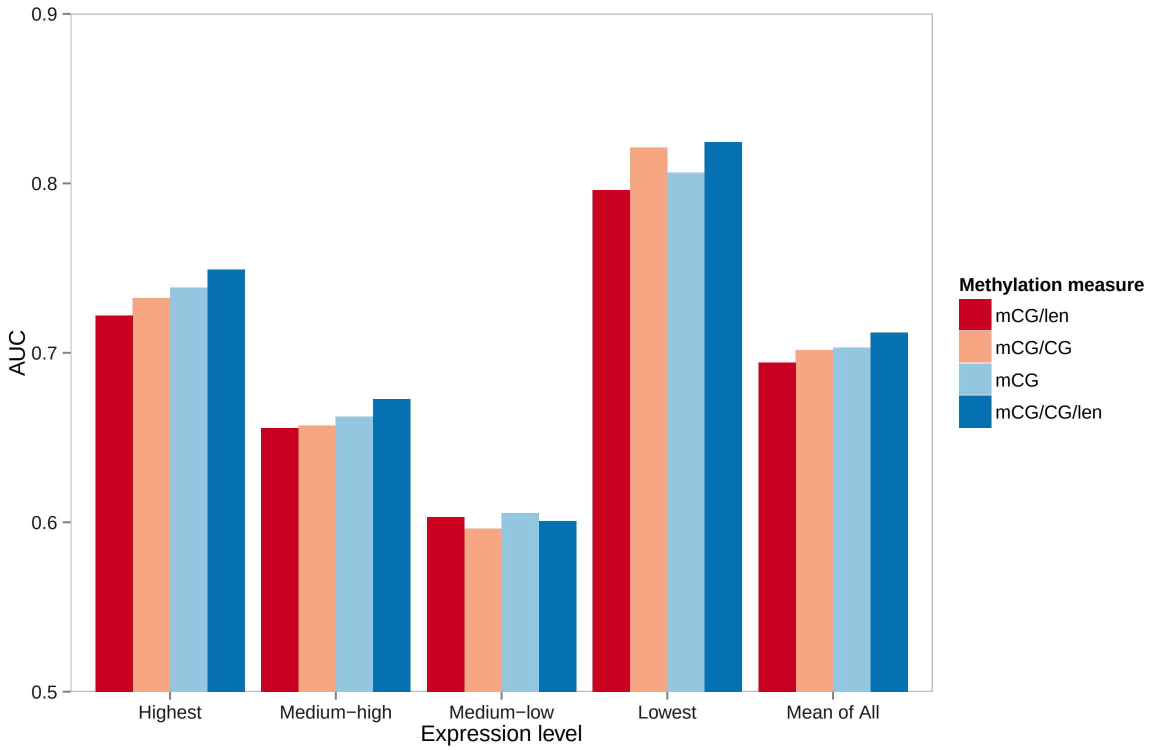

Figure S23: Accuracy of Random Forest expression models for different expression classes based on different DNA methylation measures. The first four bar groups correspond to the results for the four expression classes, while the last one shows the average accuracy of them. Within each bar group, the four bars correspond to the models based on DNA methylation features derived according to different quantification measures, ordered according to their average accuracy.

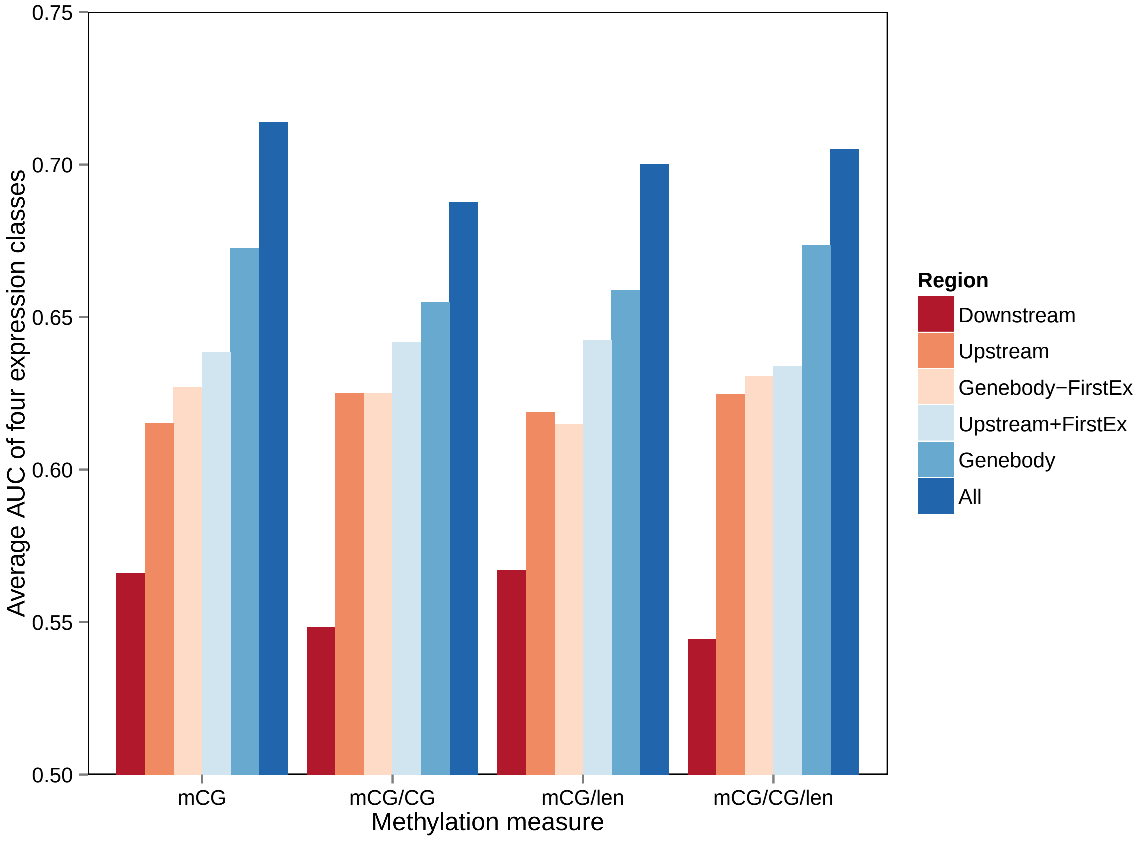

Figure S24: Accuracy of Random Forest expression models for genes with only one annotated transcript isoform. The different bar groups correspond to the models constructed according to DNA methylation features computed by different quantification measures. Within each bar group the different bars compare the accuracy of the models constructed from different feature sets. Body-FirstEx corresponds to the set of features from transcribed sub-regions excluding the first exon. Upstream+FirstEx corresponds to the set of features from both the 2kb upstream region and the first exon.

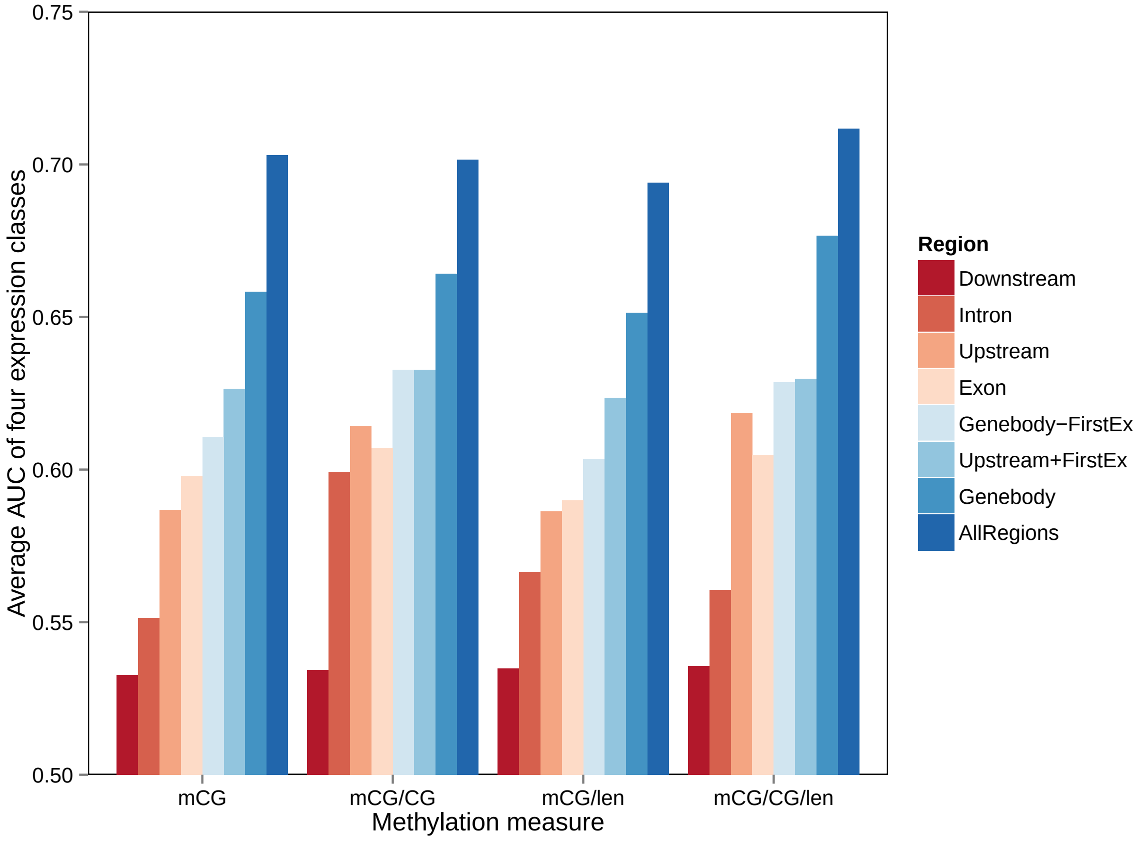

Figure S25: Accuracy of Random Forest expression models for all annotated genes when each expression class has the same number of genes. The different bar groups correspond to the models constructed according to DNA methylation features computed by different quantification measures. Within each bar group the different bars compare the accuracy of the models constructed from different feature sets. Body-FirstEx corresponds to the set of features from transcribed sub-regions excluding the first exon. Upstream+FirstEx corresponds to the set of features from both the 2kb upstream region and the first exon.

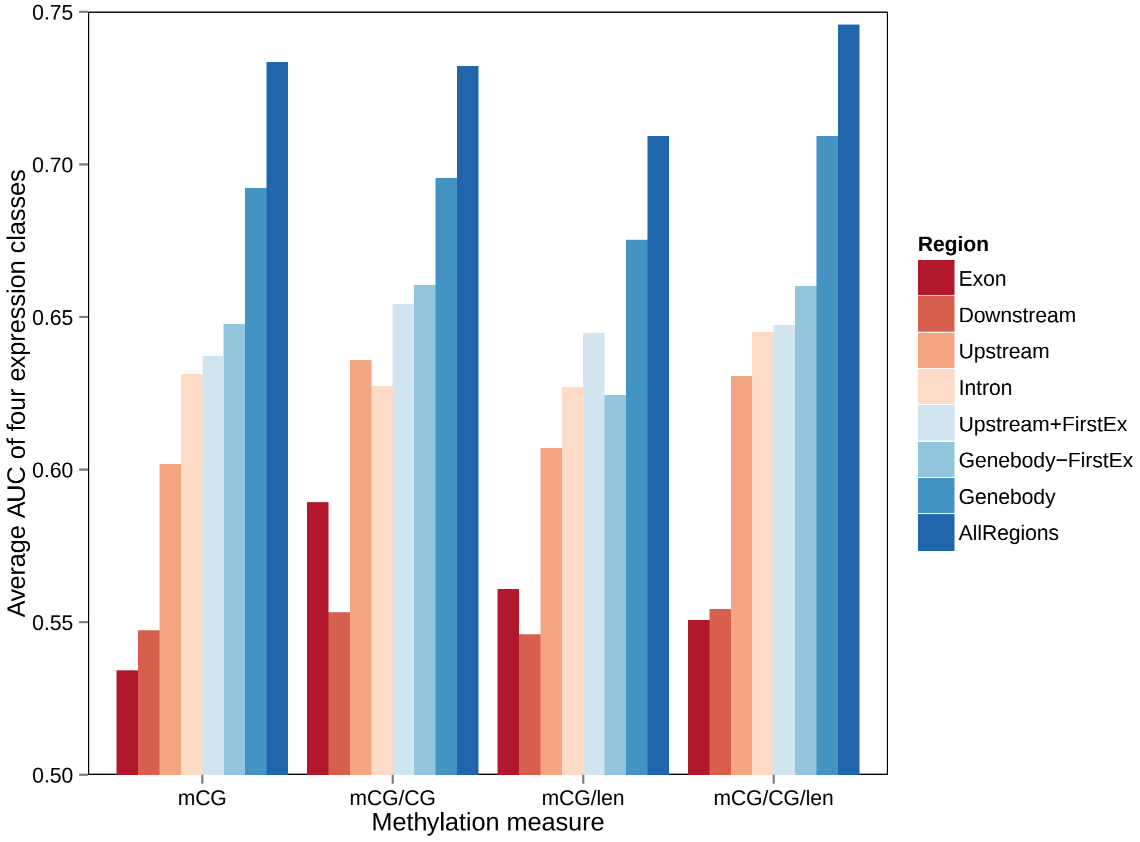

Figure S26: Accuracy of Random Forest expression models for all annotated genes when each expression class covers the same range of log-expression values. The different bar groups correspond to the models constructed according to DNA methylation features computed by different quantification measures. Within each bar group the different bars compare the accuracy of the models constructed from different feature sets. Body-FirstEx corresponds to the set of features from transcribed sub-regions excluding the first exon. Upstream+FirstEx corresponds to the set of features from both the 2kb upstream region and the first exon.

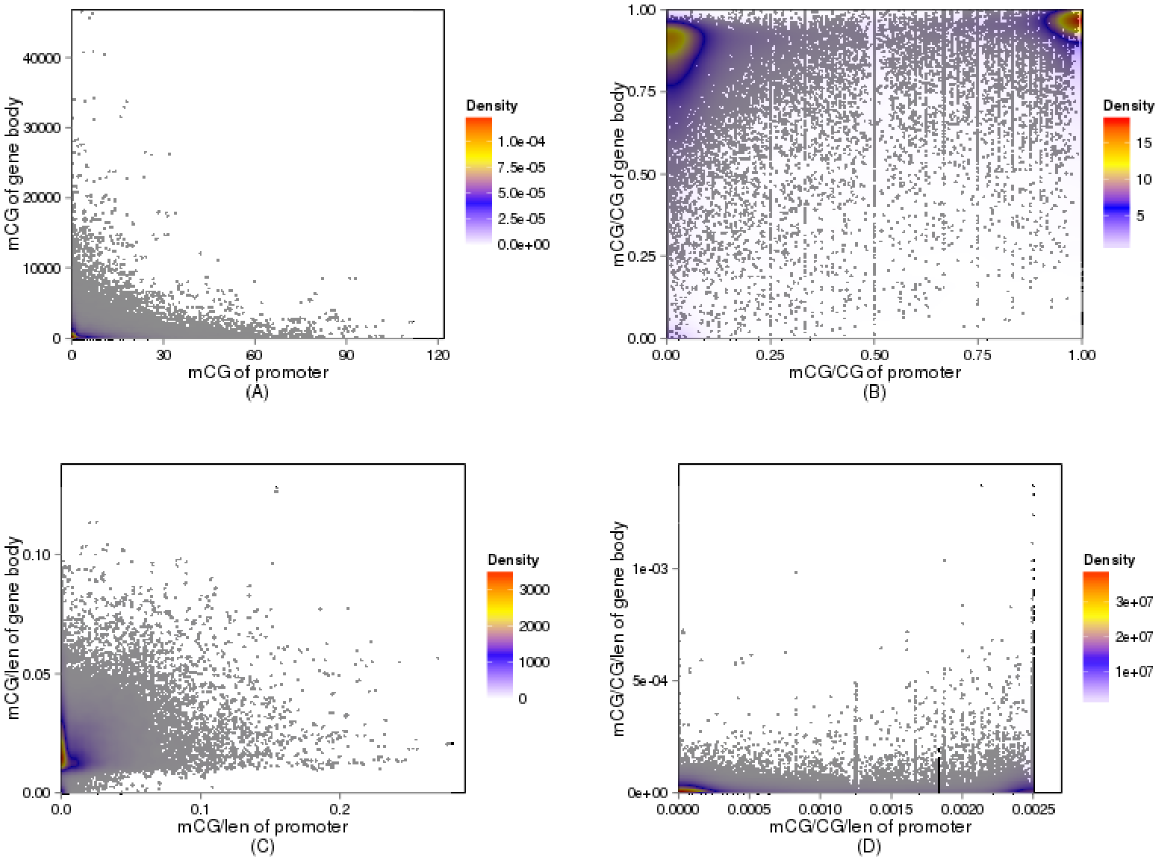

Figure S27: Relationship between DNA methylation at the upstream and transcribed region of transcripts. Each point in the figures corresponds to a transcript. The four panels show the plots based on different DNA methylation measures, namely mCG (A), mCG/CG (B), mCG/len (C) and mCG/CG/len (D). Color indicates local point density.

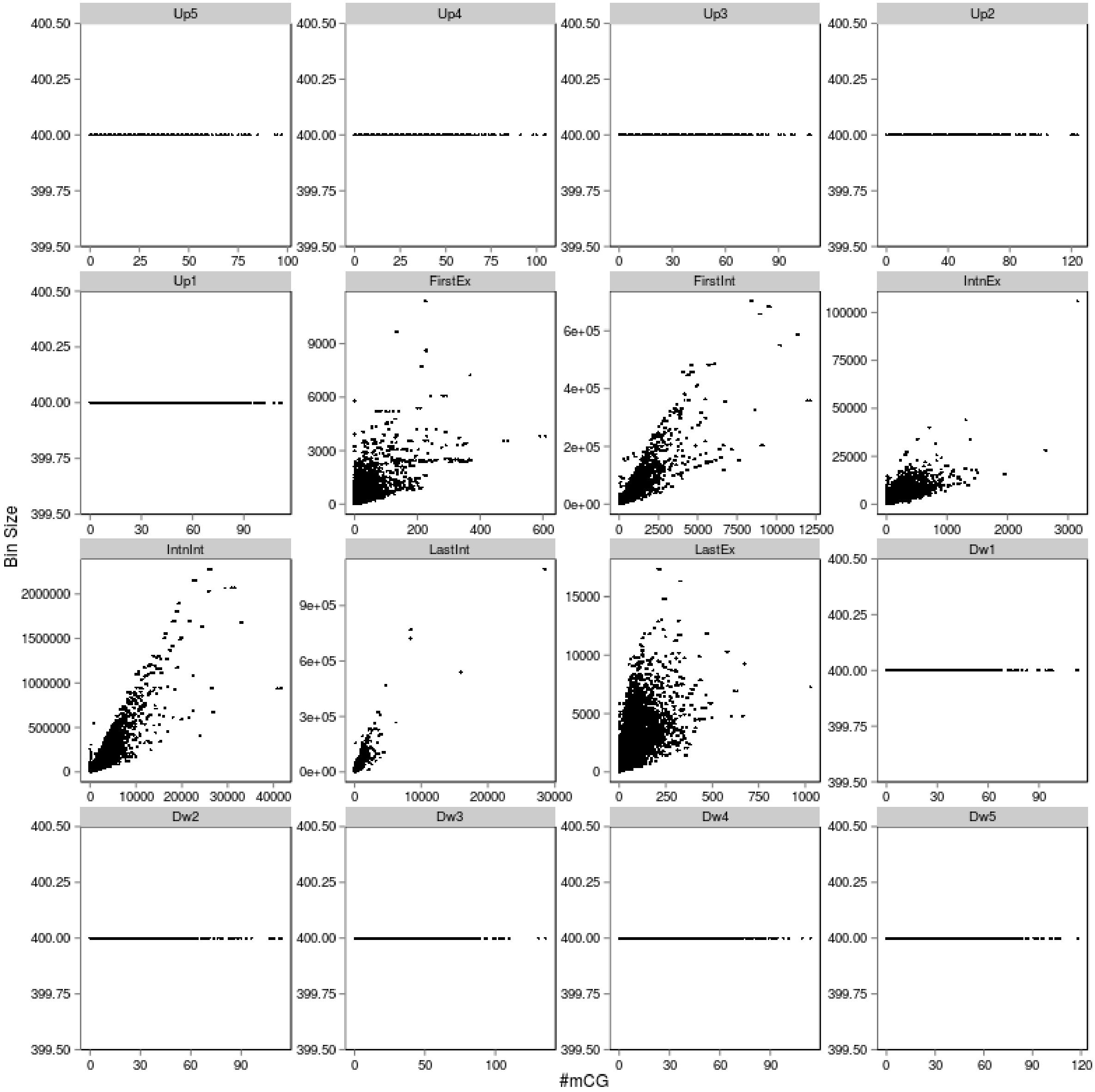

Figure S28: Scatterplots between number of methylated CpG sites (mCG, x-axis) and length of different sub-regions (y-axis) of genes. Each panel corresponds to one of the 16 sub-regions defined for a gene. Each point corresponds to one gene. For sub-regions of fixed lengths, including the upstream and downstream ones, the y-coordinates of the points are all equal.

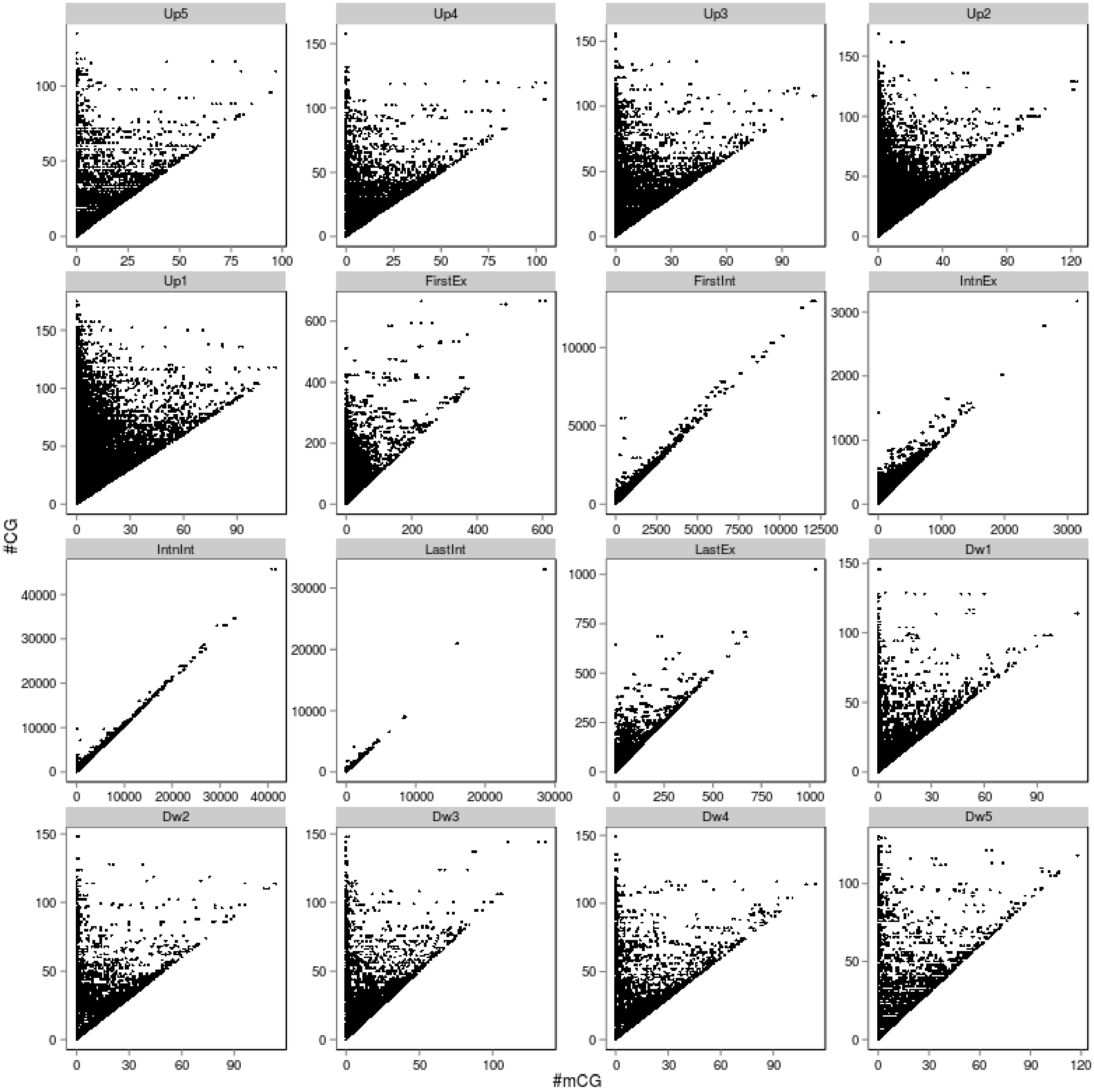

Figure S29: Scatterplots between number of methylated CpG sites (mCG, x-axis) and the total number of CpG sites (CG, x-axis) of genes. Each panel corresponds to one of the 16 sub-regions defined for a gene. Each point corresponds to one gene.

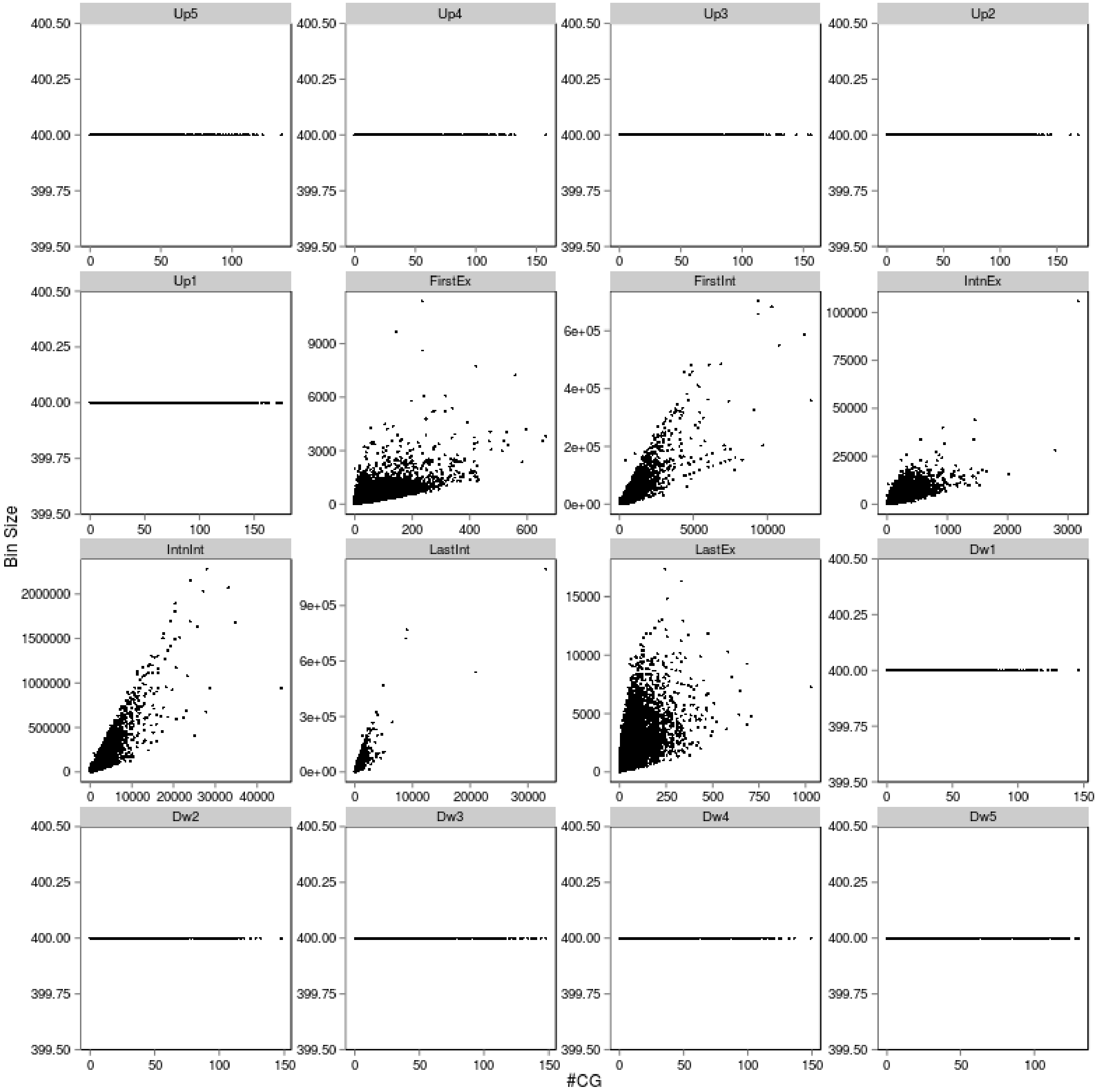

Figure S30: Scatterplots between total number of CpG sites (CG, x-axis) and length of different sub-regions (y-axis) of genes. Each panel corresponds to one of the 16 sub-regions defined for a gene. Each point corresponds to one gene. For sub-regions of fixed lengths, including the upstream and downstream ones, the y-coordinates of the points are all equal.

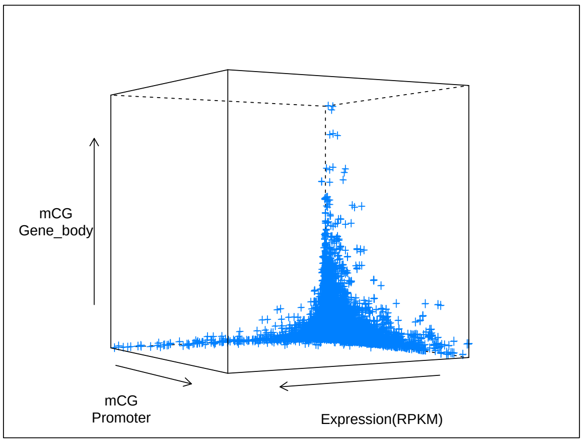

(a) mCG

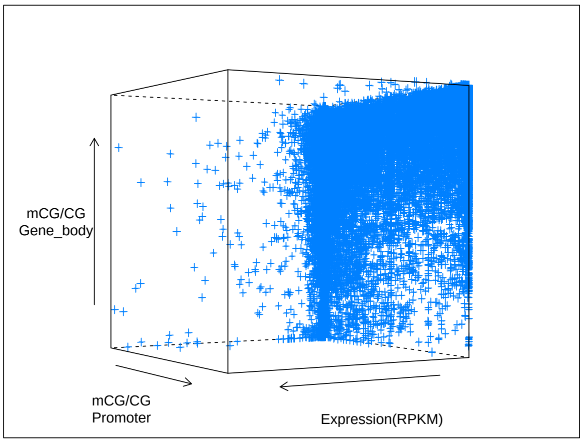

(b) mCG/CG

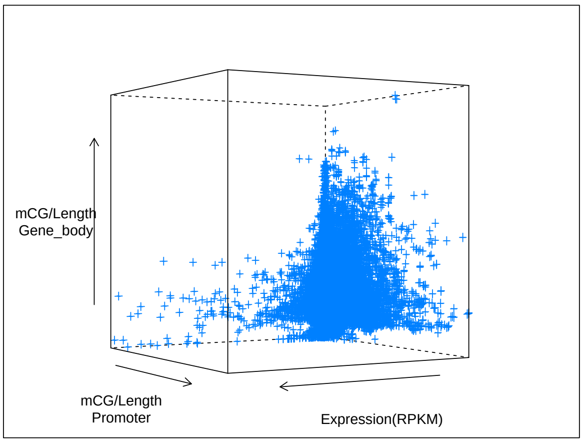

(c) mCG/len

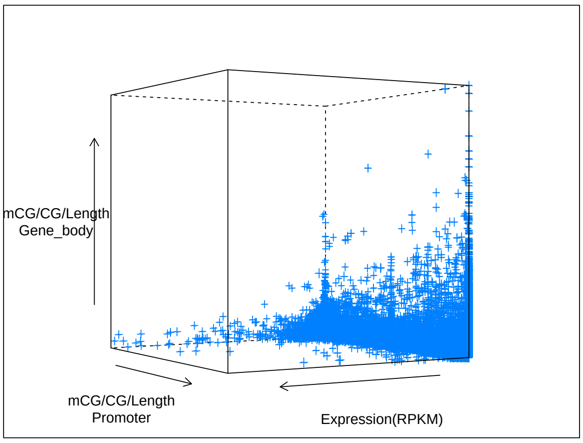

(d) mCG/CG/len

Figure S31: Relationship between promoter methylation, gene body methylation, and gene expression. Each point in the figures corresponds to a gene. The four panels show the plots based on different DNA methylation measures, namely mCG (A), mCG/CG (B), mCG/len (C) and mCG/CG/len (D).

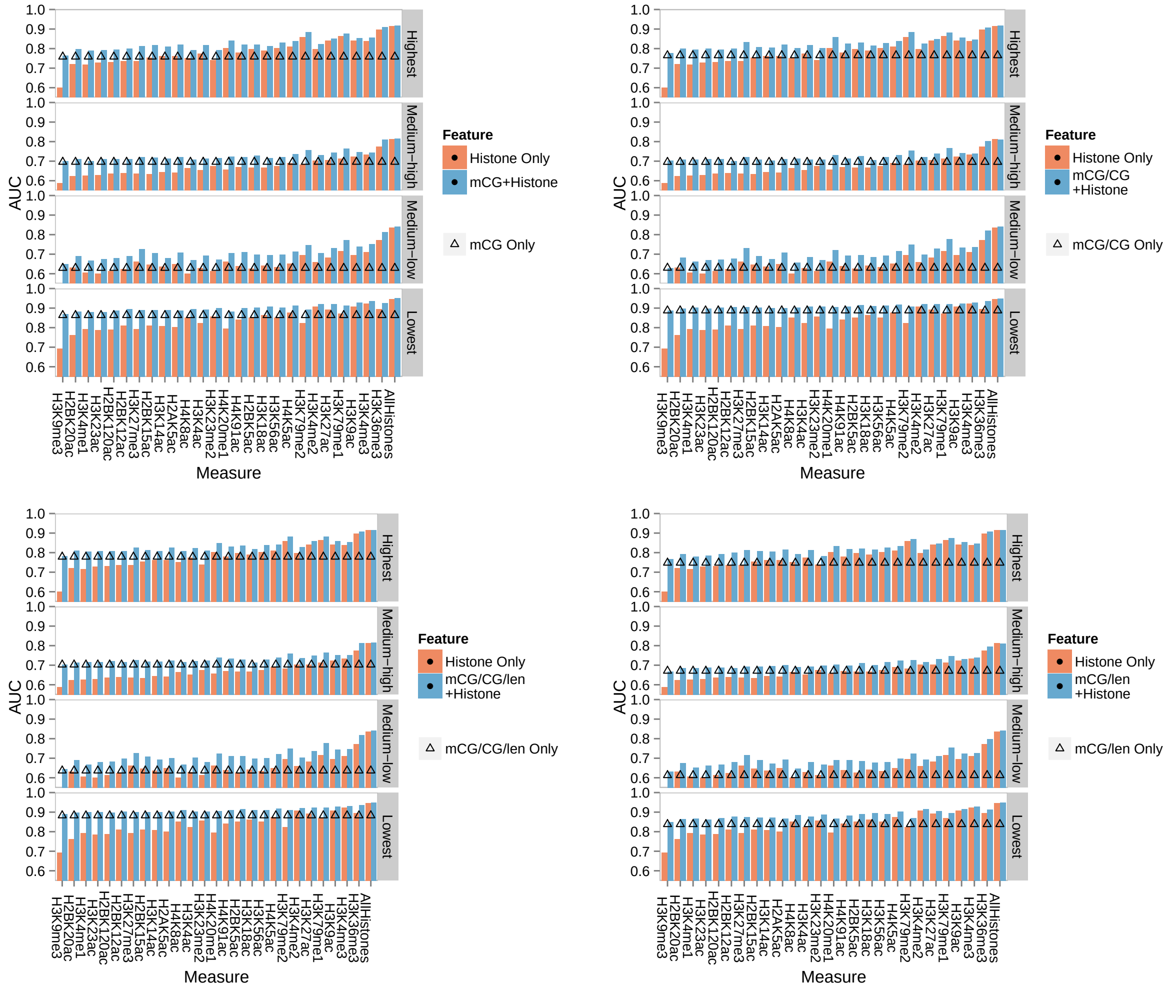

Figure S32: Joint effects of DNA methylation and histone modifications on each gene expression class. The four panels show the results based on the four DNA methylation measures. In each panel, there are four sub-panels showing results that involve genes from different expression classes. They compare the Random Forest expression models with only DNA methylation features (straight line with triangle markers), only histone modification features (orange bars), or both (blue bars). For DNA methylation and any type of histone modifications, its signal level is computed as the average over the upstream, transcribed and downstream regions of a gene. In each sub-panel, the first 26 bar groups correspond to models involving one of the 26 types of histone modification, while the last bar group corresponds to the model involving all 26 types of histone modification.

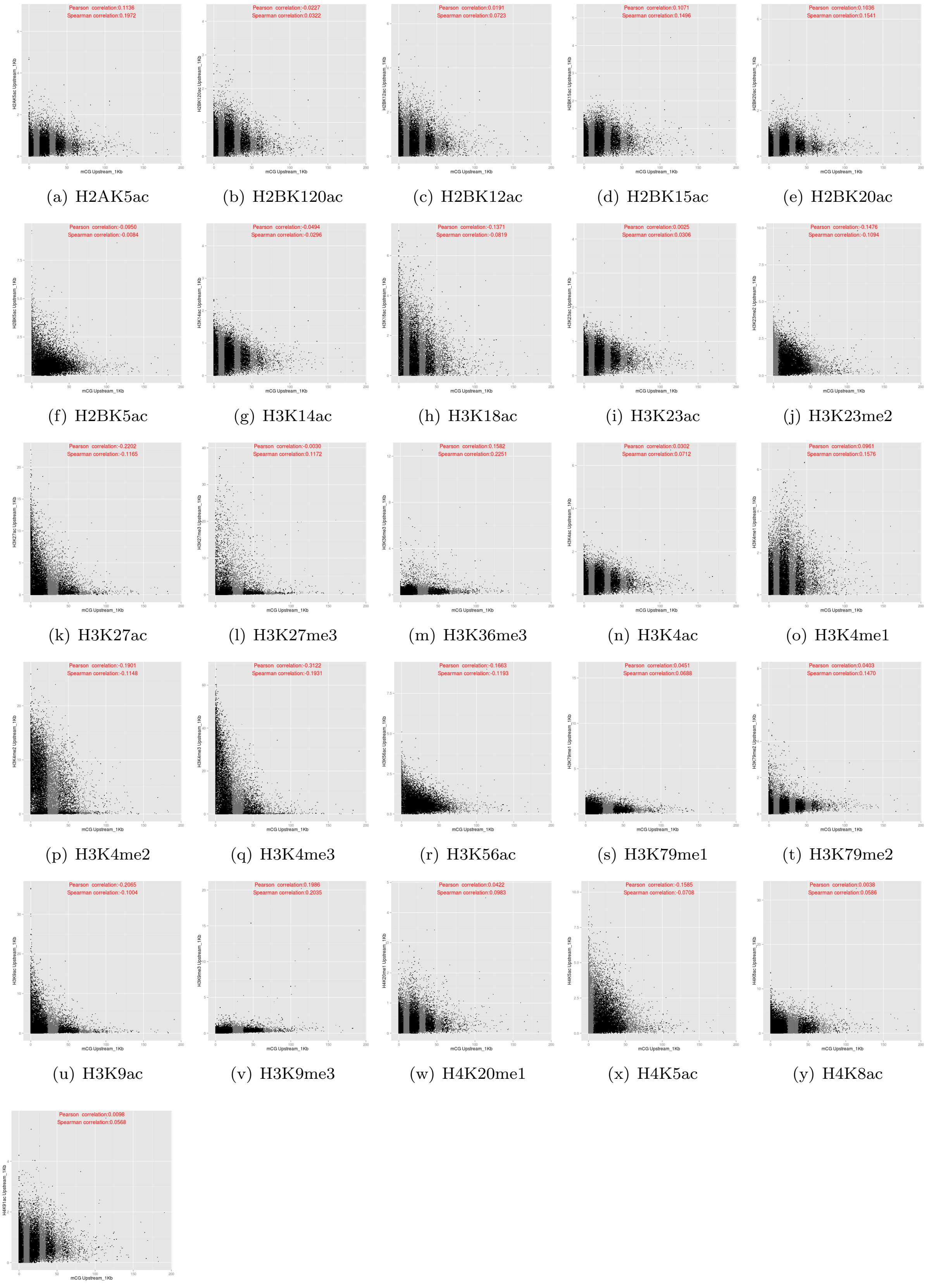

(a) H2AK5ac (b) H2BK120ac (c) H2BK12ac (d) H2BK15ac (e) H2BK20ac

(f) H2BK5ac (g) H3K14ac (h) H3K18ac (i) H3K23ac (j) H3K23me2

(k) H3K27ac (l) H3K27me3 (m) H3K36me3 (n) H3K4ac (o) H3K4me1

(p) H3K4me2 (q) H3K4me3 (r) H3K56ac (s) H3K79me1 (t) H3K79me2

(u) H3K9ac (v) H3K9me3 (w) H4K20me1 (x) H4K5ac (y) H4K8ac

(z) H4K91ac

Figure S33: Relationships between the DNA methylation (y-axis) and histone modification (x-axis), based on data from the upstream regions and the mCG DNA methylation measure. Each panel corresponds to one of the 26 types of histone modification.

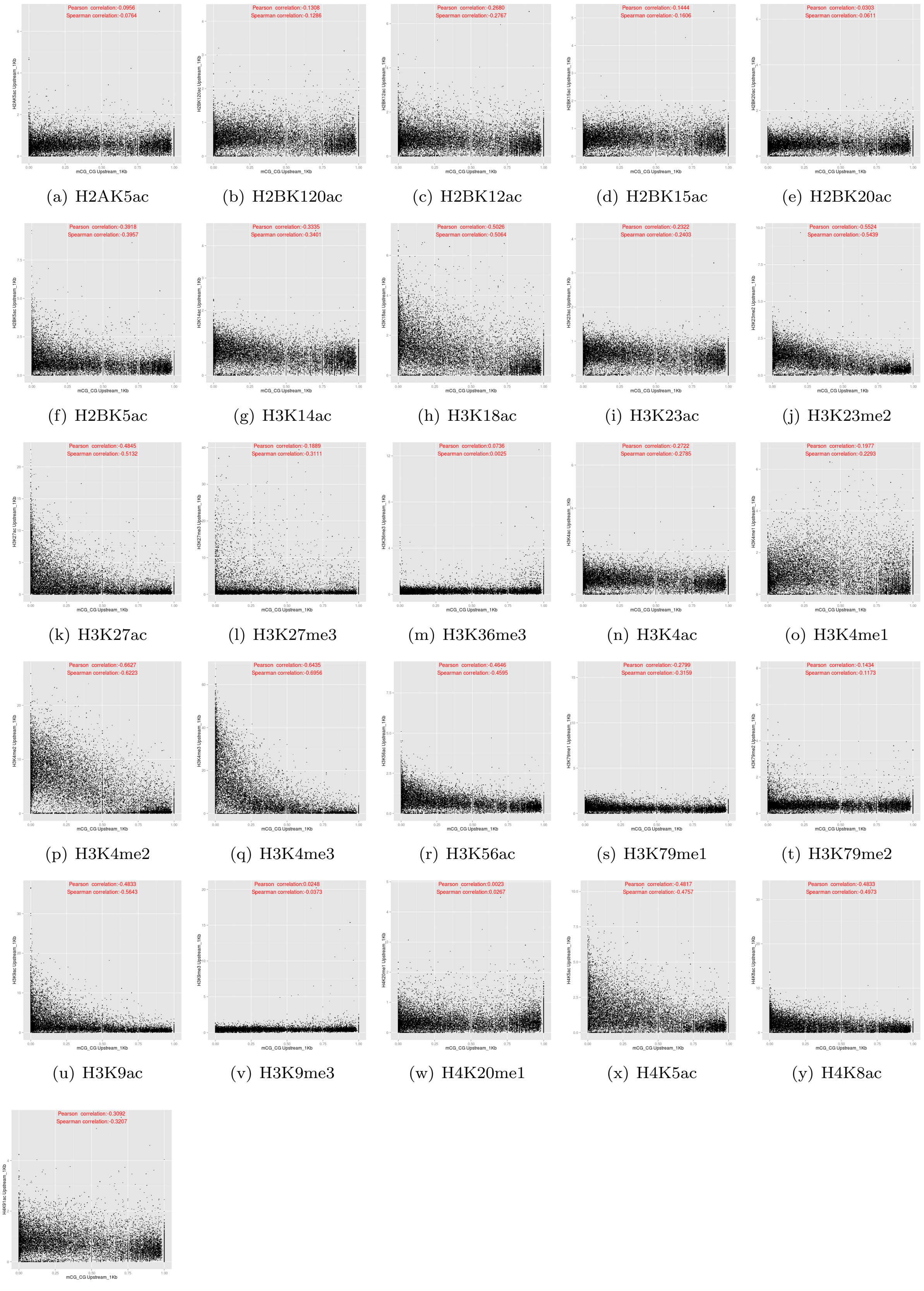

(a) H2AK5ac (b) H2BK120ac (c) H2BK12ac (d) H2BK15ac (e) H2BK20ac

(f) H2BK5ac (g) H3K14ac (h) H3K18ac (i) H3K23ac (j) H3K23me2

(k) H3K27ac (l) H3K27me3 (m) H3K36me3 (n) H3K4ac (o) H3K4me1

(p) H3K4me2 (q) H3K4me3 (r) H3K56ac (s) H3K79me1 (t) H3K79me2

(u) H3K9ac (v) H3K9me3 (w) H4K20me1 (x) H4K5ac (y) H4K8ac

(z) H4K91ac

Figure S34: Relationships between the DNA methylation (y-axis) and histone modification (x-axis), based on data from the upstream regions and the mCG/CG DNA methylation measure. Each panel corresponds to one of the 26 types of histone modification.

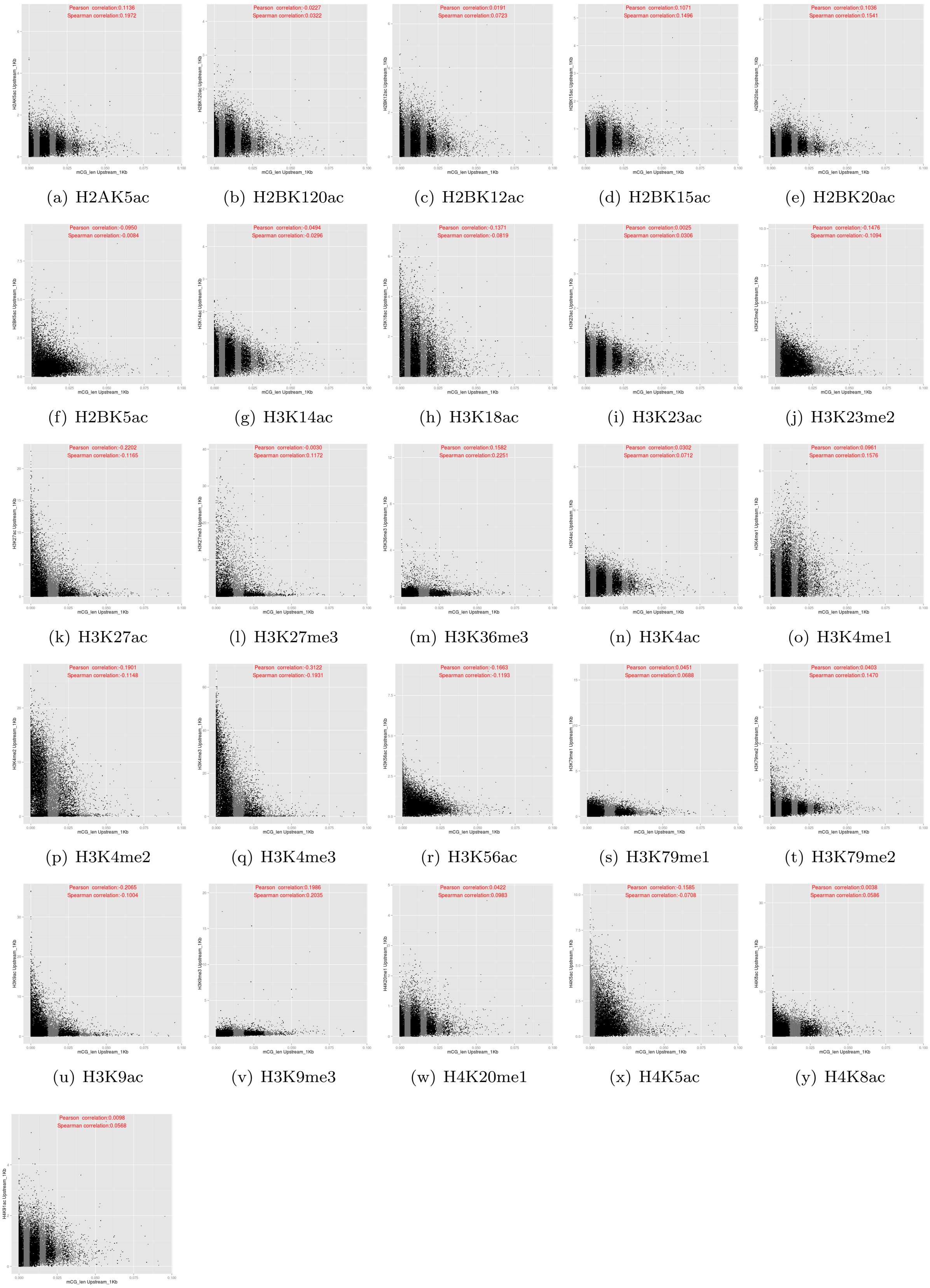

(a) H2AK5ac (b) H2BK120ac (c) H2BK12ac (d) H2BK15ac (e) H2BK20ac

(f) H2BK5ac (g) H3K14ac (h) H3K18ac (i) H3K23ac (j) H3K23me2

(k) H3K27ac (l) H3K27me3 (m) H3K36me3 (n) H3K4ac (o) H3K4me1

(p) H3K4me2 (q) H3K4me3 (r) H3K56ac (s) H3K79me1 (t) H3K79me2

(u) H3K9ac (v) H3K9me3 (w) H4K20me1 (x) H4K5ac (y) H4K8ac

(z) H4K91ac

Figure S35: Relationships between the DNA methylation (y-axis) and histone modification (x-axis), based on data from the upstream regions and the mCG/len DNA methylation measure. Each panel corresponds to one of the 26 types of histone modification.

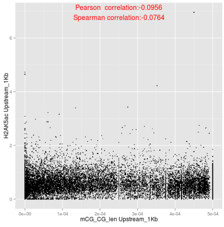

(a) H2AK5ac

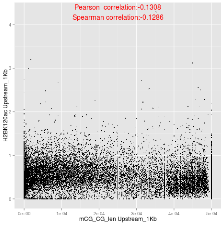

(b) H2BK120ac

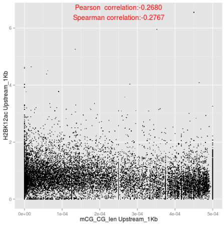

(c) H2BK12ac

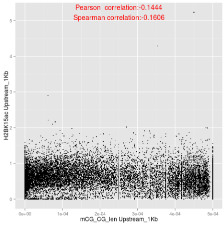

(d) H2BK15ac

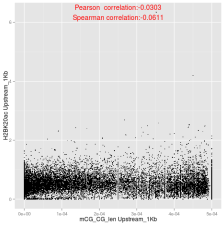

(e) H2BK20ac

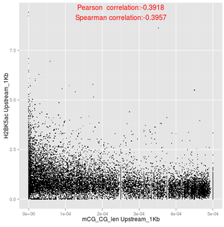

(f) H2BK5ac

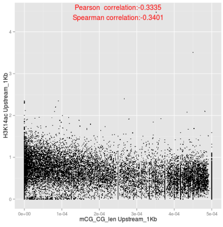

(g) H3K14ac

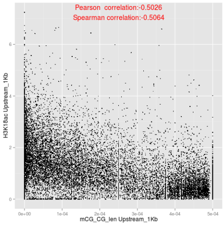

(h) H3K18ac

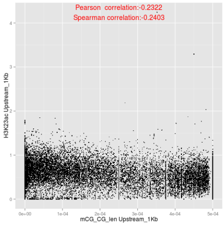

(i) H3K23ac

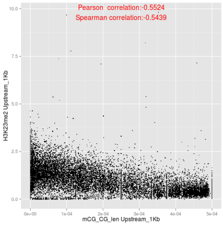

(j) H3K23me2

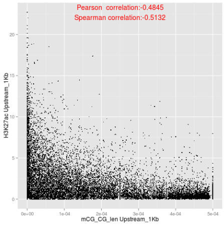

(k) H3K27ac

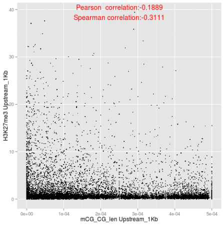

(l) H3K27me3

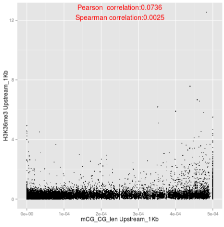

(m) H3K36me3

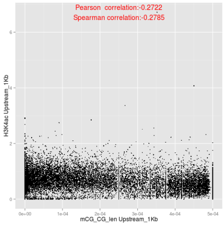

(n) H3K4ac

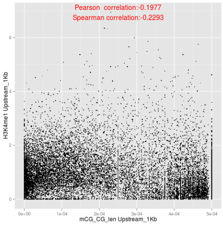

(o) H3K4me1

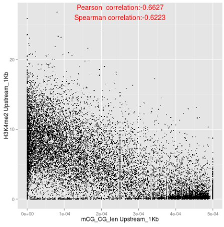

(p) H3K4me2

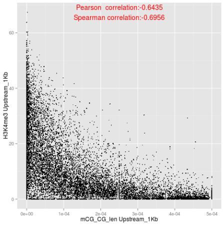

(q) H3K4me3

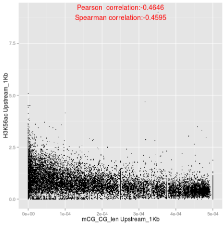

(r) H3K56ac

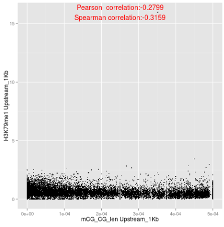

(s) H3K79me1

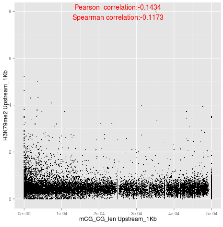

(t) H3K79me2

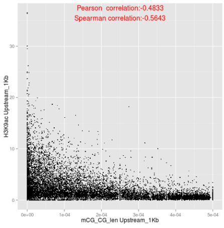

(u) H3K9ac

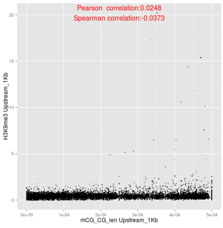

(v) H3K9me3

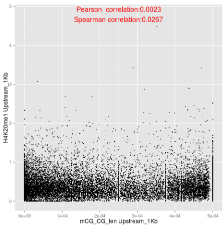

(w) H4K20me1

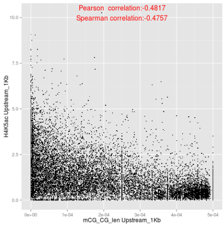

(x) H4K5ac

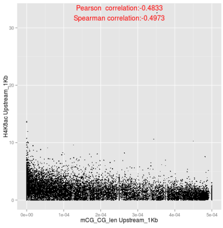

(y) H4K8ac

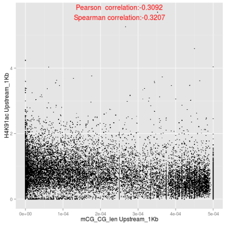

(z) H4K91ac

Figure S36: Relationships between the DNA methylation (y-axis) and histone modification (x-axis), based on data from the upstream regions and the mCG/CG/len DNA methylation measure. Each panel corresponds to one of the 26 types of histone modification.

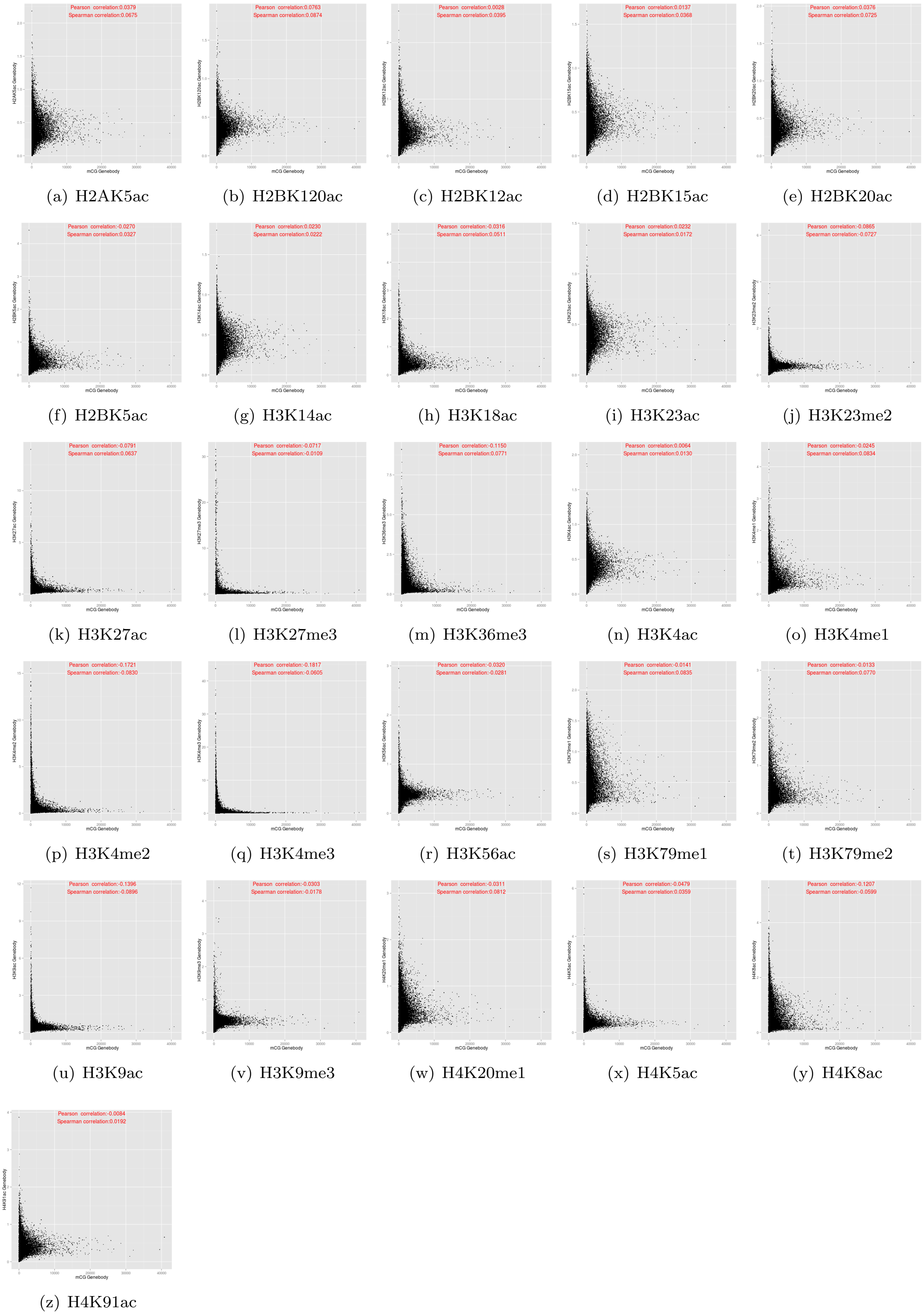

(a) H2AK5ac (b) H2BK120ac (c) H2BK12ac (d) H2BK15ac (e) H2BK20ac

(f) H2BK5ac (g) H3K14ac (h) H3K18ac (i) H3K23ac (j) H3K23me2

(k) H3K27ac (l) H3K27me3 (m) H3K36me3 (n) H3K4ac (o) H3K4me1

(p) H3K4me2 (q) H3K4me3 (r) H3K56ac (s) H3K79me1 (t) H3K79me2

(u) H3K9ac (v) H3K9me3 (w) H4K20me1 (x) H4K5ac (y) H4K8ac

(z) H4K91ac

Figure S37: Relationships between the DNA methylation (y-axis) and histone modification (x-axis), based on data from the transcribed regions and the mCG DNA methylation measure. Each panel corresponds to one of the 26 types of histone modification.

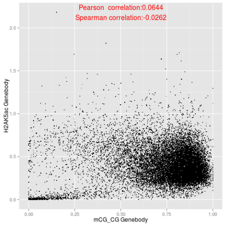

(a) H2AK5ac

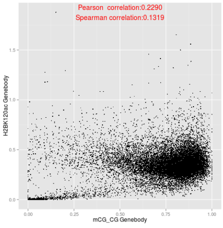

(b) H2BK120ac

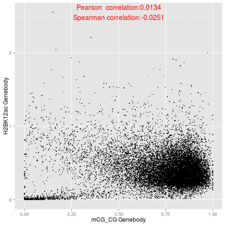

(c) H2BK12ac

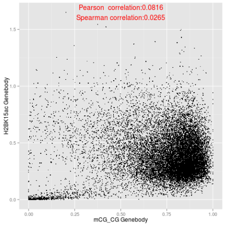

(d) H2BK15ac

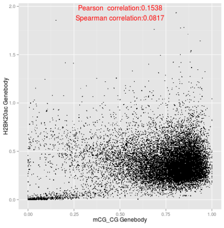

(e) H2BK20ac

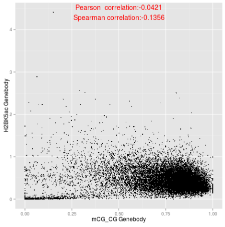

(f) H2BK5ac

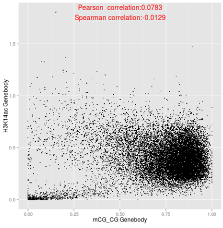

(g) H3K14ac

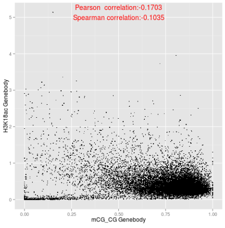

(h) H3K18ac

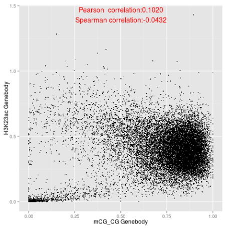

(i) H3K23ac

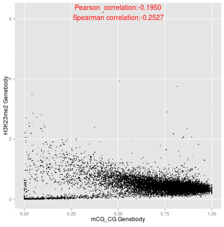

(j) H3K23me2

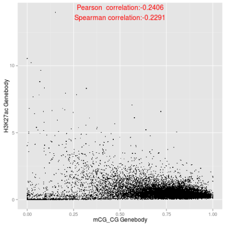

(k) H3K27ac

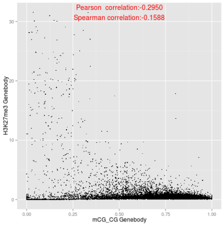

(l) H3K27me3

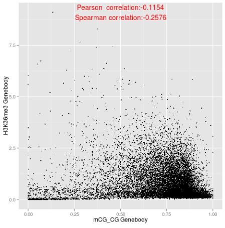

(m) H3K36me3

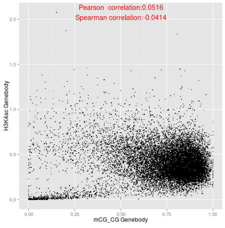

(n) H3K4ac

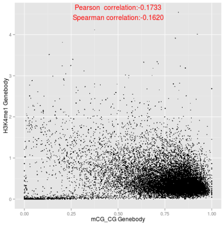

(o) H3K4me1

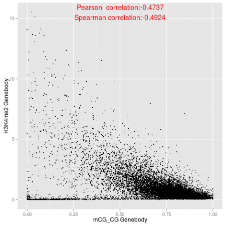

(p) H3K4me2

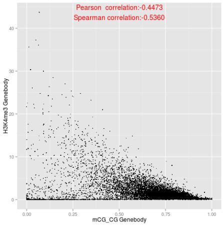

(q) H3K4me3

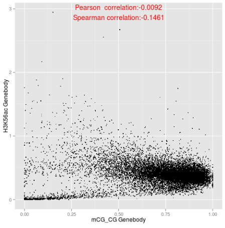

(r) H3K56ac

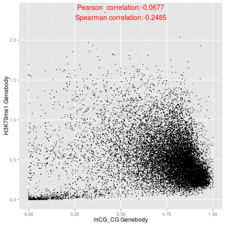

(s) H3K79me1

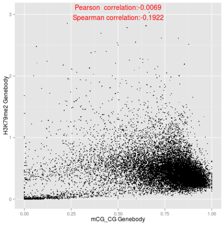

(t) H3K79me2

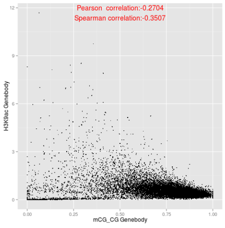

(u) H3K9ac

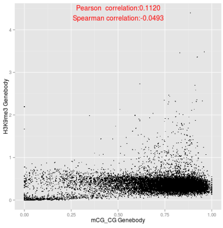

(v) H3K9me3

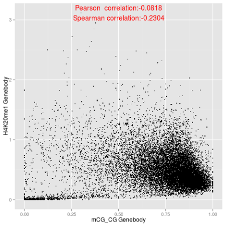

(w) H4K20me1

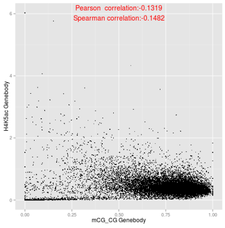

(x) H4K5ac

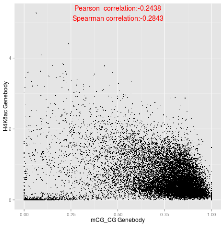

(y) H4K8ac

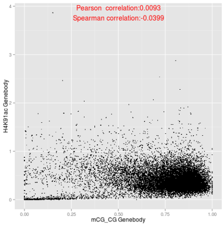

(z) H4K91ac

Figure S38: Relationships between the DNA methylation (y-axis) and histone modification (x-axis), based on data from the transcribed regions and the mCG/CG DNA methylation measure. Each panel corresponds to one of the 26 types of histone modification.

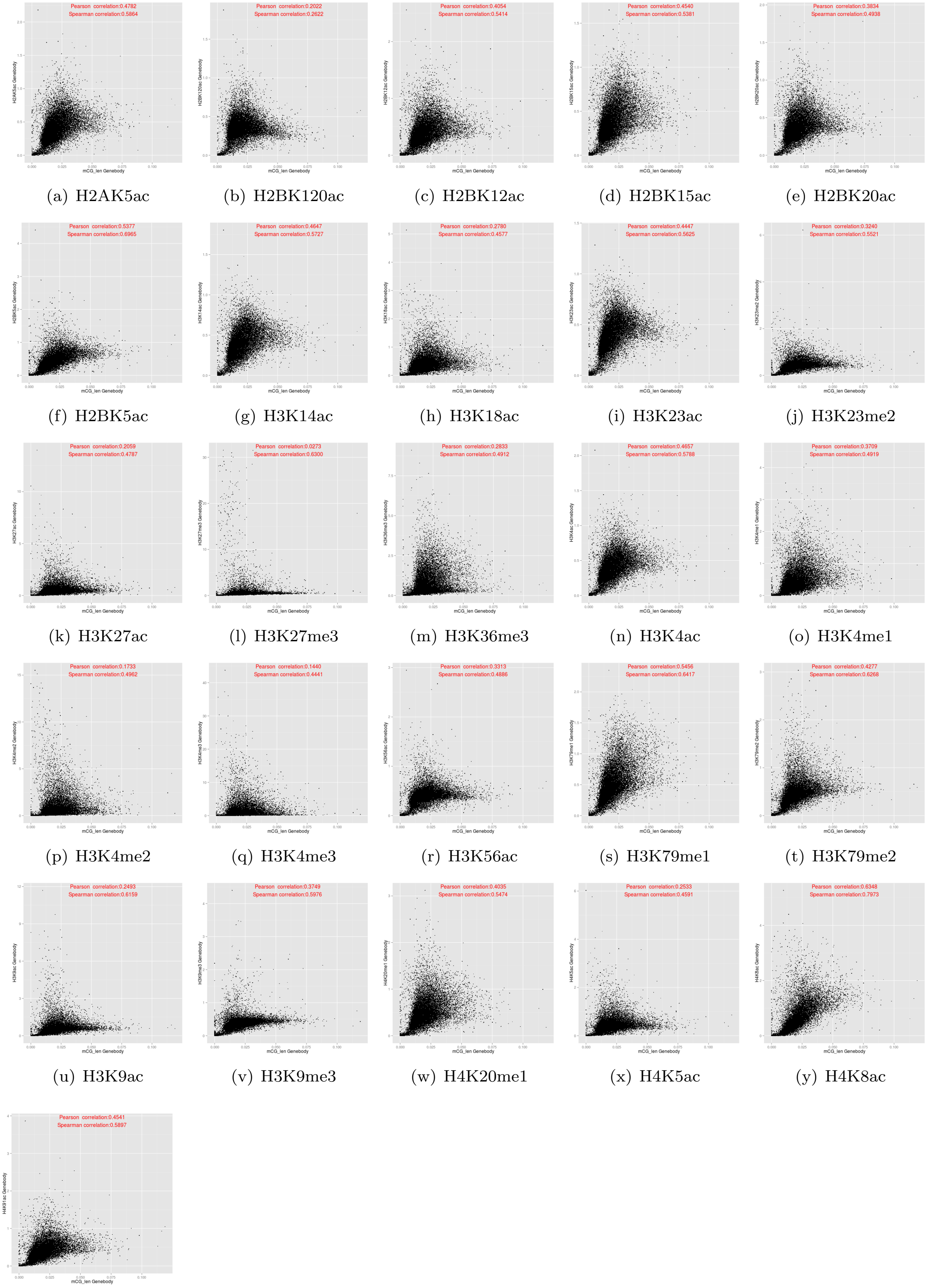

(a) H2AK5ac (b) H2BK120ac (c) H2BK12ac (d) H2BK15ac (e) H2BK20ac

(f) H2BK5ac (g) H3K14ac (h) H3K18ac (i) H3K23ac (j) H3K23me2

(k) H3K27ac (l) H3K27me3 (m) H3K36me3 (n) H3K4ac (o) H3K4me1

(p) H3K4me2 (q) H3K4me3 (r) H3K56ac (s) H3K79me1 (t) H3K79me2

(u) H3K9ac (v) H3K9me3 (w) H4K20me1 (x) H4K5ac (y) H4K8ac

(z) H4K91ac

Figure S39: Relationships between the DNA methylation (y-axis) and histone modification (x-axis), based on data from the transcribed regions and the mCG/len DNA methylation measure. Each panel corresponds to one of the 26 types of histone modification.

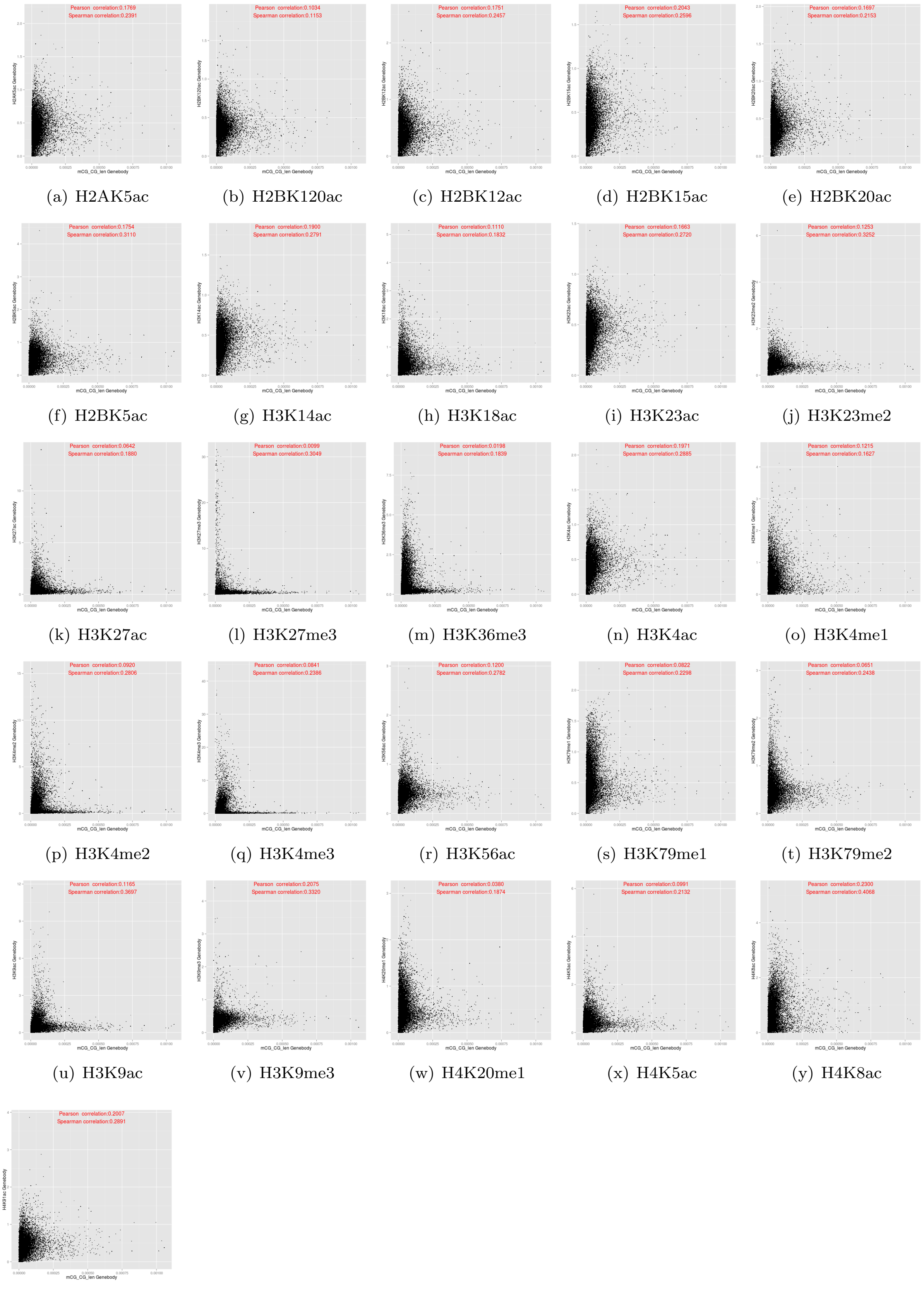

(a) H2AK5ac (b) H2BK120ac (c) H2BK12ac (d) H2BK15ac (e) H2BK20ac

(f) H2BK5ac (g) H3K14ac (h) H3K18ac (i) H3K23ac (j) H3K23me2

(k) H3K27ac (l) H3K27me3 (m) H3K36me3 (n) H3K4ac (o) H3K4me1

(p) H3K4me2 (q) H3K4me3 (r) H3K56ac (s) H3K79me1 (t) H3K79me2

(u) H3K9ac (v) H3K9me3 (w) H4K20me1 (x) H4K5ac (y) H4K8ac

(z) H4K91ac

Figure S40: Relationships between the DNA methylation (y-axis) and histone modification (x-axis), based on data from the transcribed regions and the mCG/CG/len DNA methylation measure. Each panel corresponds to one of the 26 types of histone modification.

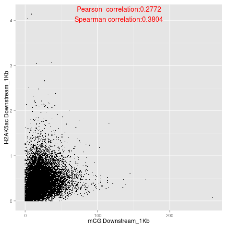

(a) H2AK5ac

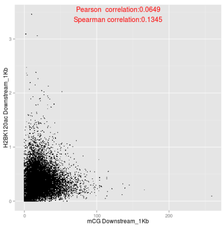

(b) H2BK120ac

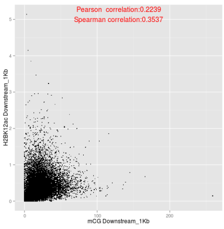

(c) H2BK12ac

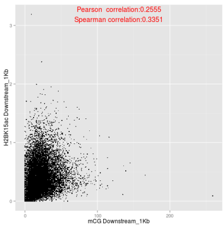

(d) H2BK15ac

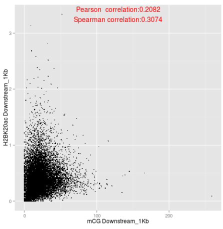

(e) H2BK20ac

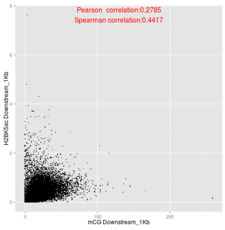

(f) H2BK5ac

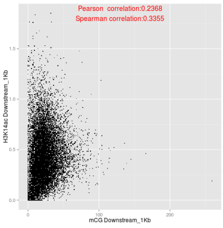

(g) H3K14ac

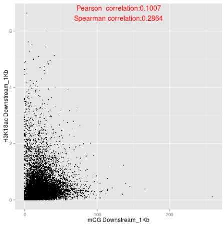

(h) H3K18ac

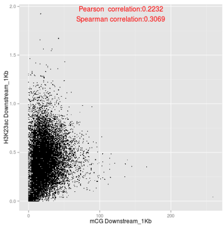

(i) H3K23ac

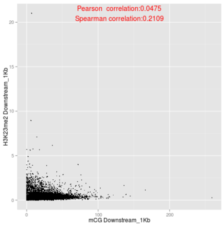

(j) H3K23me2

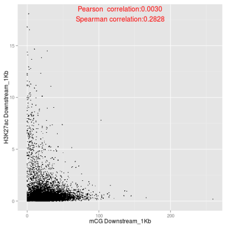

(k) H3K27ac

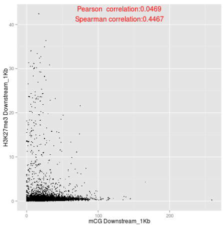

(l) H3K27me3

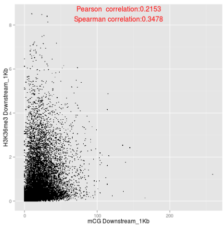

(m) H3K36me3

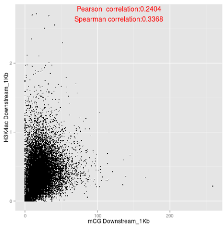

(n) H3K4ac

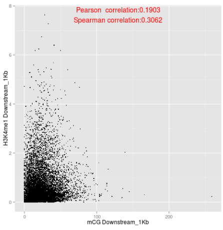

(o) H3K4me1

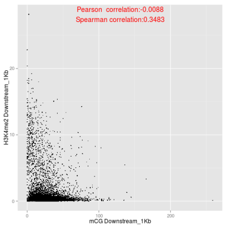

(p) H3K4me2

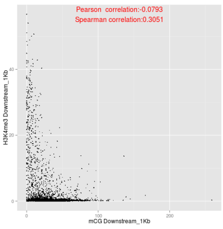

(q) H3K4me3

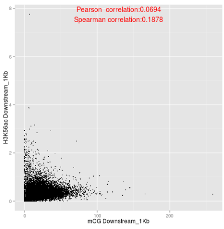

(r) H3K56ac

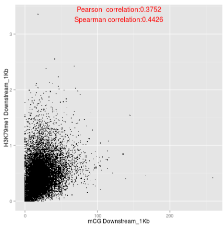

(s) H3K79me1

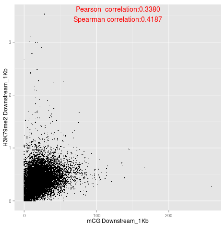

(t) H3K79me2

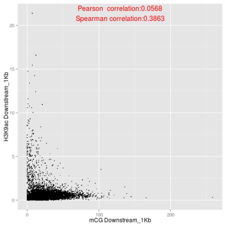

(u) H3K9ac

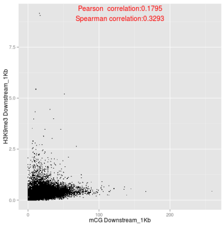

(v) H3K9me3

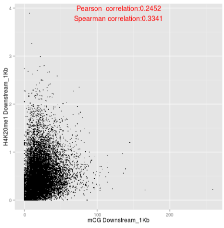

(w) H4K20me1

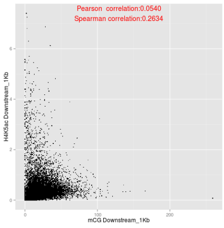

(x) H4K5ac

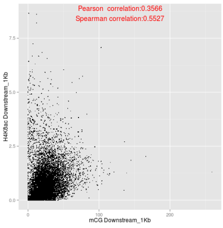

(y) H4K8ac

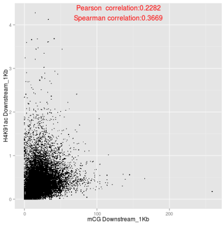

(z) H4K91ac

Figure S41: Relationships between the DNA methylation (y-axis) and histone modification (x-axis), based on data from the downstream regions and the mCG DNA methylation measure. Each panel corresponds to one of the 26 types of histone modification.

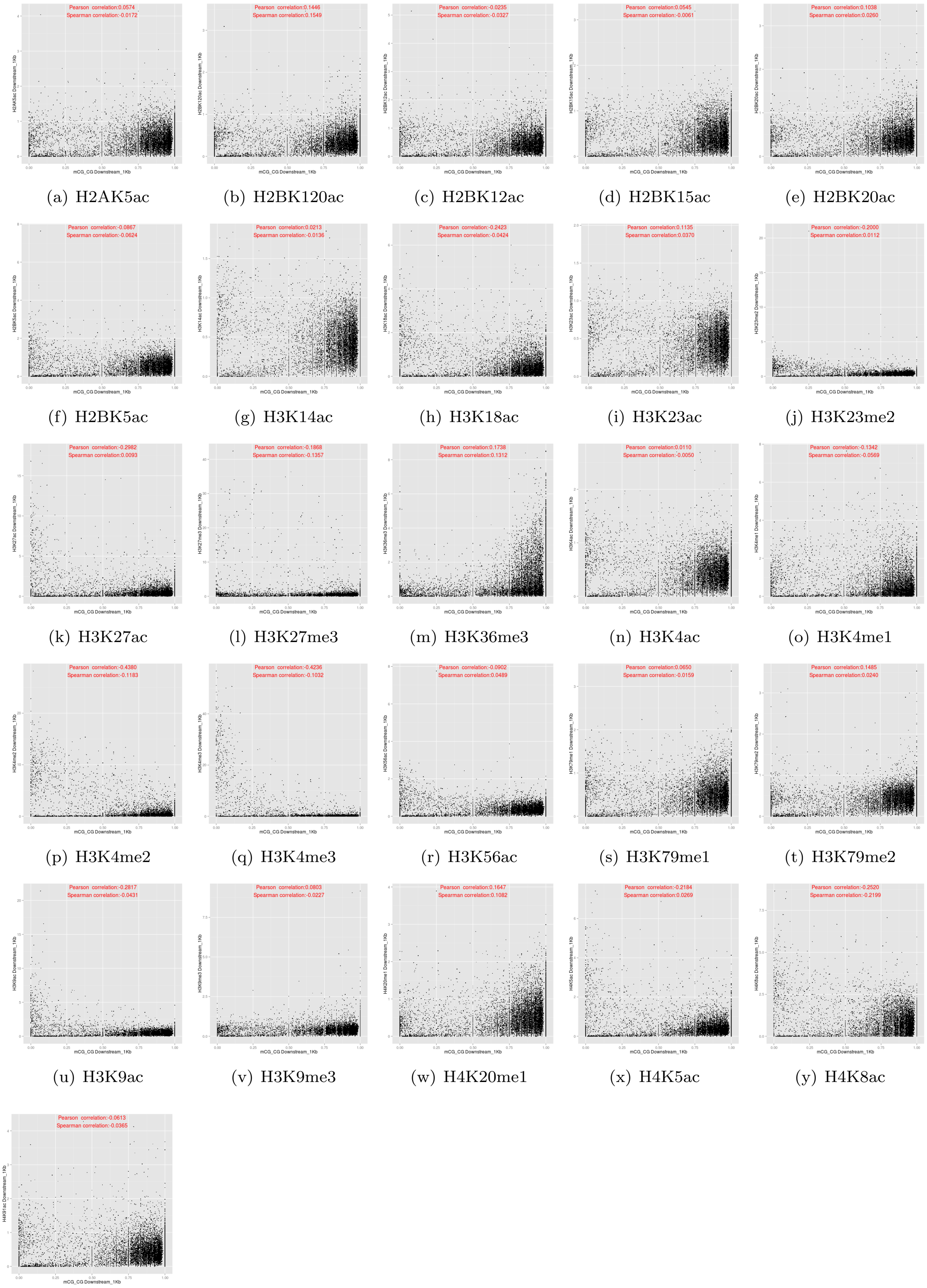

(a) H2AK5ac (b) H2BK120ac (c) H2BK12ac (d) H2BK15ac (e) H2BK20ac

(f) H2BK5ac (g) H3K14ac (h) H3K18ac (i) H3K23ac (j) H3K23me2

(k) H3K27ac (l) H3K27me3 (m) H3K36me3 (n) H3K4ac (o) H3K4me1

(p) H3K4me2 (q) H3K4me3 (r) H3K56ac (s) H3K79me1 (t) H3K79me2

(u) H3K9ac (v) H3K9me3 (w) H4K20me1 (x) H4K5ac (y) H4K8ac

(z) H4K91ac

Figure S42: Relationships between the DNA methylation (y-axis) and histone modification (x-axis), based on data from the downstream regions and the mCG/CG DNA methylation measure. Each panel corresponds to one of the 26 types of histone modification.

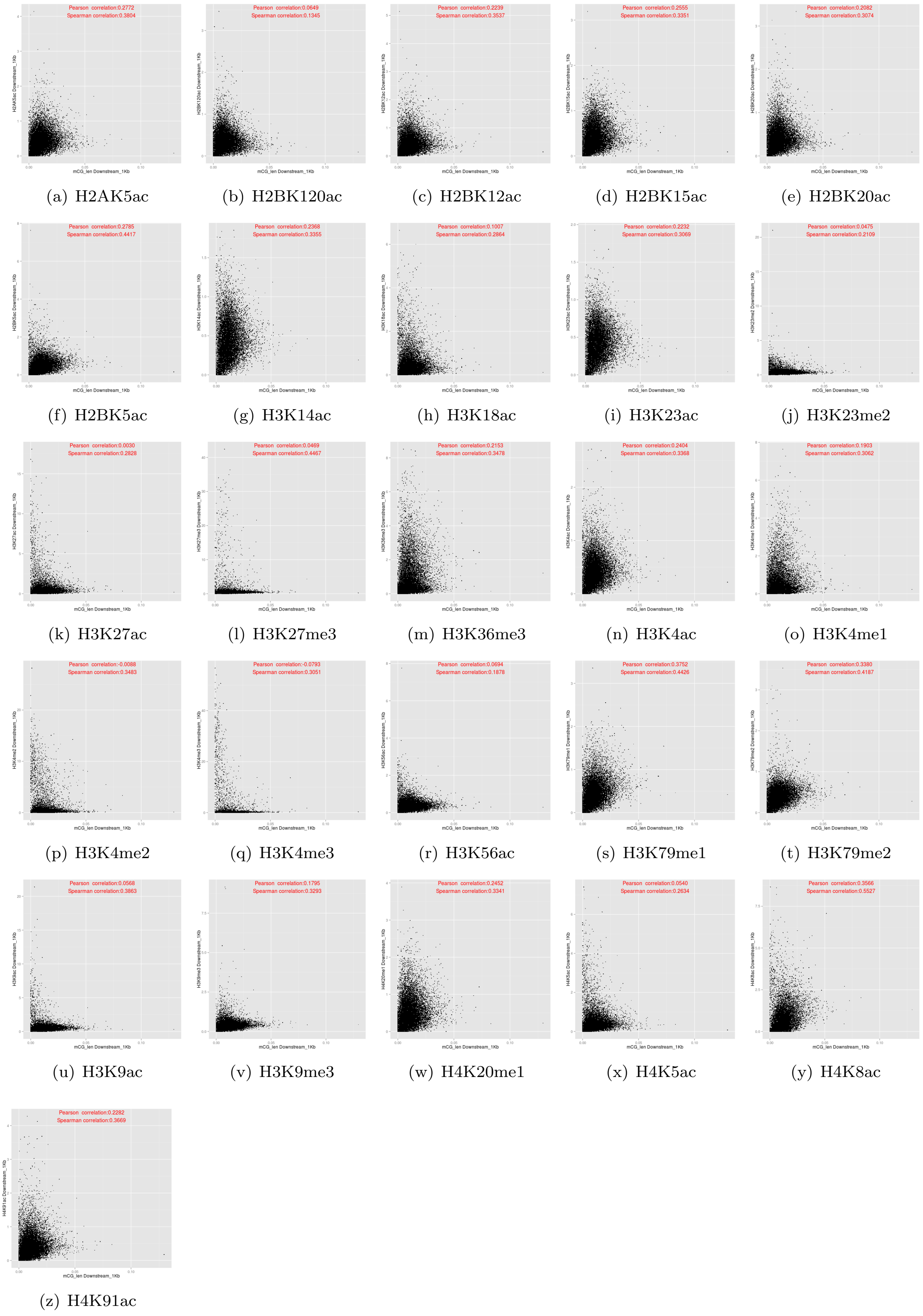

(a) H2AK5ac (b) H2BK120ac (c) H2BK12ac (d) H2BK15ac (e) H2BK20ac

(f) H2BK5ac (g) H3K14ac (h) H3K18ac (i) H3K23ac (j) H3K23me2

(k) H3K27ac (l) H3K27me3 (m) H3K36me3 (n) H3K4ac (o) H3K4me1

(p) H3K4me2 (q) H3K4me3 (r) H3K56ac (s) H3K79me1 (t) H3K79me2

(u) H3K9ac (v) H3K9me3 (w) H4K20me1 (x) H4K5ac (y) H4K8ac

(z) H4K91ac

Figure S43: Relationships between the DNA methylation (y-axis) and histone modification (x-axis), based on data from the downstream regions and the mCG/len DNA methylation measure. Each panel corresponds to one of the 26 types of histone modification.

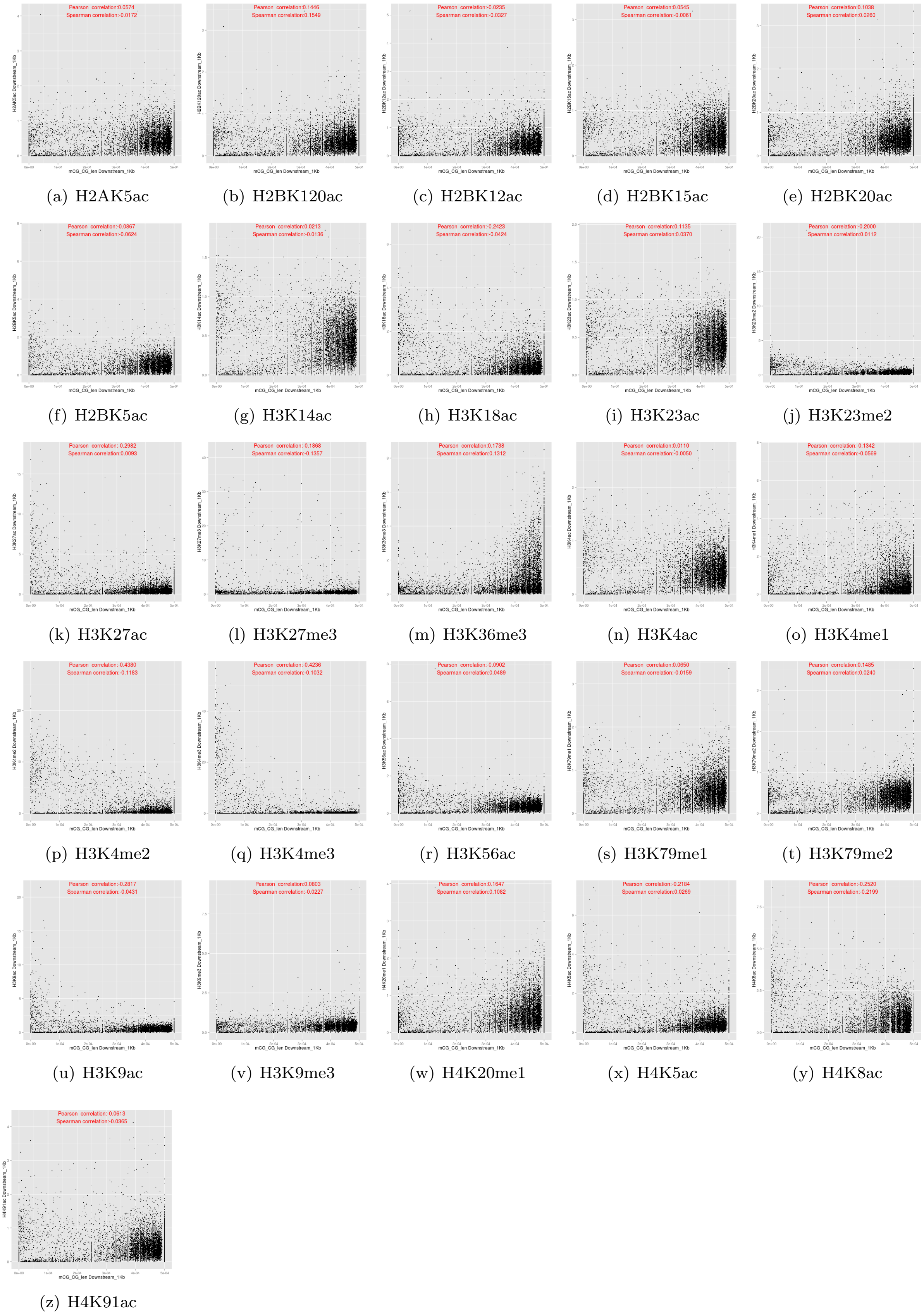

(a) H2AK5ac (b) H2BK120ac (c) H2BK12ac (d) H2BK15ac (e) H2BK20ac

(f) H2BK5ac (g) H3K14ac (h) H3K18ac (i) H3K23ac (j) H3K23me2

(k) H3K27ac (l) H3K27me3 (m) H3K36me3 (n) H3K4ac (o) H3K4me1

(p) H3K4me2 (q) H3K4me3 (r) H3K56ac (s) H3K79me1 (t) H3K79me2

(u) H3K9ac (v) H3K9me3 (w) H4K20me1 (x) H4K5ac (y) H4K8ac

(z) H4K91ac

Figure S44: Relationships between the DNA methylation (y-axis) and histone modification (x-axis), based on data from the downstream regions and the mCG/CG/len DNA methylation measure. Each panel corresponds to one of the 26 types of histone modification.

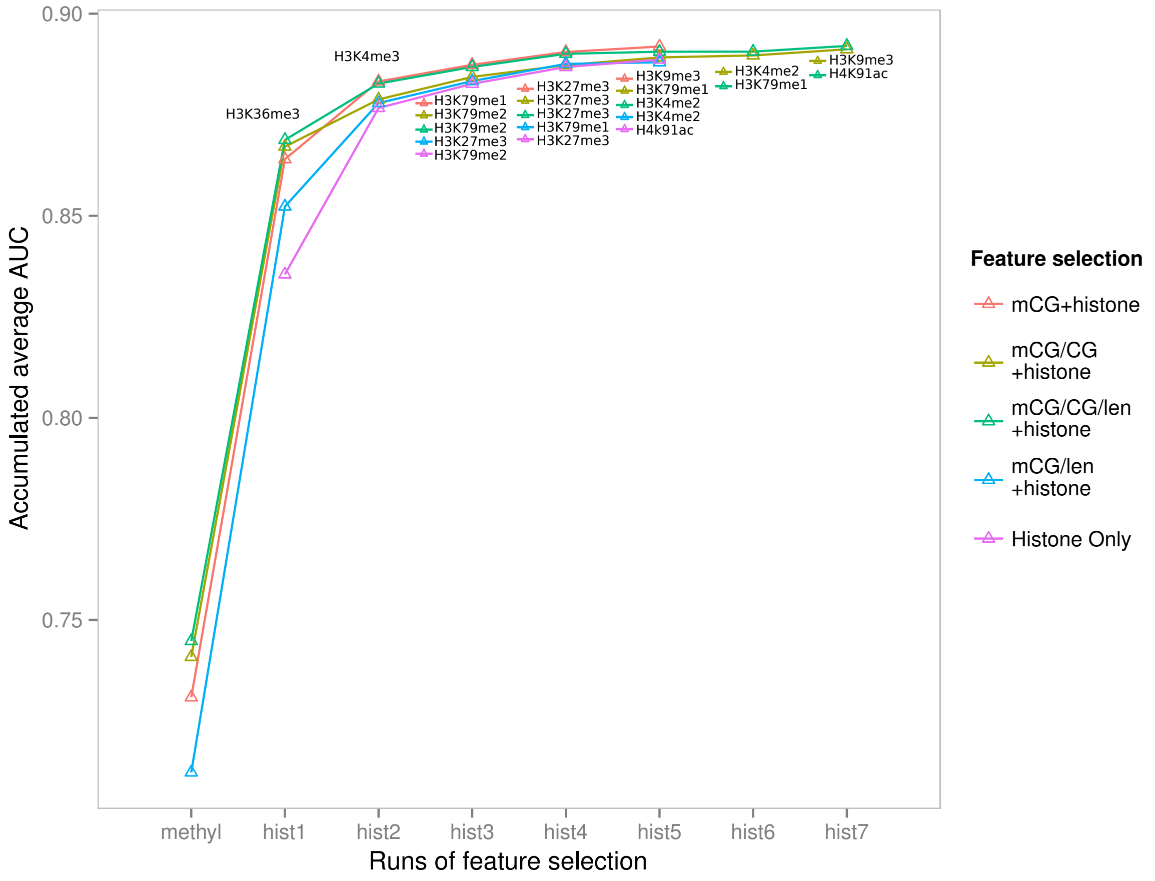

Figure S45: Feature selection for finding smallest sets of features with maximal modeling accuracy. Each curve shows the change of accuracy of the models by adding the next best feature set computed from all 16 sub-regions of genes. In the first four curves, the first feature set is fixed at DNA methylation based on one of the quantification measures. The fifth curve shows the results when DNA methylation features are not included. In all cases, the remaining feature sets are derived from histone modifications, where hist1 is the first type of histone modification that can maximize the accuracy gain, hist2 is the one that can maximize the accuracy gain after the first one has been added, and so on.
